# A dynamical systems model of emotional contagion: Oscillatory coupling in the circumplex of affect

**DOI:** 10.1371/journal.pone.0334738

**Published:** 2026-09-15

**Authors:** Diego Alexander Garzón-Alvarado, Carlos Alberto Duque-Daza, Alessio Alexiadis, Ricardo Mauricio Tamayo

**Affiliations:** 1 Biotechnology Institute, Universidad Nacional de Colombia, Bogotá, Colombia; 2 Research Group in Modelling and Numerical Methods in Engineering - GNUM, Universidad Nacional de Colombia, Bogotá, Colombia; 3 Department of Mechanical and Mechatronics Engineering, Universidad Nacional de Colombia, Bogotá, Colombia; 4 School of Chemical Engineering, University of Birmingham, Birmingham, United Kingdom; 5 Department of Psychology, Universidad Nacional de Colombia, Bogotá, Colombia; Universita degli Studi di Pisa, ITALY

## Abstract

**Background:**

Emotional contagion plays a crucial role in shaping group dynamics, interpersonal relationships, and affective regulation. Mathematical models of affect and agent-based models of emotional contagion are well established, but fewer formulations combine a continuous valence–arousal representation, explicit multi-agent coupling, time-continuous interaction dynamics, and mechanically interpretable dynamical parameters within a single framework.

**Objective:**

This study develops a mathematical and computational model of emotional contagion that integrates intra-individual affective dynamics and inter-individual affective influence within a time-dependent framework based on Russell’s Circumplex Model of Affect.

**Methods:**

Emotions are modeled as phasors in the complex plane, where each state evolves through the interaction of two oscillators: a radial oscillator for emotional intensity and an angular oscillator for affective direction. Emotional influence among agents is implemented through coupling matrices that allow dynamic simulation of contagion. The full system is expressed as a system of coupled first-order differential equations and solved numerically using Runge–Kutta integration schemes.

**Results:**

The simulations generated qualitative patterns of phase alignment, persistent state dispersion, leader-induced tracking, oscillatory amplification, and collective convergence under selected parameter and network configurations. They illustrate how attractors, damping, coupling strength, and network topology influence trajectories within the proposed mathematical framework.

**Conclusion:**

The proposed model offers a theoretically grounded and computationally tractable approach for studying emotional contagion and it should be regarded as an exploratory and hypothesis-generating computational framework. It has not been empirically calibrated or validated as a predictive or neurobiological model of human affective dynamics. Future work should estimate its parameters from empirical affective time series and evaluate its performance using held-out or out-of-sample observations. Nevertheless, the framework opens promising avenues for applications in psychology, affective computing, psychotherapy analytics, and socially intelligent systems.

## 1 Introduction

Emotional contagion is a psychobiological phenomenon through which affective states spread between individuals via neural mechanisms and cognitive processes [1,2]. It has been described as a fundamental mechanism in the evolution of social cognition, facilitating group cohesion and interpersonal regulation [1,3–5]. This process enables individuals to perceive and replicate others’ emotions through nonverbal cues such as facial expressions, body posture, and tone of voice [6,7]. From a neuroscientific standpoint, emotional contagion involves a complex interaction between sensory, motor, and emotional systems [8–11]. The study of this phenomenon has greatly benefited from neuroimaging techniques, which have helped identify the neural circuits underlying emotional contagion and their connections to empathy, social cognition, and emotional regulation.

Emotional contagion operates through both automatic and controlled pathways [12,13]. In automatic processes, involuntary mimicry of facial expressions and postures facilitates the activation of shared emotional circuits [12,13]. Neuroimaging studies have shown that observing emotional expressions activates the premotor cortex and the insula, suggesting a process of internal simulation [14–16]. However, beyond automatic imitation, emotional contagion can also be modulated by cognitive processes such as emotional reappraisal and response inhibition [17–19]. The dorsolateral prefrontal cortex plays a key role in regulating affective responses, allowing individuals to distinguish between self and other-generated emotions [20,21].

Susceptibility to emotional contagion varies according to individual differences, social context, and one’s baseline affective state. Traits such as empathy, emotional intelligence, and past emotional experiences influence the intensity of emotional contagion [22,23]. Research has shown that activation in the insula and anterior cingulate cortex correlates with a higher propensity to perceive and mirror others’ emotions [11,20,24]. Additionally, social norms and interpersonal closeness impact emotional transmission [25–27]. Cross-cultural studies have found that emotional contagion is more pronounced in collectivist societies than in individualist ones [25,27]. Furthermore, an individual’s baseline emotional state can either enhance or inhibit contagion, as anxiety or stress have been linked to heightened sensitivity to others’ negative emotions.

From a neuroscience perspective, several brain regions have been identified as central to emotional contagion [9,24,28]. The insula plays a critical role in interoceptive awareness and the integration of emotional signals [9,24,28], while the anterior cingulate cortex contributes to affect regulation and emotional synchrony [16,29]. The mirror neuron system, located in the premotor cortex and parietal lobe, supports imitation and affective simulation [11,30,31], and is essential for the automatic understanding of others’ emotional states [21]. The dorsolateral prefrontal cortex and the supramarginal gyrus modulate the intensity of emotional contagion, helping to differentiate self-generated from other-generated emotions [20,21].

The study of emotional contagion has broad applications across multiple disciplines. In clinical psychology, anxiety and depressive disorders are associated with heightened susceptibility to negative emotional contagion, potentially exacerbating clinical symptoms [22,32]. In education, teachers’ emotional states can significantly influence students’ motivation and academic performance [23,33]. In the fields of social neuroscience and communication, emotional contagion plays a key role in marketing and leadership strategies, shaping product perception and collective decision-making [4,5,34].

The present work builds on two mature but only partially overlapping literatures. The first is the literature on dimensional and dynamical models of affect. Russell’s circumplex model provides a continuous valence–arousal coordinate system in which affective states can be represented as positions and trajectories rather than only as discrete categories [35,36]. This dimensional view has motivated several mathematical formulations of emotion dynamics. For example, affective transitions have been represented using piecewise-linear state-space models [37], fractional-order dynamics [38], prediction-error formulations [39], and mathematical models of subjective affect structure [40]. These approaches show that affect can be formalised as a dynamical state whose evolution depends on previous affective configuration, stimulus history, memory, appraisal, and regulation.

A second relevant literature concerns emotional contagion in agent-based and crowd-simulation models. This area has developed explicit mechanisms for interpersonal affective propagation, including contagion rules, dyadic influence, group-level propagation, and emotion-dependent crowd behaviour [41]. Influential agent-based formulations have integrated emotional contagion with beliefs, intentions, and collective decision-making [42], and have modelled emotion contagion in groups through explicit agent-level mechanisms [43]. In crowd and evacuation modelling, related approaches have represented panic, fear, susceptibility, peer influence, and emotion-dependent movement decisions using cellular automata, social-force models, decision rules, and reinforcement-learning frameworks.

The closest relatives to the present formulation are models that move emotional contagion toward a dimensional or mechanically interpretable state space. For example, van Haeringen et al. integrated valence and arousal into an agent-based model of emotion contagion [44], while Rincon et al. proposed a dynamic emotional model for agent societies with attraction-like relations between agents [45]. These works are important because they approach the same modelling space addressed here. However, they also help clarify the specific contribution of the present model: a formulation in which each agent is represented as a continuous affective trajectory in the circumplex plane, while interpersonal influence is expressed through mechanically interpretable radial and angular coupling terms.

Accordingly, the contribution of the present study is not the introduction of emotional contagion modelling itself. Rather, the contribution lies in synthesising a continuous circumplex representation, a phasor-based radial–angular decomposition, multi-agent coupling matrices, and oscillator-like dynamics whose parameters can be interpreted in terms of affective intensity, affective direction, damping, baseline regulation, external forcing, and interpersonal susceptibility.

Building on this positioning, the objective of this article is to present a computationally tractable dynamical-systems model of emotional contagion grounded in the circumplex representation of affect. The model represents each agent’s affective state as a time-dependent phasor in the complex plane, where the radial coordinate represents affective intensity and the angular coordinate represents affective direction in the valence–arousal plane. Inter-individual influence is introduced through coupling matrices acting on both radial and angular dynamics. This structure allows the model to simulate how affective trajectories may converge, diverge, exhibit oscillatory amplification, or synchronize under different assumptions about damping, baseline regulation, external forcing, and social connectivity.

The approach provides a generative simulation framework for exploring emotional coherence, divergence, oscillatory amplification, leader-driven influence, emotional buffering, and collective affective convergence within interconnected populations. The model may prove particularly valuable for psychologists, cognitive scientists, computational neuroscientists, and engineers working in affective computing, as well as practitioners in clinical and social intervention settings who require tools for understanding group-level emotional processes or designing emotion-aware systems. The mathematical expressiveness and computational flexibility of the framework also make it adaptable to a variety of empirical settings, including psychotherapy dynamics, team-based performance, or the development of emotionally responsive artificial agents.

In this article, Section 2 presents the mathematical formulation of the model, including the polar–Cartesian mapping, radial and angular dynamics, coupling-matrix conventions, and numerical implementation. Section 3 defines the test-case scenarios, Section 4 presents and discusses the numerical results and model limitations, and Section 4.2 provides the numerical-convergence and cross-solver analysis.

## 2 Mathematical and computational model

To model the complexity of emotional contagion, we extend Russell’s Circumplex Model of Affect into a dynamical system embedded in the complex plane, where time serves as a third dimension. Emotions are not conceived as static points, but rather as time-dependent trajectories. Each individual’s emotional state is expressed as a phasor, a complex-valued function of time $z(t)=r(t)e^{i\theta (t)}$, where *r*(*t*) represents emotional intensi*t*y (the radial distance from neutrality) and θ(t) encodes the emotional orientation within the circumplex plane, corresponding to the configuration of valence and arousal. This formalism permits a continuous and dynamic representation of affective states as they evolve internally and under the influence of social interactions (see Fig 2).

### 2.1 Mapping between polar variables and valence–arousal coordinates

We represent affective states in the circumplex plane using two normalized Cartesian coordinates: valence $V_{i}(t)$, corresponding to the horizontal pleasant–unpleasant axis, and arousal $A_{i}(t)$, corresponding to the vertical activation–deactivation axis. Because the dynamical formulation below uses $v_{i}(t)={\dot{r}}_{i}(t)$ to denote radial velocity, we use the uppercase symbol $V_{i}(t)$ for valence in order to avoid notational ambiguity.

The affective state of agent *i* can be written equivalently in Cartesian and polar form as


$$\begin{array}{c} z_{i}(t)=V_{i}(t)+iA_{i}(t)=r_{i}(t)\exp\;\left(i\theta_{i}(t)\right), \end{array}$$ (1)

where i is the imaginary unit. The explicit mapping between the polar variables $\left(r_{i},\theta_{i}\right)$ and the valence–arousal coordinates $\left(V_{i},A_{i}\right)$ is


$$\begin{array}{c} V_{i}(t)=r_{i}(t)\cos\theta_{i}(t), \end{array}$$ (2)

$$\begin{array}{c} A_{i}(t)=r_{i}(t)\sin\theta_{i}(t). \end{array}$$ (3)

Conversely, for non-neutral states,


$$\begin{array}{c} r_{i}(t)=\sqrt{V_{i}^{2}(t)+A_{i}^{2}(t)}, \end{array}$$ (4)

$$\begin{array}{c} \theta_{i}(t)=atan2\;\left(A_{i}(t),V_{i}(t)\right). \end{array}$$ (5)

The emotional labels, initial conditions, and attractor coordinates used in the present study are normalized with respect to the unit-disk reference scale,


$$\begin{array}{c} 𝒟=\left\{(V,A)\in {\mathbb{R}}^{2}:V^{2}+A^{2}\leq 1\right\}. \end{array}$$ (6)

Accordingly, the tabulated reference states satisfy $r_{i}\in [0,1]$. In the direct radial baseline, however, $r_{i}(t)$ is integrated as an unconstrained oscillator coordinate and is not upper-bounded by the governing equation. Nonnegative excursions with $r_{i}(t)>1$ are therefore interpreted as model amplitudes beyond the normalized reference-label scale and are not assigned a discrete circumplex label. Negative values remain inadmissible because they are incompatible with the interpretation of $r_{i}(t)$ as radial distance. The alternative constrained formulation enforces $r_{i}(t)\in (\varepsilon_{r},1-\varepsilon_{r})$ whenever strict preservation of the normalized unit disk is required. Under this convention, $r_{i}(t)$ is not itself arousal; rather, it is the affective intensity, defined as the Euclidean distance from the origin of the circumplex. Arousal is the signed vertical coordinate $A_{i}(t)$. Thus, $A_{i}(t)>0$ denotes activation above the reference level, whereas $A_{i}(t)<0$ denotes lower activation or deactivation relative to that reference. Similarly, $V_{i}(t)>0$ and $V_{i}(t)<0$ denote pleasant and unpleasant valence, respectively.

Neutrality is defined as the origin of the normalized circumplex:


$$\begin{array}{c} r_{i}(t)=0\;⟺\;\left(V_{i}(t),A_{i}(t)\right)=(0,0). \end{array}$$ (7)

In measurement terms, this point corresponds to the centred reference value of the affective scale after normalization. It therefore represents absence of net valence and net activation relative to the selected psychometric reference point, not absence of biological emotion. Since the angle is mathematically undefined at the origin, $\theta_{i}(t)$ has no affective interpretation when $r_{i}(t)=0$. In numerical visualizations, any assigned value of $\theta_{i}(t)$ at exact neutrality is only a plotting convention. For empirical applications, near-neutral affective states can be represented using a tolerance neighbourhood


$$\begin{array}{c} {𝒩}_{\varepsilon }=\left\{(V,A)\in 𝒟:\sqrt{V^{2}+A^{2}}<\varepsilon \right\}, \end{array}$$ (8)

where ε>0 should be selected according to the resolution of the measurement instrument or by a predefined sensitivity analysis. In this manner, all points shown in the circumplex plane are plotted using the Cartesian coordinates


$$\begin{array}{c} x=V=r\cos\theta ,\;y=A=r\sin\theta , \end{array}$$

where *V* is valence and *A* is arousal. The radius *r* therefore quantifies affective intensity, while θ defines affective direction.

**Fig 1:**
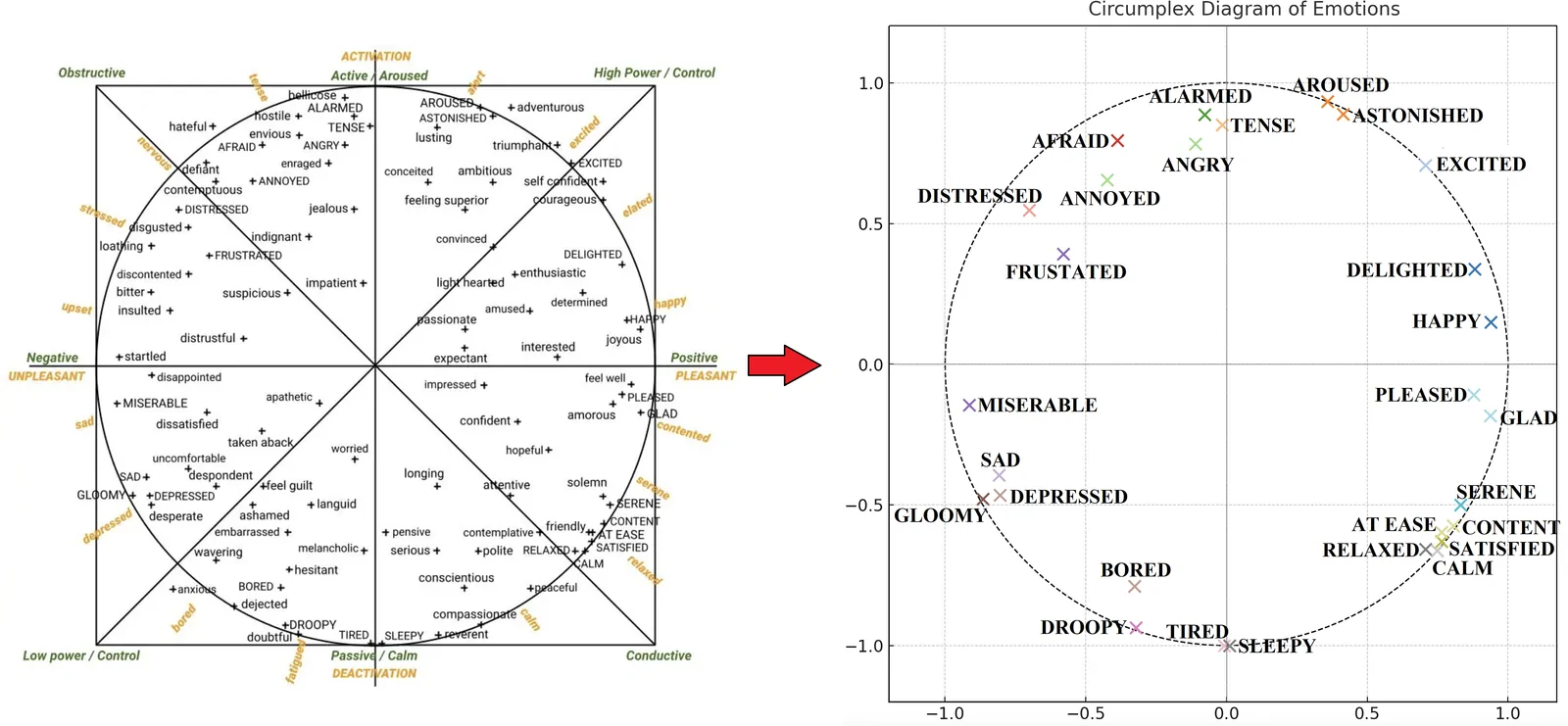
Illustrative construction of the emotion-label coordinates used in Table 1. On the left, a normalized valence–arousal schematic based on Russell’s circumplex organization is shown. On the right, representative emotion-label locations selected in this work are expressed in Cartesian coordinates and mapped to polar form. The radial coordinate *r* represents distance from the neutral origin, whereas the angular coordinate θ is measured from the positive-valence axis. These illustrative coordinates are used to specify initial states and attractor states in the simulations; they are not dataset-derived normative estimates of the emotion terms.

The numerical values reported in Table 1 are illustrative reference coordinates rather than normative estimates obtained from a psychometric dataset. In the present work, representative locations were selected for the emotion labels in the normalized valence–arousal schematic shown in Fig 1, following the general organization of Russell’s circumplex model [35]. With neutrality at (*V*,*A*) = (0,0) and the positive-valence axis defining θ=0, the selected Cartesian locations were converted to polar form using


$$\begin{array}{c} r=\sqrt{V^{2}+A^{2}},\;\theta =atan2(A,V). \end{array}$$

**Table 1:** Illustrative polar reference coordinates for emotion labels used to specify initial and attractor states in the simulations. The values were selected from the normalized schematic shown in Fig 1 and are not dataset-derived psychometric or normative estimates.

EMOTIONAL STATE	Radius	Angle (deg)	Angle (rad)
HAPPY	0.95	9	0.15708
DELIGHTED	0.945	21	0.36652
EXCITED	1.00	45	0.78540
ASTONISHED	0.98	65	1.13446
AROUSED	1.00	69	1.20428
TENSE	0.85	91	1.58825
ALARMED	0.89	95	1.65806
ANGRY	0.79	98	1.71042
ANNOYED	0.78	123	2.14675
AFRAID	0.885	116	2.02458
DISTRESSED	0.89	142	2.47837
FRUSTRATED	0.70	146	2.54818
MISERABLE	0.925	−171	−2.98451
SAD	0.90	−154	−2.68781
GLOOMY	0.99	−151	−2.63545
DEPRESSED	0.93	−150	−2.61799
BORED	0.855	−112.5	−1.96350
DROOPY	0.99	−109	−1.90241
TIRED	1.00	−90.5	−1.57952
SLEEPY	1.00	−89.4	−1.56032
RELAXED	0.965	−43	−0.75049
CALM	1.00	−41.5	−0.72431
SATISFIED	0.99	−39.5	−0.68941
AT EASE	0.97	−38	−0.66323
CONTENT	0.99	−35.5	−0.61959
SERENE	0.97	−31	−0.54105
GLAD	0.955	−11	−0.19199
PLEASED	0.885	−7	−0.12217

The radial distances were normalized with respect to the unit reference scale. The resulting values are used to specify illustrative initial states and attractor states in the numerical examples; they are not intended as unique or empirically validated placements of the corresponding emotion terms. The reported decimal precision facilitates reproducible numerical initialization and should not be interpreted as psychometric precision.

### 2.2 Dynamical model

#### 2.2.1 Radial dynamics.

The evolution of emotional intensity *r*(*t*) is governed by a second-order linear differential equation analogous to a damped driven harmonic oscillator. To facilitate numerical integration and theoretical analysis, this equation can be decomposed into a system of two coupled first-order ordinary differential equations:


$$\begin{array}{c} {\dot{r}}_{i}(t)=v_{i}(t), \end{array}$$ (9)

$$\begin{array}{c} {\dot{v}}_{i}(t)=-b_{r}v_{i}(t)-(r_{i}(t)-r_{i}^{*})+\sum_{j\text{≠}i}K_{ij}^{r}(r_{j}(t)-r_{i}(t))+F_{i}(t), \end{array}$$ (10)

where $r_{i}(t)$ is the affective intensity of agent *i*, defined as distance from the neutral origin of the circumplex, $v_{i}(t)$ is its temporal rate of change, $b_{r}$ is the damping coefficient, $r_{i}^{*}$ is the agent’s baseline affective intensity, and $F_{i}(t)$ is the external forcing. The term $\sum_{j\text{≠}i}K_{ij}^{r}(r_{j}-r_{i})$ reflects emotional contagion: each $K_{ij}^{r}$ quantifies the degree to which agent *j* influences the intensity of agent *i*’s emotional state. This captures scenarios in which affective intensity spreads from one individual to others based on social proximity or psychological susceptibility.

#### 2.2.2 Angular dynamics.

Similarly, the angular dynamics are governed by a nonlinear pendulum-like equation, which we likewise decompose into first-order form:


$$\begin{array}{c} {\dot{\theta }}_{i}(t)=\omega_{i}(t), \end{array}$$ (11)

$$\begin{array}{c} {\dot{\omega }}_{i}(t)=-b_{\theta }\omega_{i}(t)-\sin(\theta_{i}(t)-\theta_{i}^{*})+\sum_{j\text{≠}i}K_{ij}^{\theta }\sin(\theta_{j}(t)-\theta_{i}(t))+\tau_{i}(t), \end{array}$$ (12)

where $\theta_{i}(t)$ represents the valence-arousal angle of agent *i*, $\omega_{i}(t)$ is its angular velocity, $b_{\theta }$ is the angular damping factor, $\theta_{i}^{*}$ is the preferred emotional orientation, and $\tau_{i}(t)$ is a directional external influence. The coupling term $\sum_{j\text{≠}i}K_{ij}^{\theta }\sin(\theta_{j}-\theta_{i})$ encodes directional affective contagion: it models the tendency of agent *i* to align its emotional trajectory with those of its neighbors. In psychological terms, this captures how shared emotional orientations, such as collective joy, grief, or outrage, may emerge from repeated interpersonal interactions.

#### 2.2.3 Mechanical interpretation.

Equations (9)–(12) are written in nondimensional form, with the mechanical mass and angular moment-of-inertia coefficients scaled to unity. In the radial channel, the term $-\left(r_{i}-r_{i}^{*}\right)$ is the elastic restoring contribution: it represents endogenous regulation toward the agent’s preferred affective-intensity baseline $r_{i}^{*}$. The term $-b_{r}v_{i}$ is dissipative and suppresses persistent radial oscillations. Similarly, $-\sin(\theta_{i}-\theta_{i}^{*})$ is the periodic angular analogue of a restoring torque toward the preferred affective direction $\theta_{i}^{*}$, while $-b_{\theta }\omega_{i}$ damps angular motion. Inertia and elasticity are distinct mechanical concepts: inertia arises from the second-order dynamics, whereas the restoring terms pull the state toward equilibrium. Because the inertial coefficients are normalized to one in the present formulation, inertia is not varied as an independent model parameter. References to affective inertia therefore denote qualitative persistence or resistance to rapid change, rather than a separately estimated mass parameter.

#### 2.2.4 External affective inputs.

The functions $F_{i}(t)$ and $\tau_{i}(t)$ represent exogenous influences that do not originate from the agent’s internal baseline or from interpersonal coupling. The radial input $F_{i}(t)$ changes affective intensity, whereas the angular input $\tau_{i}(t)$ acts as an external torque that biases affective direction in the valence–arousal plane. In all simulations reported in this study, these inputs were set to


$$\begin{array}{c} F_{i}(t)=0,\;\tau_{i}(t)=0, \end{array}$$

so that the effects of baseline regulation, damping, and network coupling could be examined in isolation. In future applications, a short pulse could represent an acute event such as sudden distressing news, a step-like input could represent sustained environmental pressure, and a periodic input could represent recurrent exposure such as repeated media or campaign messaging. An event affecting both intensity and direction could be represented through simultaneous nonzero values of $F_{i}(t)$ and $\tau_{i}(t)$.

#### 2.2.5 Constrained radial formulation.

For the direct radial oscillator in Eqs. (9)–(10), the radial coordinate is integrated as an unconstrained state variable. Therefore, although $r_{i}(t)$ is interpreted as affective intensity and is admissible only for $r_{i}(t)\geq 0$, the direct oscillator does not mathematically guarantee non-negativity under all combinations of damping, forcing, coupling strength, and initial velocity. In low-damping or strongly excited regimes, the unconstrained formulation may generate dynamical or numerical overshoots with $r_{i}(t)<0$. Such values have no psychological interpretation as “negative intensity” and are not valid circumplex states. They are instead interpreted as loss of admissibility of the direct radial formulation in that parameter regime.

To preserve the original oscillator structure while making this limitation explicit, we retain the direct radial oscillator as the baseline model used in the numerical examples. We also introduce a constrained radial formulation as an admissibility-preserving alternative for applications in which $r_{i}(t)\in [0,1]$ must be guaranteed by construction. Because the radial coordinate $r_{i}(t)$ represents affective intensity, defined as distance from the neutral origin of the circumplex, the admissible radial domain is


$$\begin{array}{c} 0\leq r_{i}(t)\leq 1. \end{array}$$ (13)

A direct linear oscillator written in the coordinate $r_{i}(t)$ does not, by itself, guarantee that this interval is positively invariant under arbitrary forcing, coupling, or initial radial velocity. To preserve the circumplex interpretation, we therefore formulate the radial dynamics through an unconstrained latent variable


$$\begin{array}{c} \rho_{i}(t)\in \mathbb{R}, \end{array}$$

and define the observable radius through the bounded transformation


$$\begin{array}{c} r_{i}(t)=\sigma_{\varepsilon_{r}}\;\left(\rho_{i}(t)\right)=\varepsilon_{r}+(1-2\varepsilon_{r})\frac{1}{1+\exp[-\rho_{i}(t)]},\;0<\varepsilon_{r}\ll 1. \end{array}$$ (14)

This mapping guarantees


$$\begin{array}{c} r_{i}(t)\in (\varepsilon_{r},1-\varepsilon_{r})\subset [0,1] \end{array}$$ (15)

for all finite values of $\rho_{i}(t)$. Hence, the observable affective state remains inside the normalized unit disk of the circumplex:


$$\begin{array}{c} V_{i}^{2}(t)+A_{i}^{2}(t)=r_{i}^{2}(t)\leq 1. \end{array}$$ (16)

The ideal endpoints *r* = 0 and *r* = 1 are retained as scale endpoints of the normalized circumplex. In simulations using the constrained formulation, however, exact endpoint values are represented using the small tolerance $\varepsilon_{r}$. Thus, values tabulated as *r* = 1 are implemented as $1-\varepsilon_{r}$, and values corresponding to exact neutrality are implemented as $\varepsilon_{r}$ when an angular coordinate is required. This avoids the singularity of the polar angle at exact neutrality while preserving the practical neutral neighbourhood defined in Eq. (8).

For compactness, we define the bounded projection of any prescribed radial attractor as


$$\begin{array}{c} {\bar{r}}_{i}^{\;*}=\Pi_{\varepsilon_{r}}(r_{i}^{*})=\min\left\{1-\varepsilon_{r},\max\left(\varepsilon_{r},r_{i}^{*}\right)\right\}. \end{array}$$ (17)

The radial subsystem is then written in terms of the latent radial coordinate $\rho_{i}(t)$ and its latent velocity $u_{i}(t)={\dot{\rho }}_{i}(t)$:


$$\begin{array}{c} {\dot{\rho }}_{i}(t)=u_{i}(t), \end{array}$$ (18)

$$\begin{array}{c} {\dot{u}}_{i}(t)=-b_{r}u_{i}(t)-\left[r_{i}(t)-{\bar{r}}_{i}^{\;*}\right]+\sum_{j\text{≠}i}K_{ij}^{r}\left[r_{j}(t)-r_{i}(t)\right]+F_{i}(t), \end{array}$$ (19)

where $r_{i}(t)=\sigma_{\varepsilon_{r}}(\rho_{i}(t))$. The term $b_{r}$ is again the radial damping coefficient, $F_{i}(t)$ an external radial forcing term, and $K_{ij}^{r}$ quantifies the influence of agent *j* on the affective intensity of agent *i*. The coupling is evaluated in the observable radius $r_{i}$, so the psychological interpretation of radial contagion is preserved: agents tend to adjust their affective intensity toward that of their neighbours.

The observable radial rate is obtained from the chain rule:


$$\begin{array}{c} {\dot{r}}_{i}(t)=\sigma_{\varepsilon_{r}}^{\prime }\;\left(\rho_{i}(t)\right)u_{i}(t)=\frac{\left[r_{i}(t)-\varepsilon_{r}\right]\left[1-\varepsilon_{r}-r_{i}(t)\right]}{1-2\varepsilon_{r}}u_{i}(t). \end{array}$$ (20)

Thus, as $r_{i}(t)$ approaches either boundary of the admissible interval, the mapping acts as a smooth barrier and prevents the observable radius from crossing outside the circumplex domain.

The angular dynamics are retained as a pendulum-like alignment process:


$$\begin{array}{c} {\dot{\theta }}_{i}(t)=\omega_{i}(t), \end{array}$$ (21)

$$\begin{array}{c} {\dot{\omega }}_{i}(t)=-b_{\theta }\omega_{i}(t)-\sin\left(\theta_{i}(t)-\theta_{i}^{*}\right)+\sum_{j\text{≠}i}K_{ij}^{\theta }\sin\left(\theta_{j}(t)-\theta_{i}(t)\right)+\tau_{i}(t), \end{array}$$ (22)

where $\theta_{i}(t)$ is the affective direction in the valence–arousal plane, $\omega_{i}(t)$ is the angular velocity, $b_{\theta }$ is the angular damping coefficient, $\theta_{i}^{*}$ is the preferred angular attractor, and $\tau_{i}(t)$ is an external directional influence.

The full affective state is reconstructed as


$$\begin{array}{c} z_{i}(t)=r_{i}(t)\exp\;\left(i\theta_{i}(t)\right), \end{array}$$ (23)

with


$$\begin{array}{c} V_{i}(t)=r_{i}(t)\cos\theta_{i}(t),\;A_{i}(t)=r_{i}(t)\sin\theta_{i}(t). \end{array}$$ (24)

Because $r_{i}(t)$ is bounded by construction in this constrained formulation, negative affective intensities cannot occur when the constrained radial model is used.

#### 2.2.6 Separability assumption and observable valence–arousal coupling.

Equations (9)–(12) and Eqs. (18)–(22) define separable polar models for the unconstrained and constrained formulations, respectively. In both formulations, radial dynamics govern changes in affective intensity, whereas angular dynamics govern changes in affective direction. This separation is a modelling assumption introduced to preserve interpretability, reduce the number of free parameters, and isolate the effects of network-mediated contagion on intensity and direction. It should not be interpreted as a claim that intensity and direction are always independent in biological or psychological emotion dynamics.

Although the polar vector field is separable in the baseline formulation, the reconstructed valence–arousal trajectory is not independent in Cartesian coordinates. From


$$\begin{array}{c} V_{i}(t)=r_{i}(t)\cos\theta_{i}(t),\;A_{i}(t)=r_{i}(t)\sin\theta_{i}(t), \end{array}$$ (25)

we obtain


$$\begin{array}{c} {\dot{V}}_{i}(t)={\dot{r}}_{i}(t)\cos\theta_{i}(t)-r_{i}(t){\dot{\theta }}_{i}(t)\sin\theta_{i}(t), \end{array}$$ (26)

$$\begin{array}{c} {\dot{A}}_{i}(t)={\dot{r}}_{i}(t)\sin\theta_{i}(t)+r_{i}(t){\dot{\theta }}_{i}(t)\cos\theta_{i}(t). \end{array}$$ (27)

Therefore, radial and angular motion jointly determine the observable affective trajectory in the circumplex plane. For example, angular motion produces larger Cartesian displacement when the affective intensity $r_{i}(t)$ is high, while the effect of radial motion depends on the current affective direction $\theta_{i}(t)$. In this sense, the baseline model is dynamically separable in polar coordinates but geometrically coupled in the observable valence–arousal representation.

The simulations presented and discussed in this work use this separable baseline. More general nonseparable formulations can be obtained by adding radial–angular feedback terms, but these require additional parameters and empirical calibration. Such extensions are described below as future work rather than being used in the numerical examples reported here.

Interpersonal affective influence is encoded through the radial and angular coupling matrices $K^{r}$ and $K^{\theta }$. Each off-diagonal entry defines a direct influence channel from agent *j* to agent *i*, whereas $K_{ij}=0$ denotes the absence of that direct channel. A zero entry does not necessarily imply that either agent is isolated, because influence may still propagate indirectly through other nodes. The matrix structure therefore specifies the topology, directionality, and relative strength of the contagion network.

#### 2.2.7 Coupling-matrix convention and scaling.

For both the radial and angular channels, the entry $K_{ij}$ denotes the influence exerted by agent *j* on agent *i*; hence rows correspond to receiving agents and columns to source agents. In the most general case the matrices are directed, so that $K_{ij}\text{≠}K_{ji}$. Symmetric matrices represent reciprocal influence, whereas asymmetric matrices represent leader–follower relations, hierarchical influence, or unequal susceptibility. In the baseline simulations reported here, coupling coefficients are restricted to be nonnegative,


$$\begin{array}{c} K_{ij}^{r}\geq 0,\;K_{ij}^{\theta }\geq 0,\;i\text{≠}j, \end{array}$$ (28)

so that the coupling terms act as attractive or consensus-like mechanisms. Negative couplings, $K_{ij}<0$, would represent repulsive or antagonistic affective influence, such as polarization or rejection, and are not considered in the present simulations.

Unless otherwise stated, the diagonal entries are set to zero,


$$\begin{array}{c} K_{ii}^{r}=K_{ii}^{\theta }=0, \end{array}$$ (29)

because the coupling matrices represent interpersonal influence only. Self-regulation is already represented through the baseline attractors $r_{i}^{*}$, $\theta_{i}^{*}$, damping terms, and external forcing terms. The summations in the governing equations are therefore taken over j≠i.

The coupling matrices used in the reported simulations are not row-normalized, and no universal upper bound is imposed on their off-diagonal entries. Therefore, the total incoming coupling strength,


$$\begin{array}{c} \kappa_{i}^{r}=\sum_{j\text{≠}i}K_{ij}^{r},\;\kappa_{i}^{\theta }=\sum_{j\text{≠}i}K_{ij}^{\theta }, \end{array}$$

may differ across agents and scenarios. The coefficients are consequently interpreted as raw nondimensional coupling gains relative to the restoring, damping, and time-scale parameters of the model, rather than as probabilities or normalized averaging weights. Their practical range must be assessed jointly with the damping and restoring terms, the relevant dynamical time scales, and the solver tolerances, and should be verified through numerical-stability and sensitivity analyses.

#### 2.2.8 Scaling of coefficients and final considerations.

The coupling coefficients used in the numerical examples are nondimensional model parameters, scaled relative to the intrinsic restoring terms and the selected simulation time scale. They should not be interpreted as empirical measurements of interpersonal influence. In the reported simulations, the matrices are static within each scenario and are selected to instantiate specific illustrative network structures: directed chains, reciprocal three-agent chains, asymmetric leader–follower chains, weakly connected networks, and networks with a dominant influencer. Time-varying couplings $K_{ij}(t)$, for example depending on attention, proximity, interaction frequency, or context, are a natural extension but are not used in the present study.

The overall emotional state of agent *i* at any time *t* is reconstructed from the combined radial and angular components as $z_{i}(t)=r_{i}(t)e^{i\theta_{i}(t)}$. This complex-valued representation enables visualization and analysis in the circumplex space, both statically and dynamically. When projected over time, the trajectories $z_{i}(t)$ form spirals or loops in three-dimensional space (*x*, *y*, *t*), revealing how emotional contagion unfolds across individuals and populations.

From the standpoint of contagion, the interaction terms play critical roles. The differences $\left(r_{j}-r_{i}\right)$ and $\sin(\theta_{j}-\theta_{i})$ represent affective tension between individuals, which drives alignment or divergence depending on the sign and magnitude of $K_{ij}$. If multiple agents share similar baselines and coupling is strong, the system tends to exhibit synchronized dynamics, where emotional coherence or consensus emerges. Conversely, if the coupling is weak or the network is fragmented, the system may display persistent heterogeneity or irregular, non-convergent affective trajectories

The model proposed in this work can be solved numerically to a high level of accuracy, which allows us to explore diverse scenarios of emotional contagion. These include leader-induced tracking, referred to here as emotional dragging, in which followers move progressively closer to the trajectory of a strongly coupled agent; synchronization waves in homogeneous groups; or dissociation in agents with high damping or low coupling. The model also supports experimentation with group interventions or affective regulation strategies by modulating parameters like damping, baseline attractors, or network topology.

**Fig 2:**
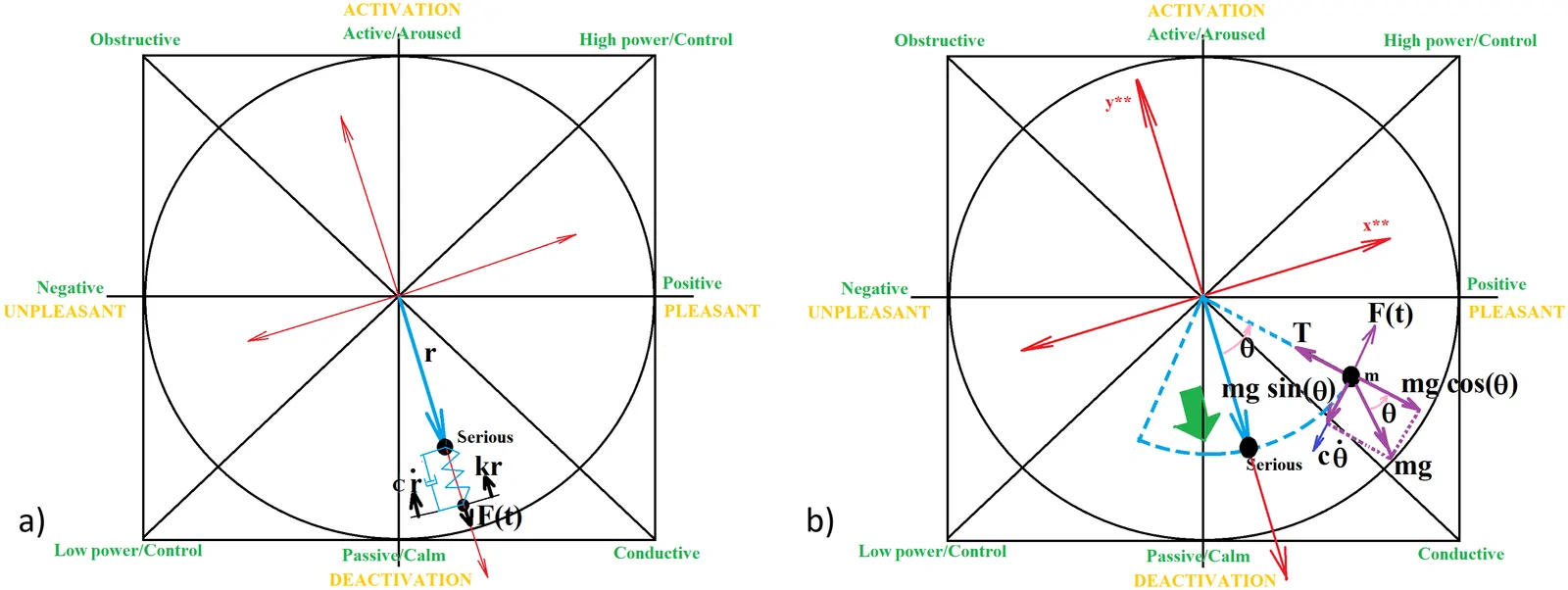
Dual-oscillator representation of affective dynamics within the Circumplex model. (a) Radial oscillator models changes in affective intensity. (b) Angular oscillator models transitions in affective direction within the valence–arousal plane. Emotional contagion is represented through radial and angular interaction matrices.

Fig 2 illustrates the architecture of the dual-oscillator model. Panel (a) shows the radial oscillator governing affective-intensity dynamics, while panel (b) displays the angular oscillator determining affective direction. The radial oscillator models changes in affective intensity, defined as distance from the neutral origin of the circumplex. These two components are jointly embedded within Russell’s circumplex space, forming the core of our simulation platform for emotional contagion.

This mathematical model, grounded in nonlinear dynamical systems and enriched by social coupling mechanisms, provides a generative framework for exploring emotional contagion. By formalizing intra-individual affective regulation and inter-individual affective influence within a common dynamical structure, it offers a computationally tractable way to simulate qualitative patterns of affective alignment, divergence, and co-regulation. Empirical validation and applied use will require future calibration against behavioural, physiological, or interactional data.

### 2.3 Numerical methodology

All numerical simulations were implemented in Python using Jupyter notebooks, NumPy, SciPy, and Matplotlib. The coupled first-order systems were integrated using the scipy.integrate.solve_ivp interface [46]. The reported simulations used method = ’RK45’, which is SciPy’s adaptive explicit Dormand–Prince embedded Runge–Kutta method of orders 5 and 4 [47]. This method is distinct from the classical fixed-step fourth-order Runge–Kutta method.

In the present work a number of simulation scenarios was explored, as presented and discussed in Sec.3. For most reported simulations, the error-control parameters were


$$\begin{array}{c} \text{rtol}=10^{-8},\;\text{atol}=10^{-8}. \end{array}$$ (30)

The three-agent simulations in Examples 4 and 5 used


$$\begin{array}{c} \text{rtol}=10^{-10},\;\text{atol}=10^{-10}. \end{array}$$ (31)

The arguments max_step and first_step were not specified; therefore, the maximum step was not externally bounded and the initial step was selected automatically by the solver.

The solver selected its internal time steps adaptively according to the local error estimate. The arrays supplied through t_eval specified only the times at which the computed solution was stored for plotting and analysis; they did not determine the internal integration steps.

The angular variables $\theta_{i}(t)$ were integrated as continuous, unwrapped real-valued states. No modulo operation was applied inside the differential equations. For plotting and circumplex interpretation, the computed angles were subsequently mapped to the principal interval [−π,π) using


$$\begin{array}{c} \theta_{i}^{wrap}=\left[\left(\theta_{i}+\pi \right)2\pi \right]-\pi . \end{array}$$ (32)

The simulations reported here were not diagnosed as stiff. The main calculations used the adaptive explicit RK45 method. Because strong coupling and rapid transients can increase the computational demands of an explicit solver, numerical robustness was assessed separately through tolerance tightening and comparison with the implicit Radau method, as described in S1 Appendix.

If the constrained radial dynamics version of the model is employed, it is important to perform verification of radial admissibility. In the constrained formulation, the Runge–Kutta scheme advances the latent variables


$$\begin{array}{c} \rho_{i}(t),\;u_{i}(t),\;\theta_{i}(t),\;\omega_{i}(t), \end{array}$$

rather than integrating the observable radius $r_{i}(t)$ as an unconstrained coordinate. After each time step, the radius is reconstructed through Eq. (14). We also record the diagnostic quantities


$$\begin{array}{c} r_{\min}=\min_{i,n}r_{i}(t_{n}),\;r_{\max}=\max_{i,n}r_{i}(t_{n}), \end{array}$$ (33)

where $t_{n}$ denotes the discrete time levels of the simulation. The admissibility condition


$$\begin{array}{c} \varepsilon_{r}\leq r_{\min}\leq r_{\max}\leq 1-\varepsilon_{r} \end{array}$$

is therefore satisfied by construction in simulations that use the constrained radial formulation.

In the implementation used to generate the reported trajectories, the radial state $r_{i}(t)$ was integrated directly. However, the value entering the radial restoring and coupling terms was evaluated through the saturation operator


$$\begin{array}{c} {\hat{r}}_{i}(t)=\Pi_{\left[0,1\right]}\;\left(r_{i}(t)\right)=\min\left\{1,\max\left\{0,r_{i}(t)\right\}\right\}. \end{array}$$ (34)

Thus, ${\hat{r}}_{i}$ rather than the raw state $r_{i}$ was used in the right-hand side of the radial acceleration equation. This numerical saturation does not itself prevent the integrated state from overshooting the interval [0,1].

#### 2.3.1 Quantitative outcome measures.

To operationalize the qualitative terms used in the results, Section 4, we define a set of trajectory-based diagnostics. Let


$$\begin{array}{c} \bar{z}(t)=\frac{1}{N}\sum_{i=1}^{N}z_{i}(t), \end{array}$$ (35)

and define the phase-synchronization order parameter as


$$\begin{array}{c} R_{\theta }(t)=\left|\frac{1}{N}\sum_{i=1}^{N}\exp\;\left(i\theta_{i}(t)\right)\right|,\;0\leq R_{\theta }(t)\leq 1. \end{array}$$ (36)

A value $R_{\theta }=1$ indicates perfect phase alignment, whereas lower values indicate increasing phase dispersion. Because phase alignment does not necessarily imply agreement in affective intensity, we also define the dispersion of the complete circumplex states:


$$\begin{array}{c} D_{z}(t)={\left[\frac{1}{N}\sum_{i=1}^{N}{\left|z_{i}(t)-\bar{z}(t)\right|}^{2}\right]}^{1/2}. \end{array}$$ (37)

Thus, full affective-state synchronization corresponds to high $R_{\theta }(t)$ together with low $D_{z}(t)$; $D_{z}=0$ means that all agents occupy the same valence–arousal state.

For finite-horizon comparisons, we define the initial and terminal windows as


$$\begin{array}{c} {𝒲}_{0}=[0,0.1T],\;{𝒲}_{f}=[0.9T,T], \end{array}$$

and denote the temporal average over a window by


$$\begin{array}{c} \langle f\rangle_{W}=\frac{1}{\left|W\right|}\int_{W}f(t)\;dt. \end{array}$$

The normalized convergence ratio is


$$\begin{array}{c} C_{z}=\frac{{\left\langle D_{z}\right\rangle }_{W_{f}}}{{\left\langle D_{z}\right\rangle }_{W_{0}}+\varepsilon_{m}},\;\varepsilon_{m}>0. \end{array}$$ (38)

Values $C_{z}<1$ indicate contraction of the group-state distribution (convergence), whereas $C_{z}>1$ indicates increased dispersion (divergence).

For scenarios containing a designated leader ℓ, the mean follower–leader distance is


$$\begin{array}{c} D_{\ell }(t)=\frac{1}{N-1}\sum_{i\text{≠}\ell }\left|z_{i}(t)-z_{\ell }(t)\right|, \end{array}$$ (39)

and the corresponding leader-following or “dragging” index is


$$\begin{array}{c} G_{\ell }=1-\frac{{\left\langle D_{\ell }\right\rangle }_{W_{f}}}{{\left\langle D_{\ell }\right\rangle }_{W_{0}}+\varepsilon_{m}}. \end{array}$$ (40)

Positive $G_{\ell }$ denotes net movement toward the leader trajectory, whereas $G_{\ell }<0$ denotes increasing separation.

The present simulations do not include a frequency-response analysis; therefore, we use the term *oscillatory amplification* rather than *resonance*. For agent *i*, define the within-window oscillation amplitude


$$\begin{array}{c} \sigma_{z,i}(W)={\left[{\left\langle {\left|z_{i}(t)-{\left\langle z_{i}\right\rangle }_{W}\right|}^{2}\right\rangle }_{W}\right]}^{1/2}, \end{array}$$ (41)

and the group amplification ratio


$$\begin{array}{c} A_{osc}=\frac{N^{-1}\sum_{i=1}^{N}\sigma_{z,i}(W_{f})}{N^{-1}\sum_{i=1}^{N}\sigma_{z,i}(W_{0})+\varepsilon_{m}}. \end{array}$$ (42)

A value $A_{osc}>1$ indicates growth of oscillatory amplitude over the simulated interval.

No trajectory in the present study is classified as chaotic. Such a classification would require, at minimum, evidence of a positive largest Lyapunov exponent under numerically converged integration and a dedicated initial-condition perturbation analysis, neither of which is performed here.

The simulated trajectories


$$\begin{array}{c} z_{i}(t)=r_{i}(t)e^{i\theta_{i}(t)} \end{array}$$

generated through numerical integration form the basis of the computational analysis. They can be visualised as paths in the three-dimensional space (*V*,*A*,*t*) or projected onto the circumplex plane. This simulation pipeline is used here to examine how specified attractors, damping coefficients, coupling structures, and forcing terms influence qualitative patterns of affective propagation, alignment, and divergence. In fact, with this approach, one could potentially explore in a systematic manner how emotion spreads across networks, how personality traits potentially affect susceptibility to contagion, and how targeted interventions may disrupt or reinforce collective affective states. However, it is important to note that the model in its current development does not explicitly parameterize personality traits or interventions.

## 3 Test case scenarios and examples

This section presents computational scenarios designed to illustrate the qualitative behaviour of the proposed phase-based emotional-oscillator model. The simulations generate model-based affective trajectories, inter-agent alignment, and collective patterns under prescribed structural and dynamical conditions. They are intended as exploratory in-silico examples and are not presented as empirically validated predictions of human emotional behaviour. Nevertheless, these computational scenarios aim to demonstrate the model’s ability to generate emotional trajectories, inter-individual synchronization, and collective affective patterns under different structural and dynamic conditions.

We consider three distinct simulation *scenarios*, each reflecting a particular social or psychological configuration:

1. **Scenario 1 – Minimal triadic interaction:** A simplified system consisting of three emotional oscillators (individuals), each initialized at a specific emotional state. One of them is assigned an emotional attractor, and the emotional interactions among the three are observed as they evolve over time.
2. **Scenario 2 – Dual group leadership dynamics:** Two separate groups of 10 individuals each are modeled. Within each group, one designated leader exerts influence over the rest. This configuration explores intra-group contagion dynamics, mutual alignment, and emotional stabilization within and across groups.
3. **Scenario 3 – Complex network with dominant influencer:** A more realistic complex network of 20 individuals is simulated, in which all nodes are connected and influenced by an external dominant source—such as a social media platform (*e.g., TikTok* or *Facebook*)—represented by the first oscillator. This scenario captures centralized contagion and heterogeneous influence in digital social ecosystems.

Each scenario includes a set of numerical examples designed to explore its behavior under varying conditions. In total, we present **nine numerical examples**, representing different parameterizations of emotional coupling strength, initial emotional states, and assigned attractor targets. These examples will be detailed in the following subsections and are intended to demonstrate the flexibility and robustness of the model across a diverse range of affective contexts.

It is important to note that the contagion matrix 𝐊, as introduced in Equations (9)–(12), could theoretically assign different values for the radial and angular components of emotional dynamics within the extended Russell circumplex model. However, in this study, we simplify the formulation by assuming that the same contagion matrix 𝐊 governs both the evolution of the radius *r* and the angle θ in the complex emotion plane.

Additionally, the damping coefficients applied to both components were also kept identical across all simulations. Specifically, we used the values $b^{r}=b^{\theta }=0.01$, $b^{r}=b^{\theta }=0.1$, and $b^{r}=b^{\theta }=1.0$ as damping constants, equally applied to the radial and angular dynamics. This modeling choice was made to isolate the effects of network topology and contagion strength, ensuring consistency and interpretability of the emotional trajectories generated under varying structural conditions.

### 3.1 Scenario 1: Transitivity

**Fig 3:**
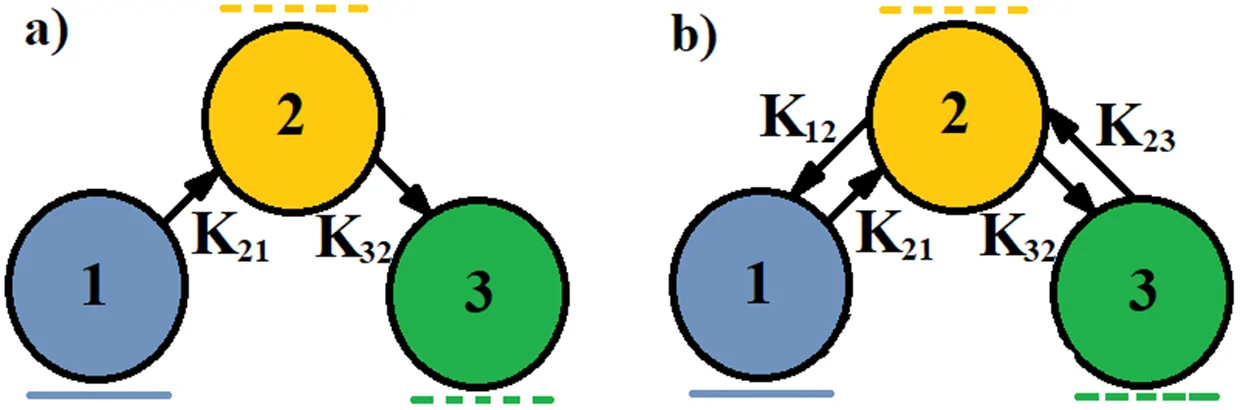
Topologies of emotional contagion in three-agent systems. (**A**) Directed emotional contagion scenario: Oscillator 1 (source, initial state “HAPPY”) influences oscillator 2 (intermediary, “BORED”), who in turn influences oscillator 3 (observer, “DISTRESSED”). No direct connection exists between oscillator 1 and 3. Arrows represent the directional couplings encoded in the contagion matrix 𝐊, capturing a transitive emotional influence structure. (**B**) Reciprocal three-agent chain: oscillator 2 is bidirectionally coupled to oscillators 1 and 3, whereas oscillators 1 and 3 have no direct connection. The links 1↔2 and 2↔3 have equal coupling strength. This topology permits reciprocal influence along the existing dyadic links while retaining oscillator 2 as the central intermediary. This configuration supports stronger emotional convergence and captures group-level emotional synchronization processes, as may occur in tightly bonded families, close peer groups, or therapeutic triads. The color and line type displayed above and below each figure serve as identifiers and are used consistently in the results to facilitate interpretation and comparison.

In the first simulation scenario explored in this work, we examine the dynamics of emotional contagion through a transitive pathway involving three agents (oscillators), each initialized in distinct affective states within the valence-arousal space. Oscillator 1 serves as the emotional source, beginning in the “HAPPY” quadrant and governed by an intrinsic attractor state corresponding to “ANGRY”—a transition that models internal emotional drift. Oscillator 2, the intermediary, begins in a “BORED” state, while oscillator 3, the final observer, starts in a “DISTRESSED” emotional position (see Fig 3a)). Importantly, oscillator 3 cannot perceive oscillator 1 directly; instead, emotional influence must propagate transitively from 1 to 2, and from 2 to 3. Initial emotional conditions are given numerically by (see Table 1 and Fig 1):


$$\begin{array}{c} r_{0}=[\begin{array}{c} 0.95\\ 0.855\\ 0.89 \end{array}],\;v_{0}=[\begin{array}{c} 0\\ 0\\ 0 \end{array}],\;\theta_{0}=[\begin{array}{c} 0.15708\\ -1.96350\\ 2.47837 \end{array}],\;\omega_{0}=[\begin{array}{c} 0\\ 0\\ 0 \end{array}] \end{array}$$

and the attractor points are defined by


$$\begin{array}{c} r_{\text{attractor}}=[\begin{array}{c} 0.79\\ 0\\ 0 \end{array}],\;\theta_{\text{attractor}}=[\begin{array}{c} 1.71042\\ 0\\ 0 \end{array}] \end{array}$$

This setup reflects realistic emotional dynamics in indirect social interactions, such as in family or team environments. For example, an emotionally volatile parent (oscillator 1) may indirectly affect the child (oscillator 3) through the emotional mediation of the other parent or caregiver (oscillator 2). Similarly, this chain of influence could describe emotional contagion in hierarchical organizations, where the emotional climate of leadership gradually permeates peripheral members via managerial intermediaries.

To capture this structure, we define a directed contagion matrix 𝐊 in which influence is allowed from oscillator 1–2, and from 2 to 3, but not directly from 1 to 3. The simulation explores how this emotional signal travels through the intermediary under various damping regimes, allowing us to study the strength and fidelity of transitive contagion.The coupling matrices are given by (example 1):


$$\begin{array}{c} 𝐊={𝐊}_{r}={𝐊}_{\theta }=(\begin{array}{ccc} 0 & 0 & 0\\ 50 & 0 & 0\\ 0 & 50 & 0 \end{array}) \end{array}$$

To investigate the directional dynamics of emotional influence in a triadic system, we define a series of coupling matrices 𝐊 that govern how emotional states propagate through the network of oscillators. In the first configuration, all elements of 𝐊 are set to zero except for *K*_21_ and *K*_32_, which are assigned the same value of 50.0. This means that oscillator 1 (the emotional source) directly influences oscillator 2 (the intermediary) with the same strength that oscillator 2 influences oscillator 3 (the observer). Psychologically, this setup simulates a scenario in which emotional influence is transmitted with equal strength along a transitive path — for example, a parent affecting a co-parent, who in turn affects the child. By using equal weights, we assess whether the final emotional impact on oscillator 3 is comparable to that of oscillator 1, even when filtered through a third party.

To explore the role of asymmetry in emotional transmission, we then vary the coupling values. In one example, we set *K*_21_ = 50 and *K*_32_ = 5, yielding the matrix (example 2):


$$\begin{array}{c} 𝐊={𝐊}_{r}={𝐊}_{\theta }=(\begin{array}{ccc} 0 & 0 & 0\\ 50 & 0 & 0\\ 0 & 5 & 0 \end{array}) \end{array}$$

This configuration reflects a situation where the source has a strong emotional impact on the intermediary, but the intermediary weakly transmits that influence to the observer. Psychologically, this could resemble a family system in which one partner internalizes strong emotional input from the other, but acts as an emotional buffer for the child.

In a third example, we reverse the relationship by assigning *K*_21_ = 50 and *K*_32_ = 500 (example 3):


$$\begin{array}{c} 𝐊={𝐊}_{r}={𝐊}_{\theta }=(\begin{array}{ccc} 0 & 0 & 0\\ 50 & 0 & 0\\ 0 & 500 & 0 \end{array}) \end{array}$$

Here, although the source continues to influence the intermediary strongly, the intermediary amplifies and transmits this emotion with much greater force to the observer. This configuration models emotional over-identification or emotional reactivity, where an intermediary figure not only absorbs emotion from the source but projects it even more intensely onto the third party. These different matrix configurations allow us to examine how emotional influence evolves depending on the relative strength of connections between individuals in a psychological system.

In addition, in Fig 3b), we illustrate a configuration involving mutual coupling among the three oscillators. In this scenario, the coupling matrix includes off-diagonal elements indicating bidirectional influence, and is defined as (example 4):


$$\begin{array}{c} 𝐊={𝐊}_{r}={𝐊}_{\theta }=(\begin{array}{ccc} 0 & 50 & 0\\ 50 & 0 & 50\\ 0 & 50 & 0 \end{array}) \end{array}$$

The nonzero pairs $K_{12}=K_{21}=50$ and $K_{23}=K_{32}=50$ represent equal reciprocal influence along the links 1↔2 and 2↔3. Oscillator 2 is the central node, while oscillators 1 and 3 interact only indirectly through oscillator 2. Thus, all existing links are reciprocal and symmetric, but the network is not fully connected. Psychologically, this configuration may represent a group in which a central intermediary supports reciprocal affective exchange between two individuals who are not directly connected.

Additionally, we examine a configuration where emotional influence is asymmetrically stronger in one direction. In this case, the coupling matrix is defined as (example 5):


$$\begin{array}{c} 𝐊={𝐊}_{r}={𝐊}_{\theta }=(\begin{array}{ccc} 0 & 50 & 0\\ 500 & 0 & 50\\ 0 & 500 & 0 \end{array}) \end{array}$$

This matrix represents a hierarchical system in which oscillator 2 is highly sensitive to oscillator 1 and strongly influences oscillator 3, but the reverse influences are much weaker. The psychological interpretation is a top-down emotional dynamic, where a central figure (oscillator 2) acts as a powerful transmitter of the source’s emotional state to a downstream individual. Such dynamics are often observed in environments characterized by emotional dominance or pressure, where a caregiver or leader strongly channels affective cues from an emotionally intense source toward others who are less able to modulate or reflect those emotions back.

### 3.2 Scenario 2: Leader-driven coupling dynamics

In the following set of simulations, we examine the evolution of emotional states within two distinct groups, each composed of 10 individuals (oscillators), under conditions of heterogeneous emotional initialization. This design allows us to investigate how emotional contagion and coupling mechanisms shape the collective emotional dynamics when each individual starts from a distinct position within the Russell circumplex model.

The initial emotional states are defined (see Table 1) in terms of their radial distance (*r*_0_), angular position ($\theta_{0}$), and corresponding velocity parameters (*v*_0_, $\omega_{0}$). These parameters are assigned based on affective labels commonly mapped to specific regions in the valence-arousal plane. Individuals in Group 1 are initialized with high-arousal and positive-valence states such as *“Pleased,” “Glad,” “Serene,” “Content,” “At Ease,” “Satisfied,” “Calm,” “Relaxed,” “Sleepy,”* and *“Tired”*. In contrast, Group 2 includes states typically associated with low or negative valence and variable arousal, such as *“Droopy,” “Bored,” “Depressed,” “Gloomy,” “Sad,” “Miserable,” “Frustrated,” “Distressed,” “Afraid,”* and *“Annoyed”*.

These emotional configurations are mathematically represented as follows:


$$\begin{array}{cc} r_{0} & =[\begin{array}{c} 0.885,0.955,0.97,0.99,0.97,0.99,1.0,0.965,1.0,1.0,\\ 0.99,0.855,0.93,0.99,0.90,0.925,0.70,0.89,0.885,0.78 \end{array}]\\ v_{0} & =[\begin{array}{c} 0,0,0,0,0,0,0,0,0,0,0,0,0,0,0,0,0,0,0,0 \end{array}]\\ \theta_{0} & =[\begin{array}{c} -0.1222,-0.1920,-0.5411,-0.6196,-0.6632,-0.6894,-0.7243,-0.7505,\\ -1.5603,-1.5795,-1.9024,-1.9635,-2.6180,-2.6355,-2.6878,-2.9845,\\ 2.54818,2.4784,2.0246,2.1468 \end{array}]\\ \omega_{0} & =[\begin{array}{c} 0,0,0,0,0,0,0,0,0,0,0,0,0,0,0,0,0,0,0,0 \end{array}] \end{array}$$

These initial values allow for the simulation of realistic emotional landscapes within groups that may represent, for example, different social factions or populations reacting to distinct leadership outcomes. By setting diverse starting points along the circumplex, we can observe how the interaction structure and emotional coupling—defined by the contagion matrix 𝐊—modulate the evolution toward group-level emotional coherence or divergence.

**Fig 4:**
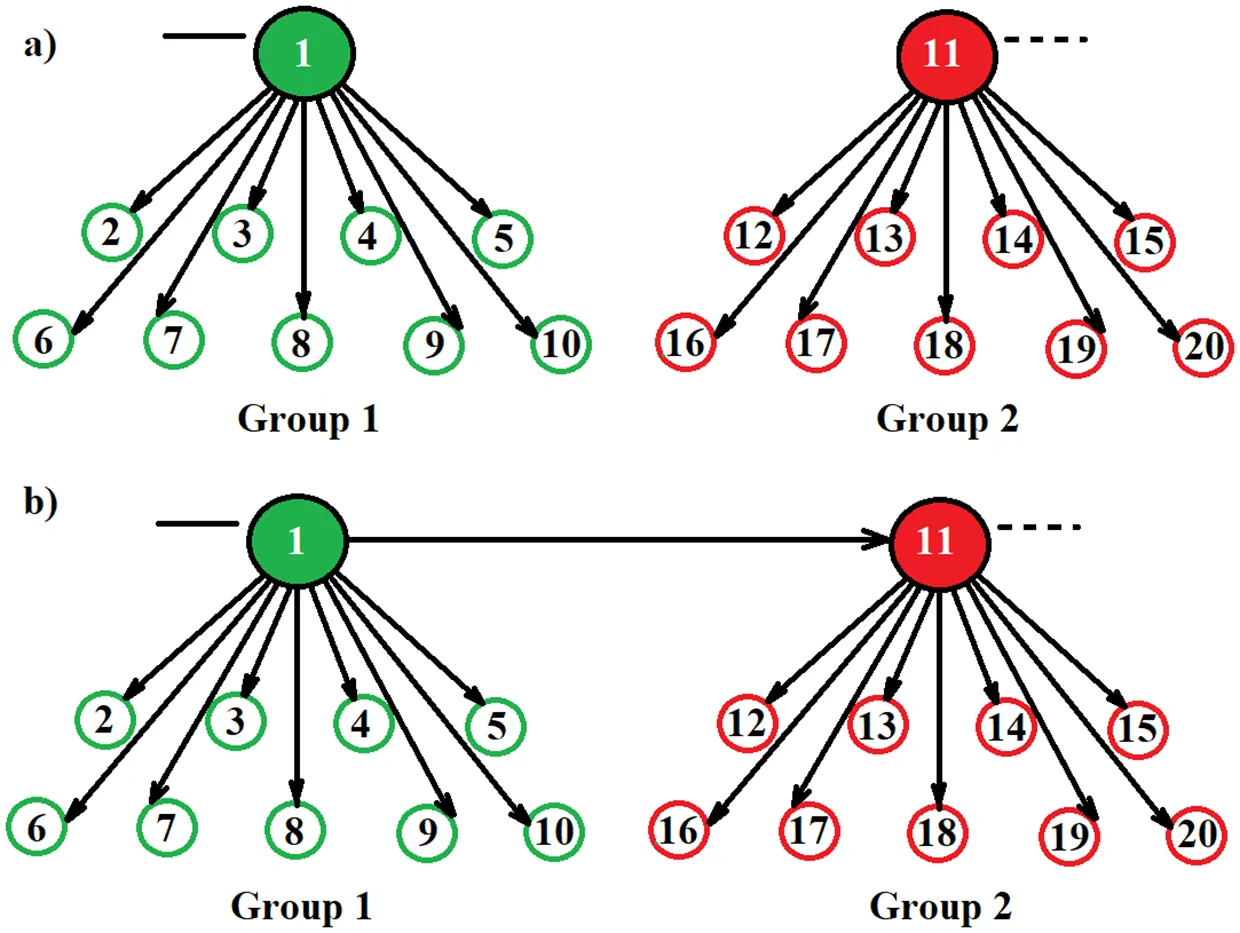
Simulation of emotional dynamics in two distinct groups with leader-driven coupling. Panels (a) and (b) illustrate the temporal evolution of emotional states in two social groups (10 individuals each), initialized with heterogeneous affective states mapped onto the valence-arousal circumplex. In panel (a), each group is influenced unidirectionally by an emotional leader (oscillator 1 for Group 1, oscillator 11 for Group 2), whose emotional attractors are *“Happy”* and *“Frustrated”*, respectively. This configuration mimics group responses to divergent outcomes—such as winning or losing an election—under strong intra-group emotional influence. Panel (b) introduces inter-group coupling, modeling an antagonistic interaction between the leaders. Oscillator 1 now transitions to an *“Angry”* attractor and exerts emotional influence on oscillator 11, initiating cross-group emotional contagion. Both configurations reveal how leader-centered emotional contagion mechanisms can produce emergent collective affective patterns, synchronization, or conflict, depending on the directionality and strength of the coupling.

In the sixth and seventh simulation examples (see Fig 4), we examine the dynamics of emotional contagion within two groups, each composed of 10 individuals. These scenarios model realistic socio-affective processes that occur in everyday life, particularly within communities subject to collective decisions, such as elections or public opinion shifts.

In the first group-coupling example (Fig 4a), we consider two parallel groups, each supporting a different leader or candidate. After a hypothetical election, one leader is assumed to win while the other loses. Each group’s affective response is consequently influenced by the trajectory of its respective leader. In this scenario, the leader attractors are assigned as *Happy* for oscillator 1 (winner) and *Frustrated* for oscillator 11 (loser); their numerical coordinates are given in Table 1. Typically, the winning group may gravitate toward emotional states such as joy or satisfaction, while the losing group may experience frustration, disappointment, or anger. An interesting aspect to study in this kind of scenarios is the extent to which each leader emotionally influences their followers. A highly expressive or emotionally dominant leader can shape the emotional trajectory of their entire group.


$$\begin{array}{cc} r_{\text{atractor}} & =[\begin{array}{c} 0.95,0,0,0,0,0,0,0,0,0,0.70,0,0,0,0,0,0,0,0,0 \end{array}]\\ \theta_{\text{atractor}} & =[\begin{array}{c} 0.15708,0,0,0,0,0,0,0,0,0,2.54818,0,0,0,0,0,0,0,0,0 \end{array}] \end{array}$$

To model this phenomenon, we constructed a coupling matrix 𝐊, which encodes the strength of emotional influence from each leader to the other members of their respective group. For individuals indexed from 1 to 10, emotional influence originates from leader 1; for those indexed from 11 to 20, influence is driven by leader 11. This setup allows us to simulate how emotional states propagate within groups via leader-follower dynamics.

To implement the contagion dynamics within each of the two groups, we define a structured emotional coupling matrix ${𝐊}_{r}={𝐊}_{\theta }$ that governs the influence of group leaders on their respective members. Specifically, each group contains one emotional leader—oscillator 1 for Group 1 and oscillator 11 for Group 2—who acts as the primary source of affective influence. The remaining members in each group are only influenced by their corresponding leader and do not influence each other or the other group.

Formally, the elements of the coupling matrix are defined as (example 6):


$$\begin{array}{c} K_{r}(i,j)=K_{\theta }(i,j)=\left\{ \begin{array}{cc} 50, & \text{if }(j=1\text{ and }i\in \{2,...,10\})\\ 50, & \text{if }(j=11\text{ and }i\in \{12,...,20\})\\ 0, & \text{otherwise} \end{array}\right. \end{array}$$

This configuration captures a leader-follower structure in which emotional contagion flows unidirectionally from each leader to their followers, simulating a psychological scenario in which a dominant emotional agent within each group sets the tone for the rest. Such a setup is commonly observed in political, social, or organizational contexts where group members align emotionally with a central figure, especially following emotionally salient events such as elections, crises, or celebrations. The matrix design enables us to analyze how the emotional attractor states of leaders shape the collective dynamics of each group over time.

This framework captures real-world phenomena such as collective euphoria or mass depressive states, where large segments of a population synchronize emotionally due to shared identification with a dominant emotional figure. Psychologically, such dynamics illustrate how affective leadership and emotional coupling can drive group-level emotional states, potentially leading to large-scale social outcomes—ranging from political mobilization to collective emotional distress.

As a second group-based simulation (see Fig 4b), we consider a scenario in which two emotionally organized groups are each led by a distinct leader—oscillator 1 and oscillator 11, respectively. These two leaders represent rival figures who engage in a form of emotional confrontation, simulating real-world situations such as interpersonal conflict between group representatives at a social event, sports match, or public demonstration.

In this context, emotional influence is not confined within each group but extends across them, specifically through the coupling from oscillator 1 to oscillator 11. This configuration allows us to examine how emotional states from one dominant figure can potentially affect a rival leader and, by extension, the opposing group. Such dynamics are relevant to understanding emotional escalation between groups and the role of inter-leader interactions in spreading heightened affective states, such as anger or agitation, across social boundaries. Once again, the leader of the first group (oscillator 1) transitions to the emotional state *“Angry”* (see Table 1) and confronts the leader of the second group (oscillator 11), as illustrated in Fig 4. This interaction initiates the conflict between the two groups. Consequently, the attractor state vector, where only the first oscillator has a clear attractor, is defined as (Example 7):


$$\begin{array}{cc} r_{\text{atractor}} & =[\begin{array}{c} 0.79,0,0,0,0,0,0,0,0,0,0,0,0,0,0,0,0,0,0,0 \end{array}]\\ \theta_{\text{atractor}} & =[\begin{array}{c} 1.71042,0,0,0,0,0,0,0,0,0,0,0,0,0,0,0,0,0,0,0 \end{array}] \end{array}$$

The simulation is designed to explore the implications of inter-group coupling and the emergence of collective emotional patterns rooted in leader rivalry. This setting highlights the psychological mechanisms by which emotionally salient interactions between influential figures can propagate through their respective networks, fostering either conflict or synchronization depending on the system’s parameters. Therefore, the coupling emotional factors are given by:


$$\begin{array}{c} K_{r}(i,j)=K_{\theta }(i,j)=\left\{ \begin{array}{cc} 50, & \text{if }(j=1\text{ and }i\in \{2,...,11\})\\ 50, & \text{if }(j=11\text{ and }i\in \{12,...,20\})\\ 0.5, & \text{if }(j=11\text{ and }i=1)\\ 0, & \text{otherwise} \end{array}\right. \end{array}$$

### 3.3 Scenario 3: Complex network

For this scenario, we adopt the same initial conditions used in Scenario 2, in which two emotionally coupled groups interact under the leadership of a designated individual within each group. The emotional states of all oscillators at *t* = 0 are thus initialized identically to those used in Scenario 2, ensuring comparability between the dynamics of isolated group leadership and the dynamics within a larger, complex network.

The remainder of the parameters—including the emotional attractor states and the contagion matrix 𝐊—will be introduced progressively throughout the detailed description of this scenario. This setup allows us to observe how the introduction of a dominant external influencer (e.g., a social media entity) alters the emotional dynamics and synchronization patterns of an otherwise balanced and symmetric group structure.

**Fig 5:**
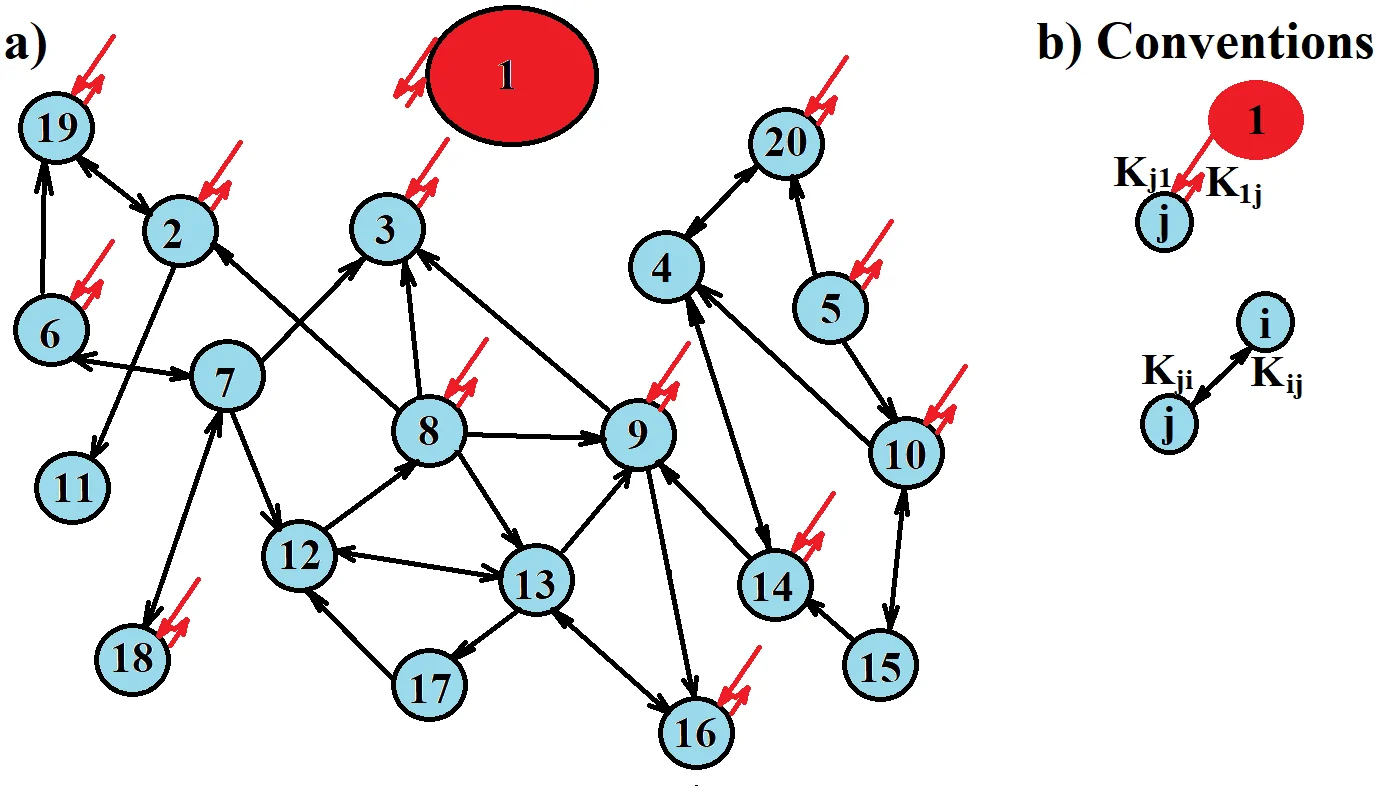
a) Visualization of a social-emotional network where nodes represent individuals and arrows indicate directed emotional influence. Red arrows originate from a central individual (node 1, in red), with arrow length corresponding to the strength of influence. Longer red arrows denote stronger affective impact on target nodes, while shorter ones indicate attenuated influence. Black arrows represent mutual or unidirectional emotional contagion between individuals within the periphery of the network. b) The right side of the figure displays the graphical conventions used to represent contagion and directionality, including how these influences are encoded in the contagion matrix through coefficients $K_{ij}$ and $K_{ji}$, as described in the mathematical formulation. This figure demonstrates the model’s ability to simulate complex, asymmetric emotional propagation patterns within heterogeneous social structures.

In Fig 5, the red arrows—particularly those of greater length—represent the influence exerted by a central individual (highlighted in red) over others within the emotional-social network. For instance, the red node labeled as individual 1 exerts a direct emotional influence on individuals 2, 3, 5, 6, 8, 9, 10, 14, 16, 18, 19, and 20. These longer arrows indicate a higher contagion strength or transmission priority, possibly reflecting charismatic leadership, frequent communication, or centrality within the digital or physical social structure.

In contrast, the shorter red arrows emanating from the red individual indicate that their influence is not uniform across the network and may be attenuated due to contextual or technological barriers. Such barriers could include limited access to digital communication platforms (e.g., mobile apps or web interfaces), or social dynamics in which certain individuals are less susceptible or even resistant to emotional influence from the source node.

The black arrows indicate bidirectional or unidirectional emotional influence between individuals within the network who are not directly affected by the central red node. For example, in the upper-left quadrant of the figure, individual 19 exerts an influence on individual 2 (as shown by the arrow from 19 to 2), and reciprocally, individual 2 influences individual 19 (arrow from 2 to 19). These interactions are encoded in the contagion matrix as coefficients $K_{ij}$ and $K_{ji}$, representing the strength and directionality of the emotional coupling between agents *i* and *j*.

This network does not constitute a fully centralized structure influenced solely by the red individual. Rather, it mirrors the complexity found in real-life social systems, where emotional influence propagates through multifaceted pathways involving familial, affective, and social ties. For instance, individual 8 may be influenced by node 12—potentially a parental figure—while simultaneously influencing individuals 2, 3, 9, and 13, who might represent peers or friends. These diverse influence pathways illustrate the layered and context-sensitive nature of emotional contagion in realistic social networks.

Although this specific configuration represents a simplified instantiation, it is sufficiently expressive for the purpose of demonstrating the model’s capacity to simulate diverse emotional routes within a complex network. The resulting emotional trajectories depend critically on the contagion coefficients and individual parameters previously described, thereby illustrating the range of nonlinear, context-dependent affective trajectories that the model can generate.

In this example, we define a set of emotional attractor states in the valence-arousal space, each characterized by a specific angle θ and radius *r* in polar coordinates, given by:


$$\begin{array}{c} r_{\text{atractor}}=[\begin{array}{cccccccccc} 0.95 & 0.945 & 1.0 & 0.98 & 1.0 & 0.85 & 0.89 & 0.79 & 0.78 & 0.885\\ 0.890 & 0.7 & 0.925 & 0.90 & 0.99 & 0.93 & 0.855 & 0.99 & 1.0 & 1.0 \end{array}] \end{array}$$

$$\begin{array}{c} \theta_{\text{atractor}}=[\begin{array}{cccccccccc} 0.1571 & 0.3665 & 0.7854 & 1.1345 & 1.2043 & 1.5883 & 1.6581 & 1.7104 & 2.1468 & 2.0246\\ 2.4784 & 2.5482 & -2.9845 & -2.6878 & -2.6355 & -2.6180 & -1.9635 & -1.9024 & -1.5795 & -1.5603 \end{array}] \end{array}$$

The attractor states included are: *“happy”*, *“delighted”*, *“excited”*, *“astonished”*, *“aroused”*, *“tense”*, *“alarmed”*, *“angry”*, *“annoyed”*, *“afraid”*, *“distressed”*, *“frustrated”*, *“miserable”*, *“sad”*, *“gloomy”*, *“depressed”*, *“bored”*, *“droopy”*, *“tired”*, and *“sleepy”*.

Each of these states is assigned a polar coordinate based on theoretical positioning within the Russell circumplex model.

Additionally, in this example, higher values of emotional influence have been assigned randomly to the first oscillator (node 1), representing a dominant emotional source—such as a social media platform (e.g., *TikTok* or *Facebook*)—capable of exerting strong affective contagion over the network. These elevated values, all greater than 50, were placed along the first column of the contagion matrix, indicating the unidirectional influence from node 1 to the rest of the network. In contrast, the interactions among the remaining individuals (represented by black arrows in Fig 5) were assigned lower random values, ranging between 20 and 40. These values reflect weaker, decentralized emotional coupling. It is important to note that all entries in the contagion matrix are assigned randomly within their respective ranges, capturing the heterogeneity of influence patterns typically observed in social systems.

The resulting contagion matrix, denoted by 𝐊, is given by (Example 8):


$$\begin{array}{c} 𝐊=\left[\begin{array}{cccccccccccccccccccc} 0 & 43 & 45 & 0 & 43 & 44 & 0 & 47 & 48 & 42 & 0 & 0 & 0 & 47 & 0 & 48 & 0 & 44 & 42 & 47\\ 61 & 0 & 0 & 0 & 0 & 0 & 0 & 0 & 35 & 0 & 0 & 0 & 0 & 0 & 0 & 0 & 0 & 0 & 37 & 0\\ 58 & 0 & 0 & 0 & 0 & 0 & 20 & 34 & 26 & 0 & 0 & 0 & 0 & 0 & 0 & 0 & 0 & 0 & 0 & 0\\ 0 & 0 & 0 & 0 & 0 & 0 & 0 & 0 & 0 & 22 & 0 & 0 & 0 & 26 & 0 & 0 & 0 & 0 & 0 & 28\\ 69 & 0 & 0 & 0 & 0 & 0 & 0 & 0 & 0 & 0 & 0 & 0 & 0 & 0 & 0 & 0 & 0 & 0 & 0 & 0\\ 66 & 0 & 0 & 0 & 0 & 0 & 29 & 0 & 0 & 0 & 0 & 0 & 0 & 0 & 0 & 0 & 0 & 0 & 0 & 0\\ 0 & 0 & 0 & 0 & 0 & 37 & 0 & 0 & 0 & 0 & 0 & 0 & 0 & 0 & 0 & 0 & 0 & 28 & 0 & 0\\ 59 & 0 & 0 & 0 & 0 & 0 & 0 & 0 & 0 & 0 & 0 & 26 & 0 & 0 & 0 & 0 & 0 & 0 & 0 & 0\\ 55 & 0 & 0 & 0 & 0 & 0 & 0 & 39 & 0 & 0 & 0 & 0 & 23 & 34 & 0 & 0 & 0 & 0 & 0 & 0\\ 53 & 0 & 0 & 0 & 37 & 0 & 0 & 0 & 0 & 0 & 0 & 0 & 0 & 0 & 31 & 0 & 0 & 0 & 0 & 0\\ 0 & 28 & 0 & 0 & 0 & 0 & 0 & 0 & 0 & 0 & 0 & 0 & 0 & 0 & 0 & 0 & 0 & 0 & 0 & 0\\ 0 & 0 & 0 & 0 & 0 & 0 & 31 & 0 & 0 & 0 & 0 & 0 & 31 & 0 & 0 & 0 & 28 & 0 & 0 & 0\\ 0 & 0 & 0 & 0 & 0 & 0 & 0 & 29 & 0 & 0 & 0 & 20 & 0 & 0 & 0 & 22 & 0 & 0 & 0 & 0\\ 64 & 0 & 0 & 27 & 0 & 0 & 0 & 0 & 0 & 0 & 0 & 0 & 0 & 0 & 35 & 0 & 0 & 0 & 0 & 0\\ 0 & 0 & 0 & 0 & 0 & 0 & 0 & 0 & 0 & 20 & 0 & 0 & 0 & 0 & 0 & 0 & 0 & 0 & 0 & 0\\ 61 & 0 & 0 & 0 & 0 & 0 & 0 & 0 & 36 & 0 & 0 & 0 & 37 & 0 & 0 & 0 & 0 & 0 & 0 & 0\\ 0 & 0 & 0 & 0 & 0 & 0 & 0 & 0 & 0 & 0 & 0 & 0 & 22 & 0 & 0 & 0 & 0 & 0 & 0 & 0\\ 63 & 0 & 0 & 0 & 0 & 0 & 34 & 0 & 0 & 0 & 0 & 0 & 0 & 0 & 0 & 0 & 0 & 0 & 0 & 0\\ 57 & 39 & 0 & 0 & 0 & 38 & 0 & 0 & 0 & 0 & 0 & 0 & 0 & 0 & 0 & 0 & 0 & 0 & 0 & 0\\ 56 & 0 & 0 & 28 & 34 & 0 & 0 & 0 & 0 & 0 & 0 & 0 & 0 & 0 & 0 & 0 & 0 & 0 & 0 & 0 \end{array}\right] \end{array}$$

Finally, to simulate the dominant influence of a central affective source—such as a major social media platform like *TikTok* or *Facebook*—we amplified the contagion effect of the first oscillator (node 1) by an order of magnitude. Specifically, we multiplied the values in the first column of the contagion matrix by a factor of 10. This adjustment reflects the asymmetry typically observed in real-world complex social networks, where a single highly influential agent can strongly modulate the emotional states of a large number of followers.

This modified contagion matrix, where the influence of node 1 has been enhanced tenfold relative to all other interactions, constitutes our **Example #9**. It serves to illustrate how dominant sources of emotional input can shape the emergent collective dynamics across a decentralized system.

## 4 Results and discussion

The following results present simulations carried out using the numerical methods and computational configurations described in the previous section. The experiments were designed to explore the effects of different damping coefficients and initial conditions on the temporal evolution of emotional states in our extended Russell circumplex model.

The graphical results are organized in a matrix format with three rows and three columns, corresponding to variations in damping and to the key emotional variables tracked:

- **Columns:**
  1. **Column 1:** The temporal evolution of the radius *r*(*t*), which reflects affective intensity, i.e., the distance from neutrality. Arousal is represented separately by the vertical coordinate A(t)=r(t)sinθ(t).
  2. **Column 2:** Temporal evolution of the angular component θ(t), corresponding to the valence-arousal direction in the circumplex plane.
  3. **Column 3:** Emotional trajectories in the complex circumplex plane. Each trajectory begins at a point marked with an **X** (initial state) and ends at a large black dot (final state), allowing visualization of the full emotional transition path over time.
- **Rows:** 1. **Row a:** Simulations using a damping coefficient of *b* = 0.01.
  2. **Row b:** Simulations with *b* = 0.1.
  3. **Row c:** Simulations with *b* = 1.0.

It is important to note that the damping coefficients used are equal for both the radial and angular components ($b_{r}=b_{\theta }$) in each simulation. This layout provides a comprehensive comparison of how different levels of emotional inertia affect both the time evolution and spatial trajectories of emotional states within the Russell circumplex framework. Additionally, for reference purposes, the integration intervals and number of sampling points used in the experiments reported in this work are summarised in Table 2. The stored output-grid density was selected for plotting and post-processing; it did not set the solver’s internal adaptive steps or error tolerances.

**Table 2:** Numerical integration intervals and output sampling used in the reported simulations. The output grid specifies stored values for plotting and is distinct from the solver’s internal adaptive steps.

Scenario	Integration interval	Number of output points
Examples 1–3	0≤t≤50	800
Example 4, Fig 9, *b* = 0.01	0≤t≤50	5000
Example 4, Fig 9, *b* = 0.1	0≤t≤50	4000
Example 4, Fig 9, *b* = 1.0	0≤t≤50	2000
Example 5, Fig 10	0≤t≤50	5000
Example 5, Fig 11, *b* = 0.01	0≤t≤200	20000
Example 5, Fig 11, *b* = 0.1	0≤t≤100	10000
Example 5, Fig 11, *b* = 1.0	0≤t≤10	1000
Example 6, Fig 12	0≤t≤20	800
Example 7, Fig 13	0≤t≤30	2000
Examples 8–9	0≤t≤20	800

### 4.1 Scenario 1: Transitivity

In this first example, we simulate emotional transitivity in a minimal system of three coupled oscillators. The initial conditions are set as follows: oscillator 1 (the emotional leader) begins in the emotional state *“happy”*, but its target attractor is set to *“angry”*; oscillator 2 (the intermediary) starts at *“bored”*; and oscillator 3 (the observer) begins in *“distressed”*. These three distinct starting points provide a diverse affective context from which to analyze convergence and influence dynamics.

**Fig 6:**
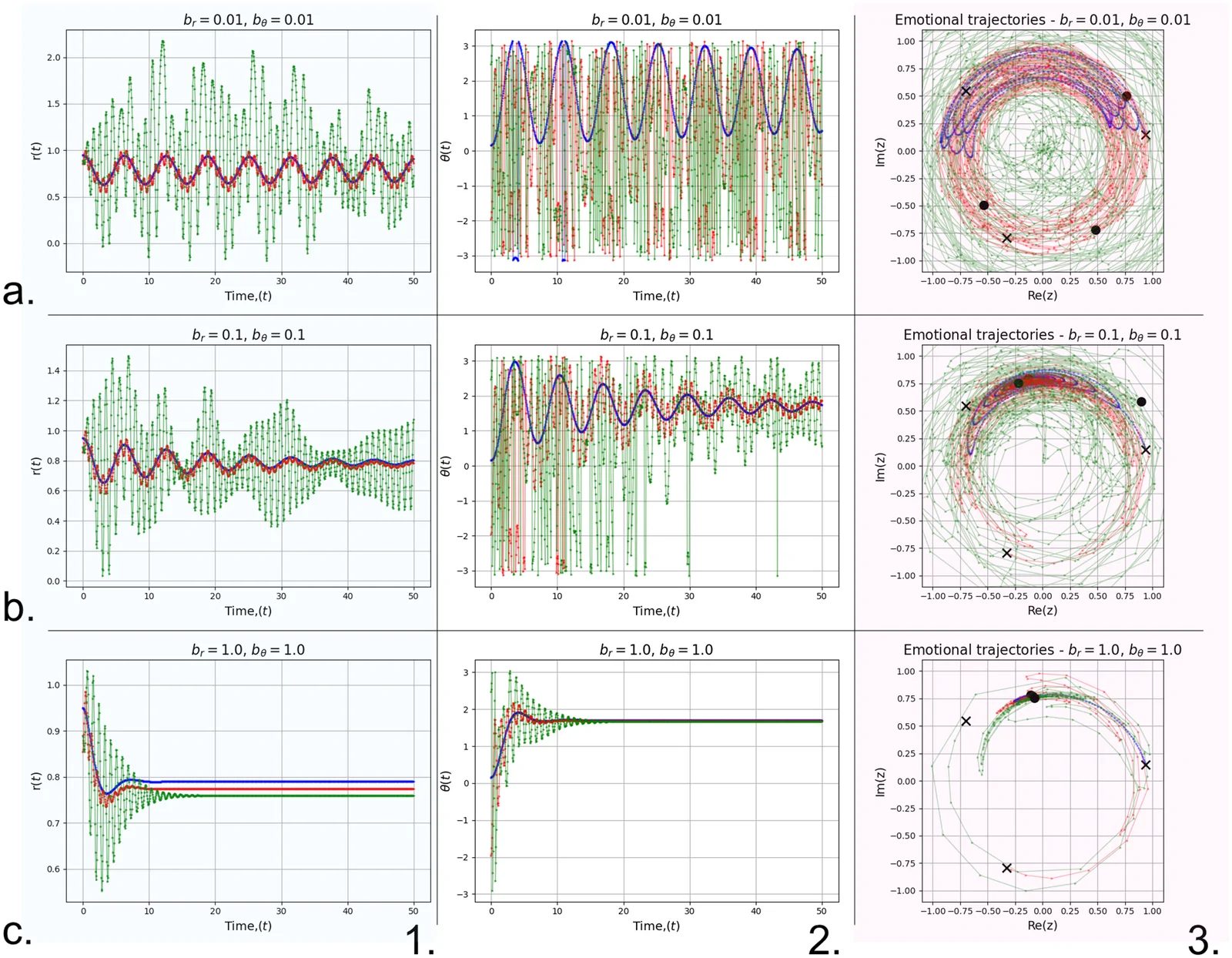
Simulation results for Example 1, with symmetric coupling between oscillators: oscillator 1 (leader, shown in blue line), oscillator 2 (mediator, shown in red line), and oscillator 3 (observer, shown in green line). Each row corresponds to a different damping coefficient (*B* = 0.01, 0.1, and 1.0 from top to bottom). Columns represent the temporal evolution of the emotional radius and phase. As damping increases, the system exhibits progressively stronger synchronization, with all oscillators approaching the emotional attractor associated with *Angry*. This indicates efficient emotional transmission under symmetric connectivity.

**Fig 7:**
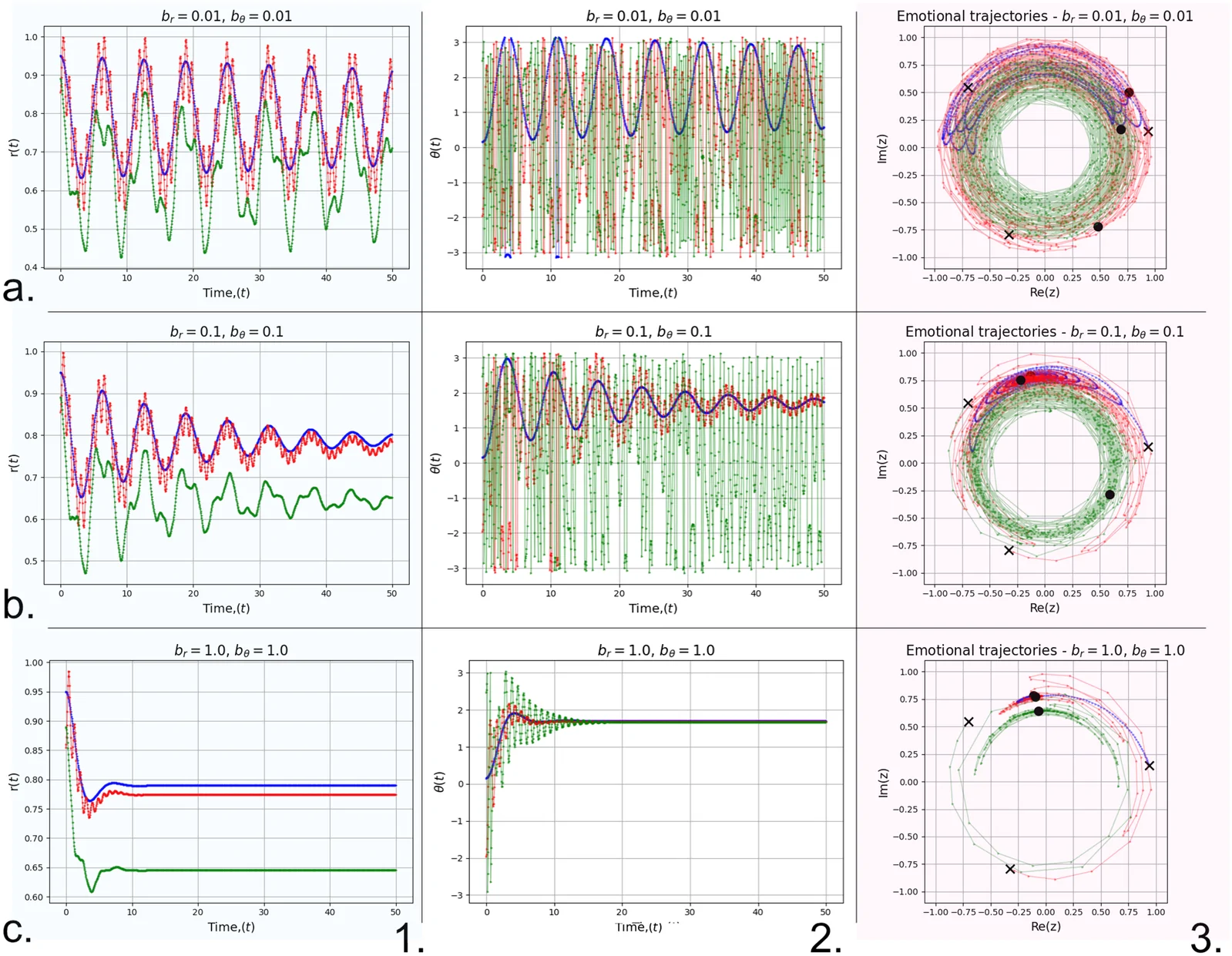
Simulation results for Example 2, where coupling between oscillator 1 (leader, blue line) and oscillator 2 (mediator, red line) is strong, but the connection between oscillator 2 and oscillator 3 (observer, green line) is weak. This asymmetry disrupts full emotional propagation. In low damping (*b* = 0.01), the observer exhibits desynchronized oscillations with high amplitude variation. Even in higher damping conditions (*b* = 1.0), only partial convergence is observed, emphasizing the necessity of consistent connectivity throughout the network to achieve full emotional alignment.

**Fig 8:**
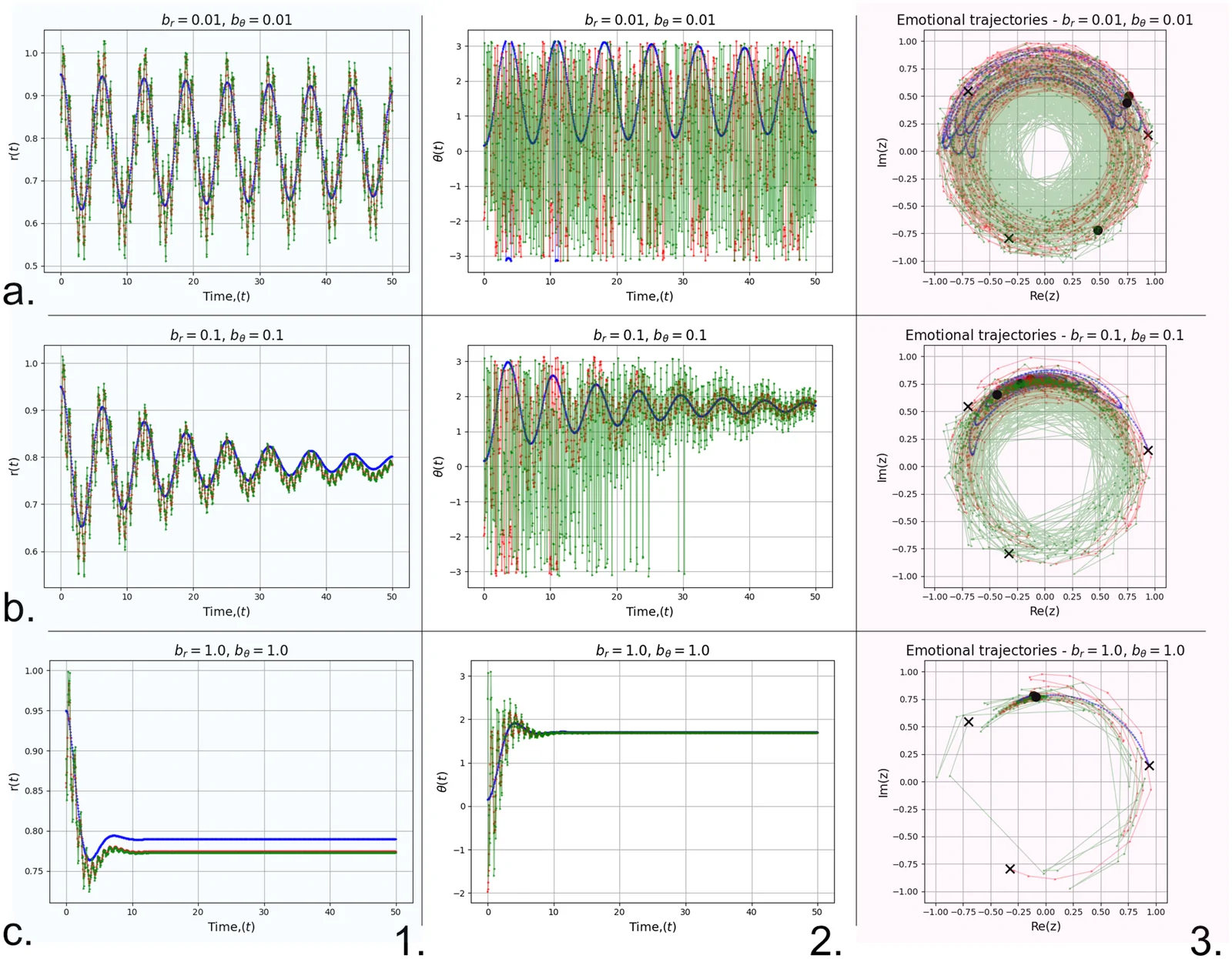
Simulation results for Example 3, featuring weak coupling between oscillator 1 (leader, blue line) and oscillator 2 (mediator, red line), and strong coupling between oscillator 2 and oscillator 3 (observer, green line). Despite the reduced leader influence, the strong mediator-observer connection allows effective emotional transmission. In all damping conditions—particularly at *b* = 1.0—oscillators 2 and 3 closely follow the leader’s trajectory toward the *Angry* attractor. This highlights the essential role of mediators as emotional integrators in distributed networks.

The evolution of the system is shown in Figs 6–8, where the emotional radius and angle of each oscillator are tracked over time, as well as their corresponding trajectories in the Russell complex plane.

In the figure, oscillator 1 (the leader) is plotted in **blue** with a solid line, indicating its central role in emotional influence. Oscillator 2 is shown with a **red** solid line, and oscillator 3 with a **green** solid line as well. These color and style distinctions allow for easy identification of the dynamics of each node throughout the simulation.

The results reveal a clear pattern of emotional transitivity: the state of oscillator 1 gradually influences the trajectory of oscillator 2, which in turn exerts a delayed and moderated influence on oscillator 3. This example demonstrates the basic emotional contagion process and the potential for indirect influence via intermediaries, illustrating how the model represents layered transmission of affective states through a structured network.

#### 4.1.1 Example 1.

In the first simulation (Fig 6), we implemented a symmetrical coupling architecture, where oscillator 1 (the emotional leader or source) is connected to oscillator 2 (the mediator), which in turn is equally coupled to oscillator 3 (the observer). This structure is designed to emulate hierarchical emotional influence within a linear social chain. In the top row (*b* = 0.01), the leader oscillator demonstrates sustained oscillatory behavior, fluctuating between its initial emotional state and the attractor corresponding to the discrete emotional label *Angry*. Due to the minimal damping, the amplitude and phase fluctuations persist over time, which results in the partial transmission of emotional influence to the subsequent nodes. Oscillator 3 exhibits clear phase and amplitude deviations, reflecting a lack of full emotional synchronization. As the damping increases (*b* = 0.1, middle row), the leader gradually stabilizes toward the *Angry* attractor, which leads to a more coherent propagation of emotional state across the chain: both the mediator and observer increasingly align their trajectories with the leader’s state. In the bottom row (*b* = 1.0), all three oscillators converge to a vicinity of the same emotional attractor. However, small divergences—especially in oscillator 3—remain, likely due to the slight cumulative phase delays introduced by the two-step transmission. These results suggest that under symmetric and moderate coupling conditions, higher damping enhances emotional coherence and facilitates stable affective alignment across a social system.

#### 4.1.2 Example 2.

The second simulation (Fig 7) explores the asymmetry of influence by weakening the coupling between oscillator 2 and oscillator 3 while maintaining strong coupling between the leader (oscillator 1) and the mediator (oscillator 2). This setup simulates emotional networks in which intermediate agents are well aligned with the leader but poorly connected to peripheral observers. In the low damping condition (*b* = 0.01), the emotional leader again exhibits sustained oscillations, but while the mediator mirrors this activity with moderate fidelity, the observer becomes increasingly decorrelated—both in phase and amplitude—over time. This lack of influence on oscillator 3 results in emotional fragmentation, as visualized by the diverging trajectories in the third column of the figure. Even as damping increases (*b* = 0.1 and *b* = 1.0), only the leader and mediator appear to converge toward the emotional attractor. Oscillator 3 remains offset in both angular and radial dimensions, occupying a region near, but not fully overlapping, the *Angry* state. This suggests that effective emotional contagion requires not only leader influence but also sufficient downstream coupling; emotional influence is not inherently transitive unless structural connectivity supports it. Psychologically, this could represent scenarios in which distal group members fail to internalize collective affective states due to weak interpersonal ties or communicative bottlenecks.

#### 4.1.3 Example 3.

The third simulation (Fig 8) reverses the previous asymmetry: the coupling between oscillator 1 and 2 is weakened, while that between oscillator 2 and 3 is strengthened. These tests whether a strongly engaged intermediary can compensate for a weak leader connection and still facilitate emotional alignment downstream. In the low damping condition (*b* = 0.01), the mediator captures the general structure of the leader’s oscillations, although with attenuated amplitude and increased smoothing. Remarkably, oscillator 3, which is not directly connected to the leader, demonstrates a higher degree of synchronization with the leader via the strong influence of oscillator 2. This result is even more prominent in the high damping condition (*b* = 1.0), where all three oscillators stabilize near the *Angry* attractor, despite the weakened direct influence from the leader. The effectiveness of this transmission highlights the critical role of mediators in emotional networks: when properly coupled, intermediaries can serve as emotional amplifiers and integrators, transmitting and reinforcing affective states even under suboptimal leadership dynamics. From a psychological and social neuroscience perspective, this model illustrates how emotionally attuned agents—such as empathetic team members or emotionally intelligent facilitators—can bridge the gap between emotionally distant individuals and foster collective emotional coherence.

#### 4.1.4 Example 4.

**Fig 9:**
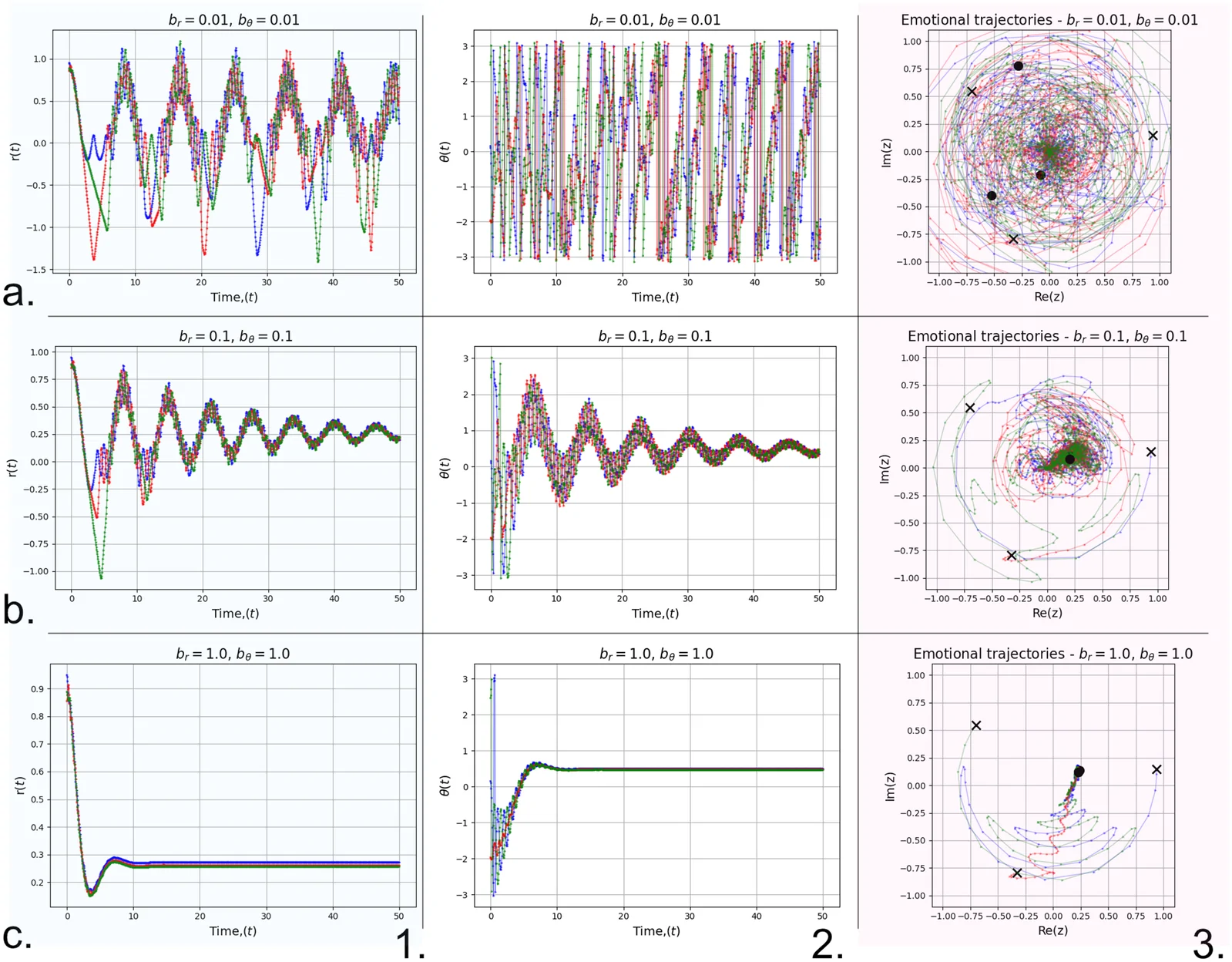
Simulation results for Example 4: reciprocal three-agent chain. Oscillator 2 (central agent, red line) is bidirectionally coupled to oscillator 1 (blue line) and oscillator 3 (green line) with equal strength; no direct coupling exists between oscillators 1 and 3. The three rows represent increasing damping levels (*b* = 0.01,0.1,1.0). The trajectories tend toward a shared state that remains distinct from the Angry attractor assigned to oscillator 1, illustrating network-mediated convergence through the central agent. For *b* = 0.01 and *b* = 0.1, portions of the radial traces below zero indicate loss of admissibility of the unconstrained radial coordinate and are not interpreted as valid affective intensities.

In this simulation (Fig 9), a reciprocal three-agent chain was implemented. Oscillator 2 is bidirectionally coupled to oscillators 1 and 3 with equal coupling strength, while oscillators 1 and 3 are not directly coupled. This architecture represents a network in which reciprocal influence is mediated through a central agent rather than distributed through direct connections between all pairs.

In the low damping condition (*b* = 0.01, top row), the oscillators display highly similar trajectories in both emotional radius and angular phase. Despite their initial conditions and transient excursions across various regions of the circumplex model, all three trajectories converge toward closely located points in emotional space, as seen in the third column. This suggests that mutual bidirectional influence stabilizes the system into a shared emotional state, though this state does not precisely align with the Angry attractor assigned to oscillator 1. As damping increases (*b* = 0.1, middle row), synchronization among the oscillators becomes more pronounced. They settle into a common emotional state that is notably distant from the *Angry* attractor originally associated with oscillator 1. This shift indicates that, in this reciprocally coupled chain, the final emotional state emerges from a dynamic negotiation among nodes rather than from top-down transmission.

For *b* = 0.01 and *b* = 0.1, the direct radial baseline crosses $r_{i}=0$ during portions of the transient response. These negative values are not interpreted as negative affective intensity and do not represent valid circumplex states. Rather, they indicate loss of radial admissibility of the unconstrained oscillator under these parameter regimes. The trajectories are retained here to expose this limitation and to motivate the constrained radial formulation introduced in Section 2.2.

In the high damping case (*b* = 1.0, bottom row), the system exhibits strong coherence: all oscillators evolve in a nearly identical manner, forming a synchronized cluster in the emotional state space. However, this final state remains intermediate, indicating that mutual coupling reduces the dominance of any single node’s attractor. Psychologically, such mutual influence networks may correspond to emotionally cohesive groups where affective consensus arises not from leadership, but from distributed regulation and compromise.

#### 4.1.5 Example 5.

**Fig 10:**
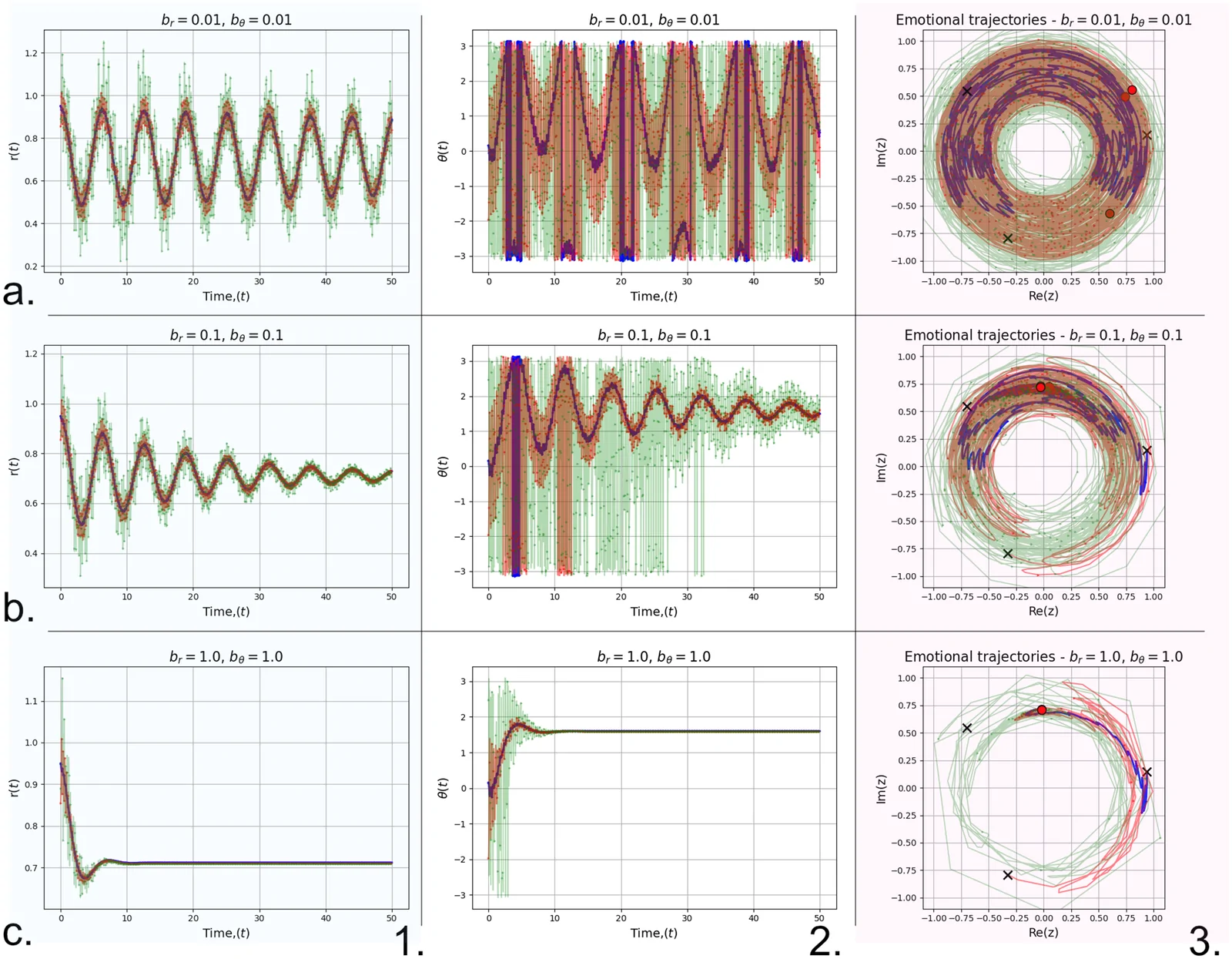
Simulation results for Example 5: Asymmetrical coupling favoring the forward direction from oscillator 1 to 2 to 3. Oscillator 1 (leader, **blue line**) exerts strong influence over oscillator 2 (**red line**), which in turn strongly affects oscillator 3 (**green line**). Feedback coupling is weak. Each row corresponds to increasing damping levels (*b* = 0.01, 0.1, 1.0). Over time, all oscillators converge toward the leader’s emotional attractor, *Angry*, particularly in the higher damping conditions. This illustrates effective emotional leadership and top-down convergence enabled by directional dominance in coupling.

The fifth simulation (Fig 10) retains the triadic structure but introduces asymmetry in the coupling strengths. Specifically, the directional influence from oscillator 1–2–3 is made substantially stronger than the reverse paths. This setup resembles a cascade hierarchy, in which emotional influence is primarily top-down but still permits some feedback. In the low damping scenario (*b* = 0.01), the initial divergence of the oscillators is more pronounced, yet a clear trend toward the leader’s attractor (*Angry*) is already noticeable. The influence is not purely unidirectional—oscillators do exert minor reciprocal effects—but the strong forward coupling ensures a dominant emotional drive from the leader. This trend becomes more robust in medium (*b* = 0.1) and high damping conditions (*b* = 1.0), where all three oscillators ultimately converge toward the emotional attractor of oscillator 1. The third column of each row shows this alignment clearly, with emotional states clustering tightly around the *Angry* region. This result supports the hypothesis that directional coupling with appropriate damping enables effective emotional leadership, where the affective state of a central agent can reliably shape the group’s emotional trajectory. Such a model aligns well with hierarchical organizational or neural systems, where top-down regulation plays a critical role in stabilizing collective affect or behavior.

In this model of emotional contagion, the damping coefficient regulates the pace of synchronisation, that is, how quickly individuals converge toward a shared emotional state. When the damping is strong, emotional fluctuations decay rapidly, and the group aligns more efficiently with the leader’s emotional attractor. Over time, this dynamic ensures that the emotional tone established by the leader becomes the dominant reference point for all members, emphasising the central role of leadership in shaping the collective mood. From a psychological perspective, the damping parameter may be interpreted as the degree of openness or receptivity that individuals show toward the leader’s emotions. Higher receptiveness enables faster convergence, highlighting that effective emotional leadership depends not only on the leader’s directional dominance but also on the followers’ readiness to internalise the affective state being projected.

**Fig 11:**
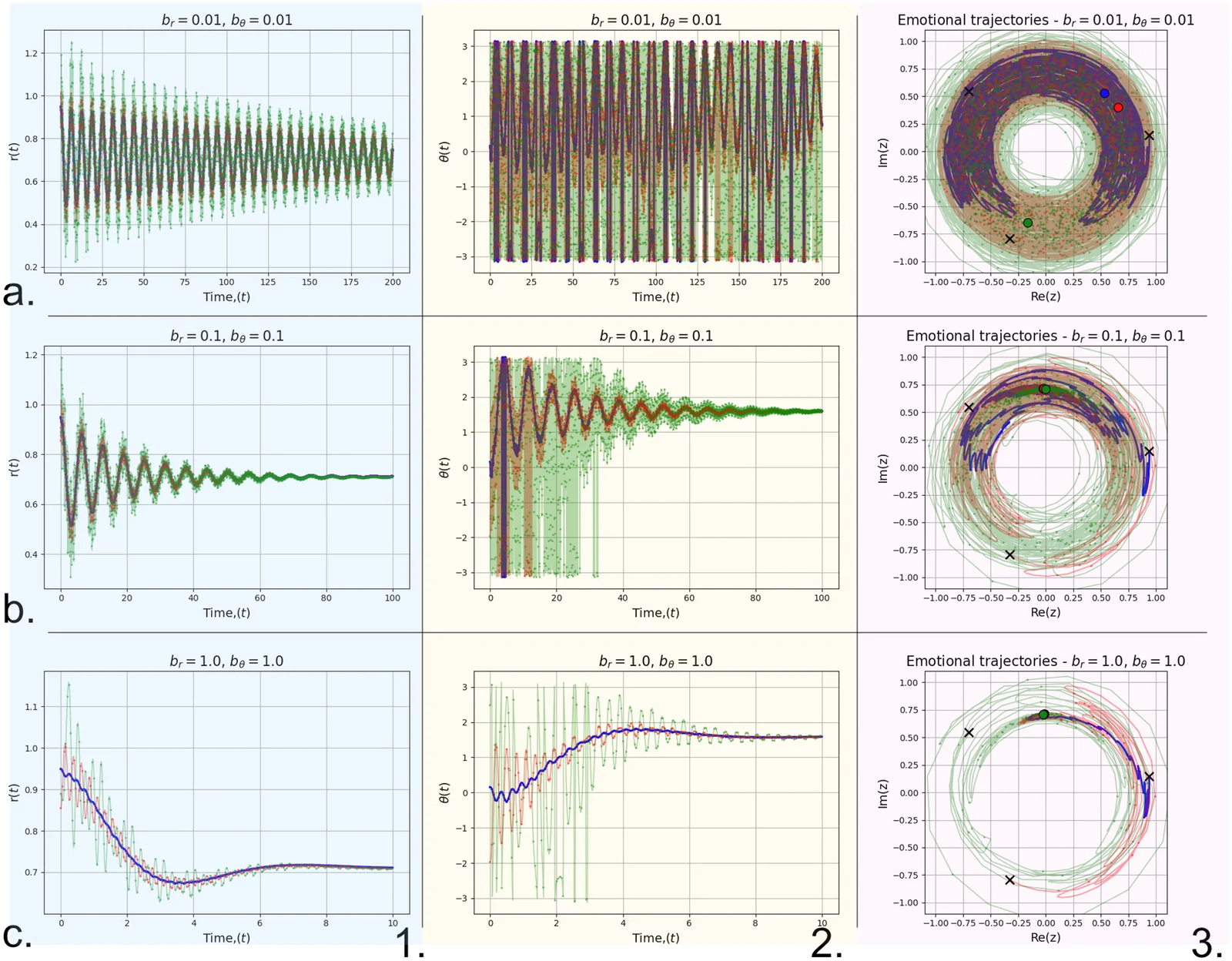
Comparison of synchronisation times for Example 5: Asymmetrical coupling favouring the forward direction from oscillator 1 → 2 → 3. Emotional trajectories are shown for oscillator 1 (leader, blue line), oscillator 2 (red line), and oscillator 3 (green line) under weak feedback coupling. Each row corresponds to an increasing damping level (*b* = 0.01, 0.1, 1.0). The results show that, over time, all oscillators converge toward the leader’s emotional attractor, with convergence occurring more rapidly under higher damping conditions. This behaviour illustrates how the damping effect can represent the ease and speed with which individuals adjust emotionally and absorb influence from a leader.

This process is illustrated in Fig 11, where the model is applied under different damping conditions, with the time evolution adjusted for comparison. The simulations clearly show that low damping is associated with longer times to achieve synchronisation, as individuals take more time to align with the leader’s state. In contrast, higher damping coefficients produce a more rapid adjustment, with the three followers quickly converging toward the leader’s emotional trajectory. These contrasting outcomes illustrate how variations in receptiveness fundamentally shape the speed and effectiveness of emotional convergence within a group.

### 4.2 Scenario 2: Leader-driven coupling dynamics

#### 4.2.1 Example 6.

**Fig 12:**
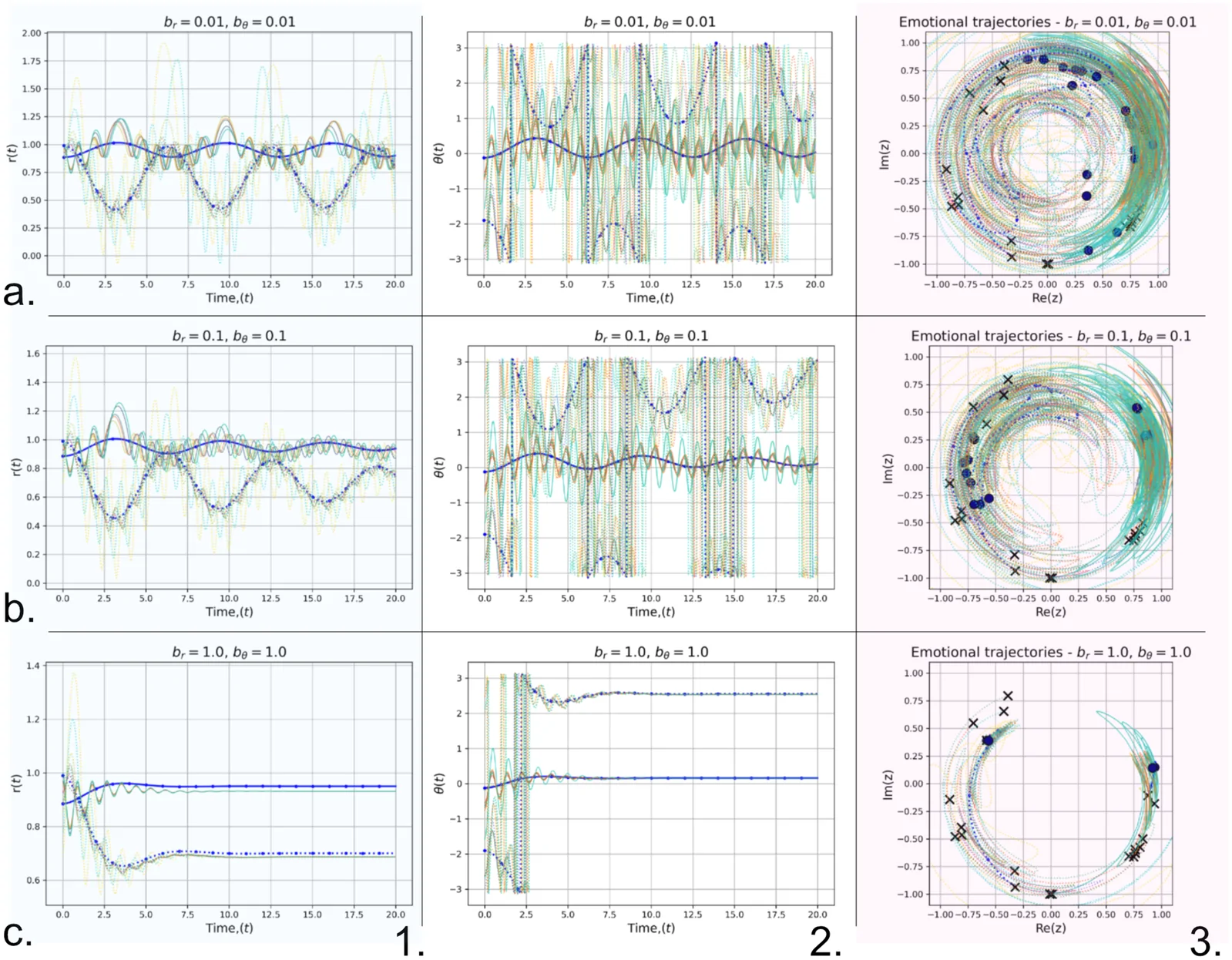
Simulation results for Example 6: Two independent groups led respectively by oscillator 1 (leading oscillators 2–10, blue solid line) and oscillator 11 (leading oscillators 12–20, blue dotted line in group 2). Each leader is uncoupled from the other, but strongly coupled to their own group members. Oscillator 1 moves toward the *Happy* emotional attractor, while oscillator 11 moves toward *Frustrated*. Rows represent increasing damping coefficients (*b* = 0.01, 0.1, 1.0). At low *b*, persistent oscillations prevent convergence; at higher *b*, each group stabilizes around its respective leader’s emotional state, forming two distinct, internally coherent emotional clusters.

In this simulation scenario (Fig 12), we examine a system composed of two independent groups, each governed by a distinct leader: oscillator 1 leads oscillators 2–10, while oscillator 11 leads oscillators 12–20. Importantly, no coupling exists between the two groups, allowing us to study the internal synchronization dynamics of each group under independent emotional leadership. Oscillator 1 converges toward the emotional attractor labeled *Happy*, whereas oscillator 11 approaches the state *Frustrated*. In the top row (*b* = 0.01), minimal damping permits persistent oscillatory behavior around the respective attractors. The group members oscillate near their leader’s trajectory, yet they fail to settle into a final emotional state. This is clearly illustrated in the third column, where emotional positions remain dynamic and divergent. In the middle row (*b* = 0.1), moderate damping improves local synchronization: group members begin to settle more closely around their respective leaders’ attractors, but still exhibit mild deviations. The most striking result occurs in the bottom row (*b* = 1.0), where both groups display strong intra-group coherence and stabilization. The two clusters of oscillators independently converge to distinct, emotionally meaningful endpoints—*Happy* and *Frustrated*—determined by the internal influence of their leaders. This simulation captures a psychologically plausible group phenomenon: in the absence of intergroup interaction, cohesive emotional identity can emerge internally, shaped by leadership, with different subgroups settling into emotionally polarized yet internally consistent states.

#### 4.2.2 Example 7.

**Fig 13:**
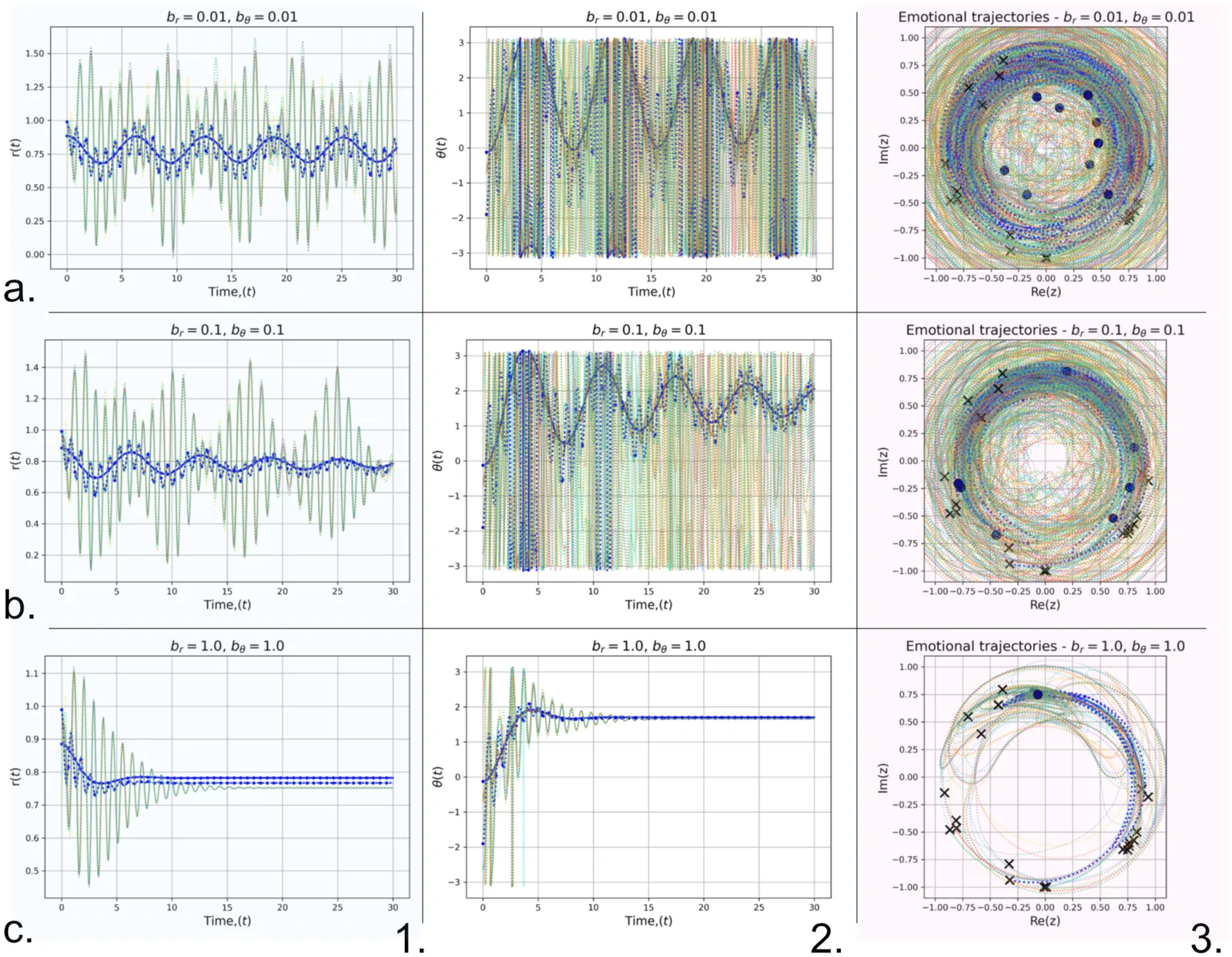
Simulation results for Example 7: Two competing groups where the leaders —-oscillator 1 and oscillator 11 (shown in blue solid and blue dashed lines respectively)– are directly coupled, modeling inter-group emotional conflict. Oscillator 1 initially exerts dominant influence, pulling oscillator 11 and its group (oscillators 12–20, **green, yellow, and red lines**) towards its emotional attractor. With increasing damping (*b* = 0.01, 0.1, 1.0 from top to bottom), the system shifts from irregular partial alignment to near-complete network convergence. At high *b*, all oscillators align in phase and amplitude, potentially around a high-arousal emotional state such as *Angry*, suggesting collective emotional escalation driven by leader conflict.

In the final simulation (Fig 13), we introduce direct interaction between the two group leaders—oscillators 1 and 11—initiating a scenario of emotional conflict or negotiation between competing leaders. This represents a shift from isolated group dynamics to a model of intergroup influence. In the low damping condition (*b* = 0.01), oscillators from both groups exhibit irregular trajectories and high variability, with the dominant influence of oscillator 1 pushing oscillator 11—and, by extension, its followers—toward its own attractor state. This is evident in the early-phase asymmetry shown in the figure, where group 2 begins to deviate from its initial orientation. As damping increases (*b* = 0.1 and especially *b* = 1.0), mutual influence between the two leaders becomes more apparent and leads to system-wide synchronization: the emotional states of all 20 oscillators begin to converge. In the high damping condition, the entire network settles into a shared emotional phase, often centered around the dominant leader’s attractor. If this attractor corresponds to a high-arousal, confrontational emotion such as *Angry*, the result may be collective escalation, with both groups entering a synchronized confrontational state. This reflects real-world phenomena where interleader conflict can escalate into broader intergroup tension or polarization. Such dynamics underline the psychological and social risk of emotional contagion when group leaders are in direct opposition, and how their personal states can ripple through and reshape the emotional landscape of entire collectives.

### 4.3 Scenario 3: Complex network

#### 4.3.1 Example 8.

**Fig 14:**
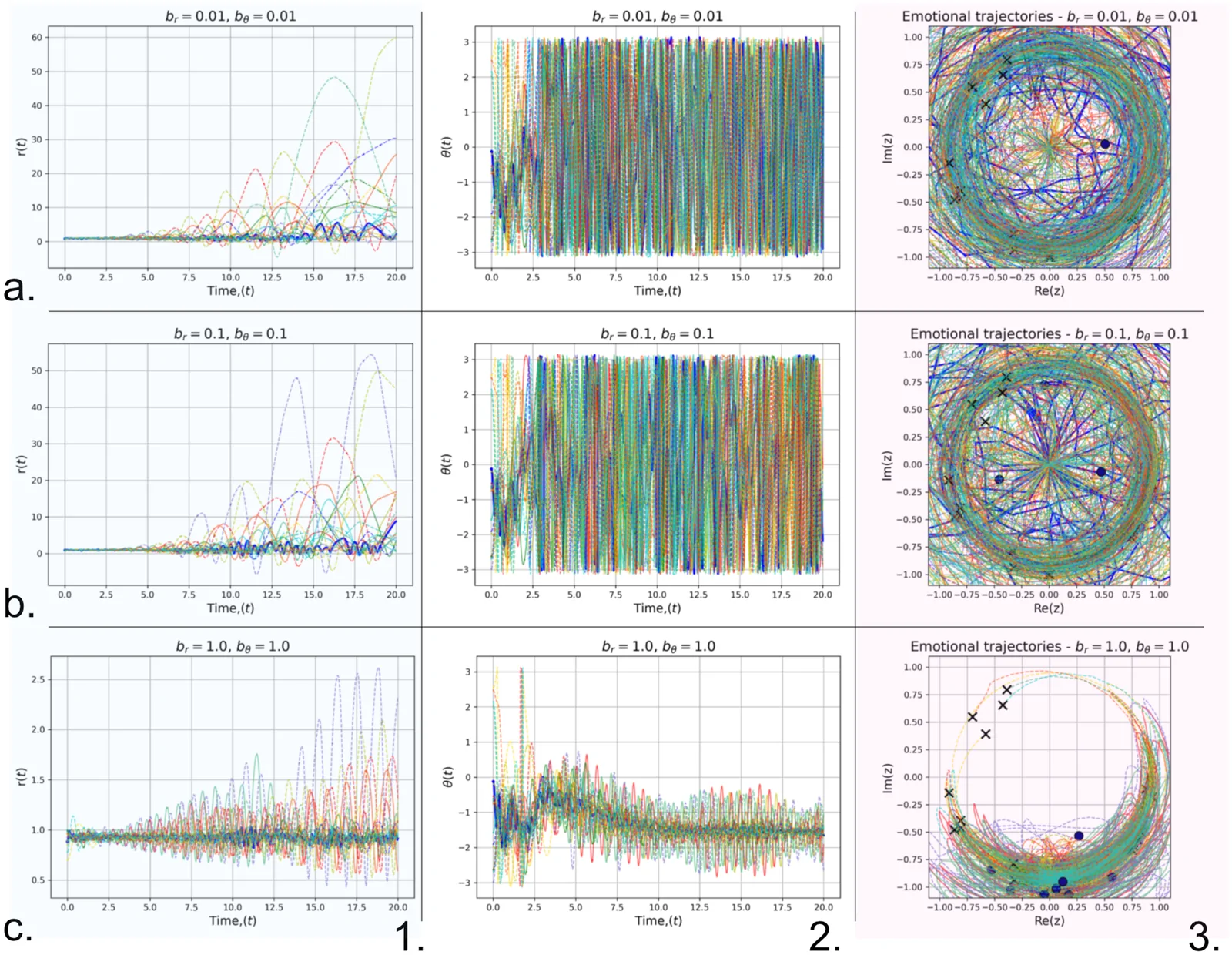
Simulation results for Example 8: A complex, weakly coupled network representing a distributed system like a social media platform (e.g., Facebook or TikTok). The leader (in **blue solid line**) exerts diffuse and weak influence over the network, with similarly weak coupling among individual nodes (in **yellow, red, green, and cyan dashed lines**). Rows correspond to increasing damping levels (*b* = 0.01, 0.1, 1.0). No global synchronization occurs; instead, oscillators exhibit emotional divergence and persistent fluctuations. In the high-*b* case, oscillatory amplitudes increase over the simulated interval, producing greater state dispersion, described in this study as oscillatory amplification. The third column shows the distribution of final states across the circumplex, highlighting the absence of a unified emotional outcome.

In this simulation (Fig 14), we modeled a complex, weakly coupled emotional network representative of modern digital social platforms such as Facebook or TikTok. Here, the network is composed of many nodes (oscillators) with approximately uniform and low-strength bidirectional coupling—both from a central source (analogous to a network-wide influence or platform algorithm) and among peers. This configuration results in a system where no single emotional attractor dominates. In the low damping condition (*b* = 0.01), oscillators fail to synchronize, exhibiting sustained and divergent oscillations across the emotional space. This is evident in both the radius and phase dynamics, where no coherent emotional state is reached. As shown in the third column, final states remain scattered across the circumplex, illustrating the emotional heterogeneity produced by weak and diffuse coupling.

As the damping increases (*b* = 0.1 and *b* = 1.0), the system does not immediately settle; instead, several trajectories exhibit increasing oscillatory amplitudes over the simulated interval. We describe this behaviour as oscillatory amplification rather than resonance, because no periodic-forcing frequency sweep or frequency-response peak is evaluated in the present study. The corresponding quantitative diagnostic is the amplification ratio $A_{osc}$ defined in Section 2.3. This counterintuitive behavior arises from the overlapping effects of many weak influences, which can interact constructively producing emotional volatility. The final states remain diverse and fragmented, with no consensus emerging, even at high *b*. Psychologically, this reflects the turbulent and often incoherent emotional dynamics of large-scale social media systems, where individuals are influenced simultaneously by many weak signals without a clear organizing emotional force.

#### 4.3.2 Example 9.

**Fig 15:**
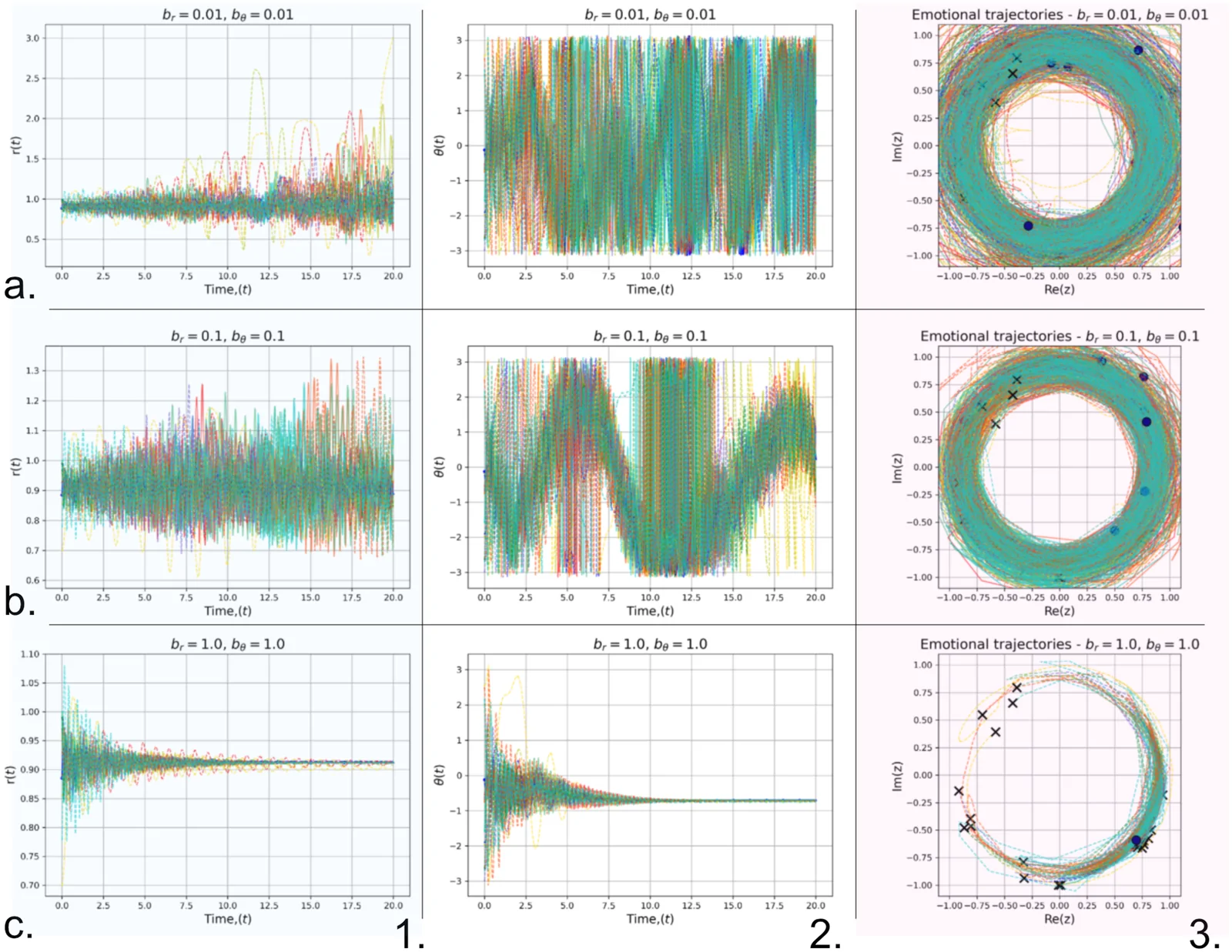
Simulation results for Example 9: A distributed emotional network dominated by a strongly coupled leader node (in blue solid line), with weak peer-to-peer connections among other nodes (in yellow, red, green, and cyan dashed lines). Rows represent increasing damping levels (*b* = 0.01, 0.1, 1.0). As *b* increases, the leader’s influence promotes stronger global emotional alignment. By *b* = 1.0, most oscillators converge near the leader’s emotional attractor, illustrating how a dominant agent—such as a political figure or viral influencer—can override network heterogeneity and shape collective affect. Final positions in the third column show convergence toward a unified emotional state.

In contrast, Fig 15 presents a scenario in which one highly influential leader (e.g., a viral content creator or political figure) is embedded within the same kind of distributed network, but now exerts disproportionately strong coupling on all other nodes. Peer-to-peer couplings remain weak, but the leader’s influence is dominant. This creates a structural asymmetry, modeling how charismatic or algorithmically amplified figures can shape emotional dynamics at scale. Under low damping (*b* = 0.01), the network exhibits partial alignment toward the leader’s emotional attractor, though transient variability remains. As the damping increases (*b* = 0.1 and especially *b* = 1.0), the influence of the leader becomes more effective in driving global convergence. By the high damping condition, nearly all nodes have aligned with the leader’s emotional state, or have stabilized in a nearby region of the emotional space. Even when individual oscillators begin with distinct initial conditions or conflicting intrinsic attractors, the gravitational pull of the leader causes affective consensus. The final emotional state of the network may reflect either the leader’s target state or a negotiated compromise between leader-driven influence and node-specific tendencies. This illustrates a psychologically relevant mechanism for emotional alignment in political, cultural, or algorithmically curated contexts—where dominant figures or systems can override emotional diversity and promote affective homogeneity through asymmetric coupling.

## 5 Conclusions and future directions

This study presents an exploratory computational framework for representing emotional contagion through coupled radial and angular dynamics in the valence–arousal plane of the Circumplex model of Affect. The phasor formulation provides a mathematically explicit means of generating and comparing affective trajectories under prescribed attractors, damping coefficients, forcing terms, coupling strengths, and network structures. The simulations produce qualitative model behaviours including phase alignment, contraction or dispersion of collective affective states, leader-induced tracking, and oscillatory amplification. These outcomes are operationalized using the quantitative measures defined in Section 2.

Although the present study is not an empirical validation or predictive test of human affective dynamics, by formalizing emotions as complex-valued phasors evolving over time, the model captures not only the intensity and direction of affective states but also their trajectories, regulatory patterns, and susceptibility to interpersonal influence. The oscillator components and coupling mechanisms are mechanistic analogies, and the simulations do not establish a direct neurobiological mechanism of emotional contagion. Psychological, clinical, and social interpretations should therefore be understood as hypotheses generated by the model rather than as validated explanations or intervention tools.

Psychologically, the simulations illustrate how emotional inertia, damping, and social coupling can shape affective synchrony and divergence within the proposed mathematical framework. Phenomena such as emotional dragging, leader-induced tracking, oscillatory amplification, and collective convergence are represented here as model-generated dynamical patterns governed by internal baselines and interpersonal coupling. From a clinical or social-intervention perspective, these results should be interpreted as theoretical simulations rather than validated predictive tools. However, the model proposed could provide theoretical tools to analyze therapeutic co-regulation, emotional dysregulation, and affective polarization in group therapy or social trauma contexts. Future empirical work will be required to determine whether the parameters of the model can be reliably estimated from behavioural, physiological, or interactional data.

The oscillatory formulation is compatible with the broader view that affective regulation involves temporally structured neural, autonomic, and interpersonal processes. However, the present simulations do not establish a direct biological mechanism. The model should therefore be understood as a mechanistic analogy and generative simulation framework, not as a validated neurobiological model of emotional contagion.

At the computational level, the use of first-order differential systems and coupling matrices opens the door to scalable, agent-based simulation of emotional ecosystems. It also enables integration with real-time sensor data and emotion-aware technologies, including interactive avatars, therapeutic chatbots, and multi-agent simulations of crowd behavior or social networks. The coupling matrices are static, whereas in real-world interactions, emotional influence is dynamic, context-sensitive, and often asymmetric. Additionally, while the circumplex space is a useful abstraction, it cannot fully account for discrete emotion theories or culture-specific emotional constructs. Finally, the model does not yet incorporate noise, learning, or plasticity, which are critical to long-term affective adaptation.

Nonetheless, the model presents several important limitations. It assumes that emotional states can be reduced to planar oscillatory dynamics, abstracting away the rich dimensionality of human affective life, including memory, semantic content, social context, and higher-order cognition.

A further limitation is that the baseline polar dynamics are separable: affective intensity $r_{i}(t)$ and affective direction $\theta_{i}(t)$ are governed by distinct dynamical channels and are coupled only through the reconstruction of the observable valence–arousal coordinates. This assumption is useful for interpretability and parameter identifiability, but it cannot represent all forms of intensity–direction feedback. For example, high arousal may accelerate, delay, or bias transitions in valence, and the current valence–arousal quadrant may modulate radial amplification or damping. Future extensions should therefore estimate nonseparable terms that allow $r_{i}(t)$, $\theta_{i}(t)$, and the Cartesian coordinates $V_{i}(t)$ and $A_{i}(t)$ to influence one another dynamically.

A natural extension of the present baseline is to include explicit feedback between affective intensity and affective direction through a nonseparable radial–angular formulation. A general form is


$$\begin{array}{c} {\dot{r}}_{i}(t)=v_{i}(t), \end{array}$$ (43)

$$\begin{array}{c} {\dot{v}}_{i}(t)=-b_{r}v_{i}(t)-\left[r_{i}(t)-r_{i}^{*}\right]+\sum_{j\text{≠}i}K_{ij}^{r}\left[r_{j}(t)-r_{i}(t)\right]+F_{i}(t)+\Phi_{i}^{r}\left(r_{i},\theta_{i},v_{i},\omega_{i}\right), \end{array}$$ (44)

$$\begin{array}{c} {\dot{\theta }}_{i}(t)=\omega_{i}(t), \end{array}$$ (45)

$$\begin{array}{c} {\dot{\omega }}_{i}(t)=-b_{\theta }\omega_{i}(t)-\sin\left(\theta_{i}(t)-\theta_{i}^{*}\right)+\sum_{j\text{≠}i}K_{ij}^{\theta }\sin\left(\theta_{j}(t)-\theta_{i}(t)\right)+\tau_{i}(t)+\Phi_{i}^{\theta }\left(r_{i},\theta_{i},v_{i},\omega_{i}\right). \end{array}$$ (46)

Here, $\Phi_{i}^{r}$ represents directional modulation of intensity dynamics, whereas $\Phi_{i}^{\theta }$ represents intensity modulation of angular dynamics. For example, $\Phi_{i}^{r}$ could encode quadrant-dependent amplification or damping of intensity, while $\Phi_{i}^{\theta }$ could encode arousal- or intensity-dependent acceleration of affective transitions. The separable model used in the present simulations is recovered by setting


$$\begin{array}{c} \Phi_{i}^{r}=\Phi_{i}^{\theta }=0. \end{array}$$ (47)

This extension is especially relevant for high-arousal transitions, where changes in the signed arousal coordinate


$$\begin{array}{c} A_{i}(t)=r_{i}(t)\sin\theta_{i}(t) \end{array}$$

may alter the rate or direction of movement through the circumplex. However, these feedback functions introduce additional parameters and nonlinearities; their reliable use requires richer empirical data, parameter-identification procedures, or regularisation assumptions. For this reason, they are not included in the numerical simulations of the present study.

Future research should extend the model through adaptive coupling, radial–angular feedback, cognitive–emotional feedback, noise, learning, plasticity, and empirically grounded parameter tuning. Empirical calibration could combine ecological momentary assessment or other repeated self-reports of valence and arousal with interaction logs and physiological or behavioural recordings, including electrodermal activity, gaze, or movement trajectories. Such data could be used to estimate damping, attractor, and coupling parameters. Predictive validity should then be evaluated using held-out trajectories or other out-of-sample comparisons before the model is used for clinical, behavioural, or intervention-oriented prediction. Data-driven calibration and validation could pave the way for applications of the proposed model in psychotherapy analytics, social robotics, and computational psychiatry.

## Supporting information

S1 Appendix Numerical convergence and solver robustness. (PDF)
