# Supplementary material for: A dynamical systems model of emotional contagion: Oscillatory coupling in the circumplex of affect
Source: PLoS One. 2026 Sep 15;21(9):e0334738. doi: 10.1371/journal.pone.0334738 (PMC13577584; doi:10.1371/journal.pone.0334738)
Supplement: Supplementary file 1 [file pone.0334738.s001.pdf]

A dynamical systems model of emotional contagion: Oscillatory coupling in the circumplex of affect

Diego Alexander Garzón-Alvarado[1,2,4◕], Carlos Alberto Duque-Daza[2,4◕], Alessio Alexiadis[5‡], Ricardo Mauricio Tamayo[3‡],

1 Biotechnology Institute, Universidad Nacional de Colombia, Bogotá, Colombia

2 Research Group in Modelling and Numerical Methods in Engineering - GNUM, Universidad Nacional Colombia, Bogotá, Colombia

3 Department of Psychology, Universidad Nacional de Colombia, Bogotá, Colombia

4 Department of Mechanical and Mechatronics Engineering, Universidad Nacional de Colombia, Bogota Colombia

5 University of Birmingham, Birmingham, United Kingdom

◕These authors contributed equally to this work.

‡These authors also contributed equally to this work.

* caduqued@unal.edu.co

S1 Appendix. Numerical convergence and solver robustness

The proposed model of emotional contagion, formulated through the analogy with coupled harmonic oscillators, is inherently dependent on the method of solution employed. While in theory analytical soluti may be feasible for a few simple and idealised cases, such approaches quickly become cumbersome and impractical as the size and complexity of the network increase. Furthermore, the presence of nonlinearit can make exact analytical solutions unattainable. For these reasons, numerical approaches are not only more convenient but also essential for exploring the behaviour of emotional contagion in realistic scenar

Numerical integration introduces discretisation and interpolation errors; therefore, solver settings m be documented and numerical agreement must be assessed. The purpose of the following test is to quan tolerance convergence and cross-solver consistency for a representative simulation. It is not intended to establish intrinsic sensitivity of the underlying dynamical system.

Numerical convergence was evaluated independently of the density of the stored output grid. In the adaptive `solve_ivp` implementation, `t_eval` determines the times at which the computed solution is reported but does not prescribe the solver's internal time steps.

A representative convergence test was performed using Example 4 with $b_r = b_\theta = 1.0$ over $0 \leq t \leq 5$ This case includes both radial and angular interpersonal coupling and corresponds to the reciprocal three-agent chain considered in the main text. All solutions were evaluated on the same output grid.

The circular angular error was defined as

$$E_\theta = \max_{i,n} \left|\operatorname{atan2}\left(\sin \Delta\theta_i(t_n), \cos \Delta\theta_i(t_n)\right)\right|,$$

where

$$\Delta\theta_i(t_n) = \theta_i(t_n) - \theta_i^{\mathrm{ref}}(t_n).$$

The maximum complex-state and final-state errors were defined as

$$E_z = \max_{i,n} \left|z_i(t_n) - z_i^{\mathrm{ref}}(t_n)\right|,$$

$$E_{z,T} = \max_{i} \left|z_i(T) - z_i^{\mathrm{ref}}(T)\right|.$$

Three numerical configurations were compared: a deliberately looser RK45 solution with `rtol` $=$ `atol` $= 10^{-8}$, the RK45 setting used for the reported Example 4 simulation with `rtol` $=$ `atol` $= 10^{-10}$, and a Radau solution with `rtol` $=$ `atol` $= 10^{-10}$, used for cross-solver compariso (see details in Table 1). All solutions were evaluated on the same output grid.

Table 1. Tolerance convergence and cross-solver comparison for Example 4 with $b_r = b_\theta = 1.0$. Errors evaluated relative to the Radau cross-solver comparison solution.

Method	`rtol`	`atol`	E_θ	E_z	$E_{z,T}$
RK45, loose comparison	10^{-8}	10^{-8}	3.5494×10^{-8}	1.7849×10^{-8}	4.5647×10^{-9}
RK45, reported setting	10^{-10}	10^{-10}	3.3865×10^{-10}	1.6014×10^{-10}	6.0334×10^{-11}
Radau, cross-solver comparison	10^{-10}	10^{-10}	—	—	—

At the RK45 tolerances used for the reported Example 4 simulation, the maximum circular phase discrepancy relative to the Radau comparison solution was 3.3865×10^{-10}, the maximum complex-stat discrepancy was 1.6014×10^{-10}, and the final-state discrepancy was 6.0334×10^{-11}. The deliberately loo RK45 calculation produced larger, but still small, discrepancies. These results demonstrate tolerance convergence and close cross-solver agreement for the representative simulation.

Differences between unconverged numerical solutions are treated as numerical error and are not

interpreted as evidence of chaotic dynamics or sensitive dependence on initial conditions. Establishing intrinsic dynamical sensitivity would require a separate perturbation or Lyapunov analysis, which is outs the scope of the present study.
