# Supplementary material for: A dynamical systems model of emotional contagion: Oscillatory coupling in the circumplex of affect
Source: PLoS One. 2026 Sep 15;21(9):e0334738. doi: 10.1371/journal.pone.0334738 (PMC13577584; doi:10.1371/journal.pone.0334738)
Supplement: Supplementary file 2 [file pone.0334738.s003.pdf]

**PONE-D-25-53517R1**
**A Dynamical Systems Model of Emotional Contagion: Oscillatory Coupling in the Circumplex of Affect**

# Response to Reviewers.

---

## Response to Reviewer 1

We thank the reviewer for the thorough review and constructive comments, which have substantially improved the manuscript. We have addressed each point below and describe the corresponding revisions made in the manuscript. We hope that this revised version responds adequately to these comments.

### Comment 1

*Mapping to valence/ arousal is not explicitly defined and the psychological interpretation is unclear. - The manuscript states that $\theta$ "corresponds to the configuration of valence and arousal", but does not provide an explicit, testable mapping between $(r, \theta)$ and the standard circumplex axes. Readers will expect something like valence = $r \cos\theta$, arousal = $r \sin\theta$. Please define this mapping and keep it consistent across figures. Also define "neutrality" precisely (what is r = 0 in psychological / measurement terms*

**Response.**
We thank the reviewer for identifying the need to make the mapping between the polar model variables and the standard valence–arousal axes explicit. We agree that this mapping is essential for interpretability, reproducibility, and consistency across the figures.

In the revised manuscript, we now define the affective state of agent $i$ through two equivalent representations: a Cartesian circumplex representation and a polar representation. To avoid conflict with the radial velocity variable $v_i(t) = \dot{r}_i(t)$ already used in the dynamical system, we denote valence by $V_i(t)$ and arousal by $A_i(t)$. The mapping is

$$V_i(t) = r_i(t) \cos\theta_i(t), \tag{1}$$
$$A_i(t) = r_i(t) \sin\theta_i(t), \tag{2}$$
$$z_i(t) = V_i(t) + \mathrm{i}A_i(t) = r_i(t) \exp(\mathrm{i}\theta_i(t)), \tag{3}$$

where i is the imaginary unit. Conversely, for $r_i(t) > 0$,

$$r_i(t) = \sqrt{V_i^2(t) + A_i^2(t)}, \tag{4}$$
$$\theta_i(t) = \mathrm{atan2}(A_i(t), V_i(t)). \tag{5}$$

We have also clarified the domain convention. The emotional labels, initial conditions, and attractor coordinates used in this study are normalized with respect to the unit-disk reference scale,

$$\mathcal{D} = \left\{(V, A) \in \mathbb{R}^2 : V^2 + A^2 \leq 1\right\},$$

so that the tabulated reference states satisfy $r_i \in [0, 1]$. In the direct radial baseline, however, $r_i(t)$ is integrated as an unconstrained oscillator coordinate and is not upper-bounded by the governing equation. Nonnegative excursions with $r_i(t) > 1$ are therefore interpreted as model

amplitudes beyond the normalized reference-label scale and are not assigned a discrete circumplex label. Negative values remain inadmissible because they are incompatible with the interpretation of $r_i(t)$ as radial distance. The alternative constrained formulation enforces

$$r_i(t) \in (\varepsilon_r, 1 - \varepsilon_r)$$

whenever strict preservation of the normalized unit disk is required. In both formulations, arousal is represented by the signed vertical coordinate $A_i(t)$, not by the radius $r_i(t)$ itself.

We now define neutrality precisely as

$$r_i(t) = 0 \iff (V_i(t), A_i(t)) = (0, 0). \tag{6}$$

In psychological and measurement terms, this point corresponds to the centered reference value of the affective scale: no net pleasantness or unpleasantness and no net activation or deactivation relative to the chosen measurement baseline. It is therefore a modelling reference state, not a claim that a person has no emotions or that such a state corresponds to a non-human agent. Since $\theta_i(t)$ is mathematically undefined at $r_i(t) = 0$, the angular coordinate is not interpreted at exact neutrality. For empirical or noisy measurements, we define a neighbourhood of practical neutrality as

$$\mathcal{N}_\varepsilon = \left\{ (V, A) \in \mathcal{D} : \sqrt{V^2 + A^2} < \varepsilon \right\}, \tag{7}$$

where $\varepsilon > 0$ is chosen according to the measurement resolution or a predefined sensitivity criterion.

Finally, we have revised the relevant text and figure descriptions to maintain this convention throughout the manuscript. All circumplex trajectories are computed using

$$V_i(t) = \mathrm{Re}[z_i(t)] = r_i(t) \cos\theta_i(t), \qquad A_i(t) = \mathrm{Im}[z_i(t)] = r_i(t) \sin\theta_i(t).$$

We have stated this equivalence explicitly and used it consistently in the interpretation of all circumplex figures. We also revised wording that could conflate radius with arousal: $r_i(t)$ is now consistently described as affective intensity or distance from neutrality, whereas arousal is the vertical coordinate $A_i(t)$.

## Comment 2

*Radial dynamics allow $r < 0$ which is incompatible with radius as intensity. Equations (1-2) treat ri(t) as a state variable. Because r is defined as a radial distance, negative values are conceptually problematic. The manuscript would need to give a constrained formulation ensuring $r_i(t) \geq 0$ or a clear mathematical argument and empirical demonstration that the chosen parameter regimes prevent $r < 0$0. Without addressing this, the core representation z is not well-defined as a circumplex embedding as currently presented.*

**Response.**
We thank the reviewer for this important observation. We agree that, under the circumplex interpretation, $r_i(t)$ represents affective intensity as a radial distance and must therefore remain nonnegative. A negative value of $r_i(t)$ has no psychological interpretation as "negative intensity" and also makes the polar representation non-unique, since

$$r_i e^{\mathrm{i}\theta_i} = |r_i| e^{\mathrm{i}(\theta_i + \pi)} \qquad \text{for } r_i < 0.$$

The direct radial oscillator used in the baseline model does not make the nonnegative half-line positively invariant. Under low damping, strong coupling or forcing, or sufficiently large initial radial velocity, the oscillator can overshoot through $r_i = 0$. We now state explicitly that such

values represent loss of radial admissibility of the unconstrained formulation in that parameter regime. They are not treated as valid circumplex states and are not assigned psychological meaning.

We have retained the direct radial oscillator as the baseline formulation used in the reported numerical examples because it preserves the original mechanical analogy and provides a transparent reference model. We have also introduced an admissibility-preserving alternative for applications in which radial bounds must be guaranteed. In this alternative, an unconstrained latent variable $\rho_i(t) \in \mathbb{R}$ is introduced and the observable radius is defined by

$$r_i(t) = \varepsilon_r + (1 - 2\varepsilon_r)\frac{1}{1 + \exp[-\rho_i(t)]}, \qquad 0 < \varepsilon_r \ll 1.$$

This transformation guarantees

$$r_i(t) \in (\varepsilon_r, 1 - \varepsilon_r)$$

for every finite $\rho_i(t)$, thereby preserving the normalized circumplex domain by construction.

The revised manuscript now clearly distinguishes the two formulations. The direct oscillator remains the baseline used in the reported examples, whereas the constrained formulation is provided as an improved alternative whenever strict radial admissibility is required. For simulations employing the constrained formulation, the numerical procedure advances $\rho_i(t)$, $u_i(t) = \dot{\rho}_i(t)$, $\theta_i(t)$, and $\omega_i(t)$, and radial admissibility is verified through the diagnostic quantities $r_{\min}$ and $r_{\max}$.

Finally, we clarified the status of trajectories produced by the unconstrained radial equation. If this formulation produces $r_i(t) < 0$ under low damping or strong excitation, those values are interpreted as loss of radial admissibility of the unconstrained oscillator; they are not treated as valid circumplex states and are not assigned psychological meaning. In Figure 9, the negative portions of the radial traces for $b = 0.01$ and $b = 0.1$ are now explicitly identified as inadmissible overshoots of the direct radial coordinate. The reported numerical examples continue to use the direct radial baseline, whereas the constrained formulation is presented as an admissibility-preserving alternative for applications that require strict radial bounds.

## Comment 3

*Separation of radial and angular dynamics is assumed but not justified. As written, $r_i(t)$ and $\theta_i(t)$ evolve independently and are only recombined at the end to form $z_i(t)$. In affective science, intensity and direction can interact (high arousal affecting valence dynamics or vice versa). Please justify why separability is appropriate for emotional contagion, or consider adding cross-terms and/ or discussing limitations more explicitly.*

**Response.**
We thank the reviewer for this important observation. We agree that affective intensity and affective direction need not be dynamically independent in real emotional processes. In particular, high arousal, represented in our notation by the signed coordinate

$$A_i(t) = r_i(t)\sin\theta_i(t),$$

may influence transitions in valence, and the valence–arousal quadrant may also modulate the regulation of affective intensity. Therefore, we do not interpret the separability of $r_i(t)$ and $\theta_i(t)$ as a universal psychological or neurobiological claim.

In the revised manuscript, we now state explicitly that the separation between radial and angular dynamics is a first-order modelling assumption. We retained this baseline formulation for three reasons. First, it gives a parsimonious and identifiable reference model, avoiding the introduction of cross-coupling parameters that cannot yet be calibrated from the present

simulation-only study. Second, it allows the radial mechanism, associated with affective intensity, to be interpreted separately from the angular mechanism, associated with affective orientation in the valence–arousal plane. Third, it preserves the mechanical analogy with damped oscillators while allowing network-mediated contagion to act independently on intensity and direction.

We also clarified that separability in the polar dynamical equations does not imply independence of the reconstructed valence–arousal trajectory. Using

$$V_i(t) = r_i(t)\cos\theta_i(t), \qquad A_i(t) = r_i(t)\sin\theta_i(t),$$

the corresponding Cartesian velocities are

$$\dot{V}_i(t) = \dot{r}_i(t)\cos\theta_i(t) - r_i(t)\dot{\theta}_i(t)\sin\theta_i(t), \tag{8}$$

$$\dot{A}_i(t) = \dot{r}_i(t)\sin\theta_i(t) + r_i(t)\dot{\theta}_i(t)\cos\theta_i(t). \tag{9}$$

Thus, even in the separable baseline model, radial and angular motion jointly determine the observable trajectory in the circumplex plane. For example, the same angular velocity produces a larger displacement in the valence–arousal plane when $r_i(t)$ is large than when the state is close to neutrality. Likewise, the effect of radial motion on valence and arousal depends on the current angular position $\theta_i(t)$.

At the same time, we agree that this kinematic coupling is not equivalent to a fully nonseparable affective dynamics. The present baseline does not include terms such as intensity-dependent angular acceleration, arousal-dependent angular drift, or quadrant-dependent radial damping/amplification. We have therefore added an explicit limitation and a possible extension in the manuscript. A general nonseparable version can be written as

$$\dot{r}_i(t) = v_i(t), \tag{10}$$

$$\dot{v}_i(t) = -b_r v_i(t) - [r_i(t) - r_i^*] + \sum_{j\neq i} K_{ij}^r [r_j(t) - r_i(t)] + F_i(t) + \Phi_i^r(r_i, \theta_i, v_i, \omega_i), \tag{11}$$

$$\dot{\theta}_i(t) = \omega_i(t), \tag{12}$$

$$\dot{\omega}_i(t) = -b_\theta \omega_i(t) - \sin(\theta_i(t) - \theta_i^*) + \sum_{j\neq i} K_{ij}^\theta \sin(\theta_j(t) - \theta_i(t)) + \tau_i(t) + \Phi_i^\theta(r_i, \theta_i, v_i, \omega_i), \tag{13}$$

where $\Phi_i^r$ and $\Phi_i^\theta$ represent radial–angular feedback terms. Setting

$$\Phi_i^r = \Phi_i^\theta = 0$$

recovers the separable baseline used in the reported simulations.

We chose not to introduce these additional terms into the present numerical results because doing so would add free parameters and nonlinear feedback mechanisms that require empirical calibration. Instead, the revised manuscript now positions the separable model as a minimal reference framework and identifies nonseparable radial–angular feedback as a direct extension for future work, especially for modelling high-arousal transitions and quadrant-dependent intensity regulation.

## Comment 4

*Novelty/ positioning. The introduction overstates novelty by implying prior work is mainly categorical or static propagation. Please add a stronger related-work section that identifies the closest mathematical relatives and state clearly what is new here.*

**Response.**
We thank the reviewer for this important observation. We agree that the original Introduction

overstated the novelty of the proposed model by implying that previous approaches were mainly categorical or static. This was not our intended claim. Emotional contagion, mathematical affect modelling, and agent-based crowd simulation are all well-established research areas.

To address this issue, we have revised the Introduction and added a concise related-work and positioning subsection. This new text identifies the closest mathematical relatives of the present model, including dimensional and circumplex-based models of affect, dynamical-systems models of emotion, mathematical descriptions of affective processes, and agent-based models of emotional contagion. We now explicitly acknowledge prior work on continuous valence–arousal representations, agent-based emotion contagion, crowd-emotion propagation, and Newtonian or attraction-like emotional dynamics in agent societies.

We have also reformulated the novelty claim. The revised manuscript no longer claims that previous work is primarily categorical or static. Instead, the contribution is positioned more precisely as a synthesis of two mature but only partially overlapping literatures: mathematical models of affective dynamics and multi-agent models of emotional contagion. The specific contribution of the present work is the combination of:

1. a continuous valence–arousal representation based on the circumplex model;
2. a phasor formulation in which affective intensity and affective direction are represented by radial and angular variables;
3. coupled radial and angular oscillator dynamics for multiple interacting agents;
4. directed coupling matrices that encode interpersonal affective influence; and
5. mechanically interpretable dynamical terms associated with damping, baseline regulation, external forcing, and interpersonal susceptibility.

Thus, the revised manuscript frames the model as a mechanically interpretable, circumplex-based, multi-agent dynamical formulation of emotional contagion, rather than as the first model of emotional contagion or the first mathematical model of affect. This revision directly addresses the reviewer's concern and provides a clearer, more accurate positioning of the proposed framework relative to existing work.

## Comment 5

*Coupling matrices $K_r$, $K_\theta$. Key properties are underspecified: directed vs undirected, allowed sign (negative?), normalization/ bounds, static vs time varying and how $K$ is chosen per scenario. The Appendix uses very large couplings (e.g. 50/500) and includes a nonzero diagonal term, which needs explanation. These details are required for interpretability and reproducibility.*

**Response.**
We thank the reviewer for this important observation. We agree that the coupling matrices $K^r$ and $K^\theta$ require explicit specification for interpretability and reproducibility. In the revised manuscript, we have clarified the directionality convention, allowed signs, diagonal entries, scaling, static versus time-varying status, and the rationale for the large numerical values used in the illustrative simulations.

First, we now state explicitly that $K_{ij}$ denotes the influence exerted by agent $j$ on agent $i$. Thus, rows correspond to receiving agents and columns to source agents. The most general formulation is directed, so $K_{ij} \neq K_{ji}$ in general. Symmetric coupling, $K_{ij} = K_{ji}$, is recovered as the special case of reciprocal influence.

Second, the baseline simulations use nonnegative off-diagonal coupling coefficients,

$$K^r_{ij} \geq 0, \qquad K^\theta_{ij} \geq 0, \qquad i \neq j,$$

so that the coupling terms act as attractive or consensus-like mechanisms. In the radial equation, this means that agent $i$ is pulled toward the affective intensity of agent $j$. In the angular equation, it means that agent $i$ tends to align its affective direction with that of agent $j$. We now explicitly identify negative couplings, $K_{ij} < 0$, as a possible extension for repulsive, antagonistic, or polarization dynamics, but such couplings are not used in the simulations reported in this manuscript.

Third, we have clarified the treatment of diagonal terms. In the baseline formulation, the diagonal entries are set to zero,

$$K^r_{ii} = K^\theta_{ii} = 0,$$

because the coupling matrices represent interpersonal influence only. Self-regulation is already represented by the radial and angular attractors, damping terms, and external forcing terms. Moreover, the governing equations use summations over $j \neq i$, so diagonal entries do not contribute to the interpersonal coupling terms. We have therefore corrected the matrix notation in the Appendix so that all displayed coupling matrices have zero diagonal entries.

The coupling matrices used in the reported simulations are not row-normalized, and no universal upper bound is imposed on their off-diagonal entries. Therefore, the total incoming coupling strength,

$$\kappa^r_i = \sum_{j\neq i} K^r_{ij}, \qquad \kappa^\theta_i = \sum_{j\neq i} K^\theta_{ij},$$

may differ across agents and scenarios. The coefficients are consequently interpreted as raw nondimensional coupling gains relative to the restoring, damping, and time-scale parameters of the model, rather than as probabilities or normalized averaging weights. Their practical range must be assessed jointly with the damping and restoring terms, the relevant dynamical time scales, and the solver tolerances, and should be verified through numerical-stability and sensitivity analyses.

Fourth, we have clarified the scaling of the coupling coefficients. The values used in the numerical examples are nondimensional model parameters scaled relative to the restoring terms, damping values, and selected simulation time scale. They are not empirical measurements of interpersonal influence. Values such as 50 and 500 are used in the illustrative scenarios to create clearly differentiated coupling regimes within the chosen nondimensional time scale. This interpretation is now stated in the coupling-matrix subsection of Section 2.2.

Finally, we have clarified that $K^r$ and $K^\theta$ are static within each reported simulation scenario. They are selected to instantiate specific network structures, including directed chains, reciprocal three-agent chains, asymmetric leader–follower chains, weakly coupled networks, and networks with a dominant influencer. A time-dependent extension $K_{ij}(t)$, depending for example on proximity, attention, interaction frequency, or social context, is possible but is not included in the present baseline simulations. We also corrected the descriptions of Figure 3(b), Example 4, and Figure 9 so that each stated topology matches its displayed matrix under the convention that rows represent receiving agents and columns represent source agents.

## Comment 6

*Table 1 emotion coordinates. Table 1 provides polar coordinates used as initial conditions but does not justify their provenance or derivation. Please cite a dataset/ method or clearly label values as illustrative.*

**Response.**

We thank the reviewer for identifying this ambiguity. We agree that Table 1, as originally presented, could be interpreted as reporting empirically or psychometrically established coordinates for the listed emotion terms, although that was not our intention.

No empirical dataset was used to estimate the exact numerical locations in Table 1. The values are illustrative reference coordinates selected by the authors to instantiate representative affective states within the normalized valence–arousal circumplex. Russell's circumplex model provides the conceptual organization of the affective space, but the specific numerical values in Table 1 should not be interpreted as unique normative coordinates derived from Russell's study or from another psychometric dataset.

We have revised Section 2.1 to describe the construction explicitly. With neutrality located at

$$(V, A) = (0, 0)$$

and the positive-valence axis defining $\theta = 0$, representative Cartesian locations were selected in the normalized circumplex schematic and converted to polar form using

$$r = \sqrt{V^2 + A^2}, \qquad \theta = \operatorname{atan2}(A, V).$$

The radial distances were normalized to the unit reference scale, and the angles are reported in both degrees and radians.

We have also clarified the role of these coordinates. They are used to specify illustrative initial states and attractor states in the numerical scenarios; they are not intended as empirically validated or universally fixed placements of the corresponding emotion labels. The decimal precision reported in the table is retained to reproduce the numerical initialization and does not imply equivalent psychometric precision.

To make this distinction unambiguous, we revised the provenance paragraph in Section 2.1, the caption of Figure 1, and the caption of Table 1. All three now explicitly identify the values as illustrative, author-selected reference coordinates rather than dataset-derived estimates.

## Comment 7

*Numerical methods. The numerical methods section is currently underspecified and contains potentially misleading statements. Classical RK4 is a fixed-step method, yet the text mentions "fixed or adaptive step size," without reporting actual step sizes or adaptive tolerances used for each figure. In addition, the manuscript suggests RKF45 is advantageous for stiff regimes; however, explicit RK methods are generally not reliable for stiffness and may require prohibitively small steps. Please report full solver settings ($\Delta t$ or tolerances, simulation horizon, angle wrapping conventions) and include a convergence/robustness analysis (e.g., step-halving or tolerance tightening). The Appendix currently shows outcomes can change markedly with "numerical accuracy"; define what was varied and demonstrate convergence, and separate true dynamical sensitivity from numerical artifacts.*

**Response.**

We thank the reviewer for this careful numerical-methodological assessment. We agree that the original description was underspecified and contained misleading terminology.

Inspection of the computational implementation confirmed that the simulations were performed using the `scipy.integrate.solve_ivp` interface. Because no method argument was supplied, the actual method was SciPy's default `RK45`: an adaptive explicit Dormand–Prince embedded Runge–Kutta pair of orders 5 and 4. The simulations were therefore not computed using a classical fixed-step RK4 scheme. We have corrected Section 2.3 and removed both the classical-RK4 description and the statement that RKF45 is advantageous for stiff systems.

We now report the solver settings explicitly. Most simulations used

$$\texttt{rtol} = \texttt{atol} = 10^{-8},$$

whereas Examples 4 and 5 used

$$\texttt{rtol} = \texttt{atol} = 10^{-10}.$$

The arguments `max_step` and `first_step` were not specified, so the solver selected its internal steps adaptively. We have also clarified that `t_eval` determined only the stored output times and did not represent the internal integration step. The integration horizons and output grids are now reported for the simulation cases.

The angular variables were integrated as continuous, unwrapped real-valued states. Wrapping was applied only after integration for plotting and circumplex interpretation:

$$\theta_i^{\mathrm{wrap}} = [(\theta_i + \pi) \bmod 2\pi] - \pi.$$

The reviewer is also correct that the previous Appendix comparison did not constitute a controlled convergence test. In that comparison, both the number of stored output points and the solver tolerances were varied simultaneously. Because `t_eval` controls output sampling rather than the solver's internal adaptive steps, this comparison did not isolate a numerical-control parameter. We therefore removed the previous Figure 16 and replaced it with a quantitative tolerance-convergence and cross-solver analysis.

The controlled analysis uses Example 4 with $b_r = b_\theta = 1.0$ over $0 \le t \le 50$. We compared a deliberately looser RK45 calculation with

$$\texttt{rtol} = \texttt{atol} = 10^{-8},$$

the RK45 setting used for the reported Example 4 calculation with

$$\texttt{rtol} = \texttt{atol} = 10^{-10},$$

and a Radau calculation with

$$\texttt{rtol} = \texttt{atol} = 10^{-10}$$

for cross-solver comparison. All solutions were evaluated on the same output grid.

Relative to the Radau comparison solution, the looser RK45 calculation gave

$$E_\theta = 3.5494 \times 10^{-8}, \qquad E_z = 1.7849 \times 10^{-8}, \qquad E_{z,T} = 4.5647 \times 10^{-9}.$$

At the RK45 tolerances used for the reported Example 4 simulation, the corresponding discrepancies were

$$E_\theta = 3.3865 \times 10^{-10}, \qquad E_z = 1.6014 \times 10^{-10}, \qquad E_{z,T} = 6.0334 \times 10^{-11}.$$

The qualitative and terminal behaviour was unchanged, demonstrating tolerance convergence and close cross-solver agreement for the representative case.

We have also documented the radial saturation operation used in the numerical implementation. The raw radial state $r_i(t)$ is integrated directly, while the value entering the radial restoring and coupling terms is

$$\widehat{r}_i(t) = \Pi_{[0,1]}(r_i(t)).$$

This saturation does not itself prevent the integrated radial state from overshooting the interval $[0, 1]$.

Finally, differences between unconverged numerical solutions are now treated as numerical error and are not presented as evidence of chaos or intrinsic sensitive dependence on initial conditions. Establishing such behaviour would require a separate perturbation or Lyapunov analysis and is not claimed in the revised manuscript.

## Comment 8

*Conclusions and metrics. Conclusions appear overstated (biological grounding, "predictive reliability") relative to evidence; please moderate language or add validation. At present, the work is best framed as an exploratory computational framework rather than an empirically validated predictive model. Define quantitative outcome metrics for synchronization, convergence/ divergence, "dragging/ resonance" and any claims of chaos.*

**Response.**

We thank the reviewer for this important critique. We agree that some statements in the Abstract, Results, and Conclusions could be interpreted as overstating the biological grounding and predictive status of the proposed model relative to the simulation evidence presented.

We have therefore revised the manuscript to frame the contribution explicitly as an exploratory and hypothesis-generating computational framework. We removed or moderated statements implying empirical, clinical, or neurobiological validation. The revised Abstract and Conclusions now state clearly that the model has not been calibrated against human affective data and should not be interpreted as an empirically validated predictive model. The oscillator and coupling terms are presented as mechanistic analogies whose psychological and biological interpretations require future testing.

We also added an explicit validation roadmap. Future calibration may combine repeated self-report or ecological momentary assessment measurements of valence and arousal with interaction logs and physiological or behavioural recordings, such as electrodermal activity, gaze, or movement trajectories. These data could support estimation of damping, attractor, and coupling parameters. Predictive performance would then need to be evaluated using held-out trajectories or other out-of-sample protocols.

In response to the reviewer's request for reproducible outcome definitions, we added a set of quantitative trajectory-based measures to Section 2.3. Phase synchronization is quantified through

$$R_\theta(t) = \left| \frac{1}{N} \sum_{i=1}^{N} e^{\mathrm{i}\theta_i(t)} \right|,$$

where $R_\theta = 1$ denotes perfect phase alignment. Agreement of the complete affective states is quantified through

$$D_z(t) = \left[ \frac{1}{N} \sum_{i=1}^{N} |z_i(t) - \bar{z}(t)|^2 \right]^{1/2}.$$

Full affective synchronization therefore requires high $R_\theta$ together with low $D_z$.

Convergence and divergence are operationalized by comparing the mean complex-state dispersion in the first and last 10% of the simulation:

$$C_z = \frac{\langle D_z \rangle_{\mathcal{W}_f}}{\langle D_z \rangle_{\mathcal{W}_0} + \varepsilon_m}.$$

Thus, $C_z < 1$ denotes contraction or convergence, whereas $C_z > 1$ denotes increasing dispersion or divergence.

For leader-driven scenarios, we define the mean follower–leader distance

$$D_\ell(t) = \frac{1}{N-1} \sum_{i \neq \ell} |z_i(t) - z_\ell(t)|$$

and the leader-following or dragging index

$$G_\ell = 1 - \frac{\langle D_\ell \rangle_{\mathcal{W}_f}}{\langle D_\ell \rangle_{\mathcal{W}_0} + \varepsilon_m}.$$

Positive $G_\ell$ denotes net movement of the followers toward the leader trajectory.

We also revised the terminology concerning resonance. Because the present study does not perform a periodic-forcing frequency sweep or demonstrate a frequency-response peak, we no longer describe the observed amplitude growth as resonance. We instead use the term *oscillatory amplification*, quantified by the ratio of terminal-window to initial-window root-mean-square oscillation amplitude, $A_{\text{osc}}$.

Finally, we removed positive claims of chaotic dynamics. Terms such as "chaotic partial synchronization" and "chaotic affective trajectories" were replaced by "irregular partial alignment" or "non-convergent trajectories." A claim of chaos would require a dedicated perturbation analysis and evidence of a positive largest Lyapunov exponent under numerically converged integration, which are outside the scope of this study. The revised manuscript therefore makes no claim of chaotic behaviour.

These changes were made in the Abstract, Section 2.3, the opening of Section 3, the discussion and caption of Figures 13 and 14, and the Conclusions and Future Directions section.

## Comment 9

*Presentation and english (minor/ editorial). The manuscript is generally understandable, but it would benefit from editing the conciseness and precision. Please correct specific issues including typographical errors and duplicated paragraphs in the Appendix around Figure 16.*

**Response.**

We thank the reviewer for this editorial observation. We have performed a final proofreading and language-editing pass throughout the manuscript, with particular attention to conciseness, grammatical precision, terminology, typographical errors, and redundant statements.

The duplicated paragraphs previously located in the Appendix around Figure 16 are no longer present. As explained in our response to Comment 7, the former Figure 16 and its associated uncontrolled numerical-accuracy discussion were removed and replaced by a single, concise numerical-convergence and cross-solver analysis.

We also corrected several remaining local issues in the main text, including a duplicated interpretation of the reciprocal three-agent chain, grammatical and punctuation errors in the leader-driven scenario, inconsistent use of the term "resonance," and an unnecessary paragraph introducing an unused expressivity parameter. We consider that these editorial revisions improve readability and precision without changing the mathematical formulation, numerical results, or scientific conclusions of the study.

## Comment 10

*Data/ code availability. Because the findings are simulation-based, full reproducibility requires public access to the code (or complete pseudocode sufficient for reimplementation), all parameter files and initial conditions (including Table 1 values and the coupling matrices used in each scenario), and scripts or raw outputs needed to reproduce figures end-to-end. If these materials are not yet provided in a public repository, I strongly encourage the authors to deposit them and reference them in the Data Availability Statement.*

**Response.**

We thank the reviewer for emphasizing the importance of complete code and data availability for reproducibility. We agree that, because the findings of the present study are simulation-based, the numerical implementation, parameter values, initial conditions, coupling matrices, and figure-generation procedures must be publicly accessible.

We have deposited the complete reproducibility package in a public Zenodo repository with the persistent DOI

`10.5281/zenodo.20742838`.

The repository contains the Jupyter notebooks used for the reported simulation scenarios, all initial and attractor states, the illustrative coordinates reported in Table 1, the radial and angular coupling matrices used in each scenario, the damping and numerical-solver settings, the convergence-analysis code, and the scripts required to regenerate the figures and tables reported in the manuscript.

We have also included a README file that maps each manuscript figure and table to its corresponding notebook, describes the execution order, and documents the required software environment and package versions. No empirical participant-level data were generated or analysed; the results consist of numerical outputs that can be regenerated from the deposited code and parameter specifications.

The Data Availability Statement in the revised manuscript has been updated to identify the repository, its DOI, and the materials contained therein.

---

## Response to Reviewer 2

We thank the reviewer for the constructive assessment of the manuscript and for the specific recommendations concerning reproducibility, presentation, and the interpretation of the mathematical model. The manuscript has undergone substantial revision, and we provide a point-by-point response below.

### Comment 1

*The manuscript does not provide full access to the data and instead states that they are available upon request, which does not satisfy the journal's data policy.*

**Response.**
We thank the reviewer for identifying this issue. The previous "available upon request" statement has been removed. All code and supporting materials required to reproduce the numerical simulations, quantitative analyses, tables, and figures are now publicly available through the Zenodo repository with the persistent DOI

`10.5281/zenodo.20742838`.

The repository contains the Jupyter notebooks used for the simulation scenarios, the initial and attractor states, the emotion-label coordinates, the coupling matrices and damping parameters, the numerical-convergence analysis, and the scripts required to regenerate the reported figures and tables. A README documents the correspondence between the repository files and manuscript outputs, the execution procedure, and the required software environment. No empirical participant-level dataset was generated or analysed in this study; the reported results are numerical outputs that can be regenerated from the deposited code and parameter specifications.

The Data Availability Statement has been revised accordingly.

## Comment 2

*The manuscript contains typographical and stylistic errors, including "yhe" and an incorrectly capitalized "We". The complete manuscript should be proofread.*

**Response.**
We thank the reviewer for this observation. The two specific errors identified by the reviewer have been corrected. We also performed a complete proofreading and language-editing pass throughout the manuscript, focusing on grammatical precision, punctuation, terminology, conciseness, and removal of redundant statements. These editorial corrections did not alter the mathematical model, numerical results, or scientific conclusions.

## Comment 3

*The terms appearing in the principal equations, particularly the emotional-contagion term, are described in a fragmented manner. They should be characterized in the order in which they appear, including both their mathematical and social interpretations.*

**Response.**
We agree that the original presentation was unnecessarily fragmented. The dynamical-model section has been reorganized so that the radial and angular channels are introduced separately and in the same order as their governing equations.

For the radial subsystem, the revised text defines $r_i$, $v_i$, $b_r$, $r_i^*$, $F_i(t)$, and the coupling term

$$\sum_{j \neq i} K_{ij}^r (r_j - r_i)$$

immediately after the radial equations. The coupling term is described mathematically as an attractive diffusive interaction and socially as the influence exerted by agent $j$ on the affective intensity of agent $i$.

The angular variables $\theta_i$, $\omega_i$, $b_\theta$, $\theta_i^*$, $\tau_i(t)$, and

$$\sum_{j \neq i} K_{ij}^\theta \sin(\theta_j - \theta_i)$$

are then defined immediately after the angular equations. The latter term represents directional alignment toward the affective orientations of neighbouring agents.

We also added explicit "Radial dynamics" and "Angular dynamics" headings and relocated Table 1 so that it no longer interrupts the explanation of the governing equations.

## Comment 4

*The roles of the elastic and damping forces should be explained for a non-specialist audience. The interpretation of internal inertia and the restoring term $r_i - r_i^*$ also requires clarification.*

**Response.**
We thank the reviewer for this helpful suggestion. We have added a concise mechanical interpretation aimed at readers without a background in dynamical systems.

The revised radial equation explicitly contains the restoring term

$$-(r_i - r_i^*),$$

which represents endogenous regulation toward the preferred affective-intensity baseline $r_i^*$. The damping term $-b_r v_i$ dissipates radial motion and suppresses persistent oscillations. In the angular channel,

$$-\sin(\theta_i - \theta_i^*)$$

is the periodic analogue of a restoring torque toward the preferred affective direction, while $-b_\theta \omega_i$ damps angular motion.

We have also clarified an important mechanical distinction. Elastic restoration and inertia are not the same phenomenon. Inertia is associated with the second-order character of the equations, whereas the elastic terms restore the state toward equilibrium. The present equations are nondimensionalized with the mass and angular moment-of-inertia coefficients set to unity; consequently, inertia is not varied independently in the present simulations. The term "affective inertia" is therefore used qualitatively to describe persistence or resistance to rapid affective change, rather than as a separately calibrated mass parameter.

## Comment 5

*The spring metaphor would benefit from a clearer explanation. In addition, the meaning and initialization of the external force $\tau_i(t)$ are unclear, and real-life examples should be provided.*

**Response.**
We agree and have expanded the explanation of both the spring–damper analogy and the external inputs.

In the model, $F_i(t)$ and $\tau_i(t)$ represent exogenous influences that are distinct from internal regulation and interpersonal contagion. The radial input $F_i(t)$ changes the distance of the affective state from neutrality, whereas the angular input $\tau_i(t)$ acts as an external torque that biases affective direction in the valence–arousal plane.

For all numerical examples reported in the manuscript, we used

$$F_i(t) = 0, \qquad \tau_i(t) = 0.$$

This choice isolates the effects of the restoring dynamics, damping, and network coupling and resolves the previous ambiguity concerning initialization.

We have also added concrete examples of possible nonzero inputs. A short pulse could represent an acute external event, such as sudden distressing news; a step-like input could represent sustained environmental or social pressure; and a periodic input could represent recurrent exposure, such as repeated media messaging. Events that affect both emotional intensity and orientation may be represented through simultaneous radial and angular inputs. A systematic analysis and empirical estimation of such forcing functions are left for future work.

## Comment 6

*Future applications should extend the model to real-world settings using a priori or real-time measures of emotional influence, enabling prediction of emotional dynamics in everyday social interactions.*

**Response.**
We thank the reviewer for this constructive recommendation and agree that empirical calibration is the necessary next stage.

The revised Conclusions and Future Directions section now outlines a concrete validation pathway. Repeated self-reports or ecological momentary assessment of valence and arousal could be combined with interaction logs and physiological or behavioural measurements, including

electrodermal activity, gaze, and movement trajectories. These data could support estimation of individual attractors, damping coefficients, and interpersonal coupling parameters.

For real-time applications, the static matrices used in the present study could be replaced by time-dependent couplings $K_{ij}(t)$ estimated from proximity, communication frequency, attention, or other interactional signals. Any predictive application would then require evaluation using held-out trajectories or another out-of-sample validation protocol. We have retained the manuscript's explicit statement that the present model is exploratory and has not yet been empirically validated as a predictive model of everyday human affective dynamics.
