# Supplementary material for: A dynamical systems model of emotional contagion: Oscillatory coupling in the circumplex of affect
Source: PLoS One. 2026 Sep 15;21(9):e0334738. doi: 10.1371/journal.pone.0334738 (PMC13577584; doi:10.1371/journal.pone.0334738)
Supplement: Peer review history

# Peer review: Decision Letter 0

**DOI:** 10.1371/journal.pone.0334738.r001

**Authors:** Alberto Greco

22 Dec 2025

Dear Dr. Duque-Daza,

Thank you for submitting your manuscript to PLOS ONE. After careful consideration, we feel that it has merit but does not fully meet PLOS ONE’s publication criteria as it currently stands. Therefore, we invite you to submit a revised version of the manuscript that addresses the points raised during the review process.

Both reviewers find the modeling framework potentially valuable, but Reviewer 1 raises major concerns that must be addressed for acceptance, particularly regarding: (i) the explicit and consistent mapping to the valence–arousal circumplex, (ii) conceptual constraints and interpretability of the model variables (e.g., non-negativity of emotional intensity), (iii) specification and justification of coupling matrices, and (iv) numerical methods, convergence, and reproducibility. These points are required revisions to ensure technical soundness and reproducibility. Reviewer 2’s comments mainly concern clarifications, writing quality, and data/code availability, which should also be addressed.

Please submit your revised manuscript by  Feb 05 2026 11:59PM. If you will need more time than this to complete your revisions, please reply to this message or contact the journal office at plosone@plos.org. When you're ready to submit your revision, log on to https://www.editorialmanager.com/pone/ and select the 'Submissions Needing Revision' folder to locate your manuscript file.

- A letter that responds to each point raised by the academic editor and reviewer(s). You should upload this letter as a separate file labeled 'Response to Reviewers'.
- A marked-up copy of your manuscript that highlights changes made to the original version. You should upload this as a separate file labeled 'Revised Manuscript with Track Changes'.
- An unmarked version of your revised paper without tracked changes. You should upload this as a separate file labeled 'Manuscript'.

If you would like to make changes to your financial disclosure, please include your updated statement in your cover letter. Guidelines for resubmitting your figure files are available below the reviewer comments at the end of this letter.

If applicable, we recommend that you deposit your laboratory protocols in protocols.io to enhance the reproducibility of your results. Protocols.io assigns your protocol its own identifier (DOI) so that it can be cited independently in the future. For instructions see: https://journals.plos.org/plosone/s/submission-guidelines#loc-laboratory-protocols. Additionally, PLOS ONE offers an option for publishing peer-reviewed Lab Protocol articles, which describe protocols hosted on protocols.io. Read more information on sharing protocols at https://plos.org/protocols?utm_medium=editorial-email&utm_source=authorletters&utm_campaign=protocols.

We look forward to receiving your revised manuscript.

Kind regards,

Alberto Greco

Academic Editor

PLOS One

Journal Requirements:

When submitting your revision, we need you to address these additional requirements.

1. Please ensure that your manuscript meets PLOS ONE's style requirements, including those for file naming. The PLOS ONE style templates can be found at

https://journals.plos.org/plosone/s/file?id=wjVg/PLOSOne_formatting_sample_main_body.pdf and

https://journals.plos.org/plosone/s/file?id=ba62/PLOSOne_formatting_sample_title_authors_affiliations.pdf.

2. Please note that PLOS One has specific guidelines on code sharing for submissions in which author-generated code underpins the findings in the manuscript. In these cases, we expect all author-generated code to be made available without restrictions upon publication of the work. Please review our guidelines at https://journals.plos.org/plosone/s/materials-and-software-sharing#loc-sharing-code and ensure that your code is shared in a way that follows best practice and facilitates reproducibility and reuse.

3. Thank you for stating in your Funding Statement:

“This work was supported by Biotechnology Institute - Universidad Nacional de Colombia.”

Please provide an amended statement that declares *all* the funding or sources of support (whether external or internal to your organization) received during this study, as detailed online in our guide for authors at http://journals.plos.org/plosone/s/submit-now.  Please also include the statement “There was no additional external funding received for this study.” in your updated Funding Statement.

Please include your amended Funding Statement within your cover letter. We will change the online submission form on your behalf.

4. Thank you for stating the following financial disclosure:

“This work was supported by Biotechnology Institute - Universidad Nacional de Colombia.”

Please state what role the funders took in the study.  If the funders had no role, please state: "The funders had no role in study design, data collection and analysis, decision to publish, or preparation of the manuscript."

If this statement is not correct you must amend it as needed.

Please include this amended Role of Funder statement in your cover letter; we will change the online submission form on your behalf.

5. We note that the grant information you provided in the ‘Funding Information’ and ‘Financial Disclosure’ sections do not match.

When you resubmit, please ensure that you provide the correct grant numbers for the awards you received for your study in the ‘Funding Information’ section.

6. We note that your Data Availability Statement is currently as follows: [All relevant data are within the manuscript and its Supporting Information files.]

Please confirm at this time whether or not your submission contains all raw data required to replicate the results of your study. Authors must share the “minimal data set” for their submission. PLOS defines the minimal data set to consist of the data required to replicate all study findings reported in the article, as well as related metadata and methods (https://journals.plos.org/plosone/s/data-availability#loc-minimal-data-set-definition).

For example, authors should submit the following data:

- The values behind the means, standard deviations and other measures reported;

- The values used to build graphs;

- The points extracted from images for analysis.

Authors do not need to submit their entire data set if only a portion of the data was used in the reported study.

If your submission does not contain these data, please either upload them as Supporting Information files or deposit them to a stable, public repository and provide us with the relevant URLs, DOIs, or accession numbers. For a list of recommended repositories, please see https://journals.plos.org/plosone/s/recommended-repositories.

If there are ethical or legal restrictions on sharing a de-identified data set, please explain them in detail (e.g., data contain potentially sensitive information, data are owned by a third-party organization, etc.) and who has imposed them (e.g., an ethics committee). Please also provide contact information for a data access committee, ethics committee, or other institutional body to which data requests may be sent. If data are owned by a third party, please indicate how others may request data access.

7. Please update your submission to use the PLOS LaTeX template. The template and more information on our requirements for LaTeX submissions can be found at http://journals.plos.org/plosone/s/latex.

8. Please be informed that funding information should not appear in the Acknowledgments section or other areas of your manuscript. We will only publish funding information present in the Funding Statement section of the online submission form. Please remove any funding-related text from the manuscript.

9. If the reviewer comments include a recommendation to cite specific previously published works, please review and evaluate these publications to determine whether they are relevant and should be cited. There is no requirement to cite these works unless the editor has indicated otherwise.

[Note: HTML markup is below. Please do not edit.]

Reviewers' comments:

Reviewer's Responses to Questions

**Comments to the Author**

1. Is the manuscript technically sound, and do the data support the conclusions?

Reviewer #1: Partly

Reviewer #2: Yes

**********

2. Has the statistical analysis been performed appropriately and rigorously?

Reviewer #1: N/A

Reviewer #2: Yes

**********

3. Have the authors made all data underlying the findings in their manuscript fully available?

The PLOS Data policy

Reviewer #1: No

Reviewer #2: No

**********

4. Is the manuscript presented in an intelligible fashion and written in standard English?

Reviewer #1: Yes

Reviewer #2: No

**********

Reviewer #1: This manuscript proposes a dynamic system model of emotional contagion based on Russell's circumplex model of affect. The affective state of each agent is represented as a complex phasor zi(t), where the radius ri(t) represents the emotional intensity and the angle θi(t) represents the affective orientation in the valence-arousal space. The model combines (i) a radial, damped, driven oscillator with linear diffusive coupling and (ii) an angular, pendulum-like oscillator with sinusoidal coupling, which is solved numerically (RK4 / RKF45) and demonstrated using several network scenarios.

The modeling direction is potentially interesting and could be useful as a generative/simulation framework. However, in its current form, the manuscript has several fundamental problems that prevent an assessment of its scientific soundness and reproducibility, particularly with regard to mapping to circumplex variables, the interpretability of parameters, the construction and scaling of coupling matrices, and numerical verification. The conclusions also overstate the biological basis and predictability compared to what is shown.

1) Mapping to valence/ arousal is not explicitly defined and the psychological interpretation is unclear.

- The manuscript states that θ "corresponds to the configuration of valence and arousal", but does not provide an explicit, testable mapping between (r,θ) and the standard circumplex axes. Readers will expect something like valence = r cos θ, arousal = r sin θ. Please define this mapping and keep it consistent across figures. Also define "neutrality" precisely (what is r = 0 in psychological/ measurement terms?)

2) Radial dynamics allow r <0 which is incompatible with radius as intensity.

Equations (1-2) treat ri(t) as a state variable. Because r is defined as a radial distance, negative values are conceptually problematic. The manuscript would need to give a constrained formulation ensuring ri(t) ≥0 or a clear mathematical argument and empirical demonstration that the chosen parameter regimes prevent r <0. Without addressing this, the core representation z is not well-defined as a circumplex embedding as currently presented.

3) Separation of radial and angular dynamics is assumed but not justified.

As written, ri(t) and θi(t) evolve independently and are only recombined at the end to form zi(t). In affective science, intensity and direction can interact (high arousal affecting valence dynamics or vice versa). Please justify why separability is appropriate for emotional contagion, or consider adding cross-terms and/ or discussing limitations more explicitly.

4) Novelty/ positioning

The introduction overstates novelty by implying prior work is mainly categorical or static propagation. Please add a stronger related-work section that identifies the closest mathematical relatives and state clearly what is new here.

5) Coupling matrices Kr, Kθ

Key properties are underspecified: directed vs undirected, allowed sign (negative?), normalization/ bounds, static vs time varying and how K is chosen per scenario. The Appendix uses very large couplings (e.g. 50/500) and includes a nonzero diagonal term, which needs explanation. These details are required for interpretability and reproducibility.

6) Table 1 emotion coordinates

Table 1 provides polar coordinates used as initial conditions but does not justify their provenance or derivation. Please cite a dataset/ method or clearly label values as illustrative.

7) Numerical methods

The numerical methods section is currently underspecified and contains potentially misleading statements. Classical RK4 is a fixed-step method, yet the text mentions “fixed or adaptive step size,” without reporting actual step sizes or adaptive tolerances used for each figure. In addition, the manuscript suggests RKF45 is advantageous for stiff regimes; however, explicit RK methods are generally not reliable for stiffness and may require prohibitively small steps. Please report full solver settings (Δt or tolerances, simulation horizon, angle wrapping conventions) and include a convergence/robustness analysis (e.g., step-halving or tolerance tightening). The Appendix currently shows outcomes can change markedly with “numerical accuracy”; define what was varied and demonstrate convergence, and separate true dynamical sensitivity from numerical artifacts.

8) Conclusions and metrics

Conclusions appear overstated (biological grounding, "predictive reliability") relative to evidence; please moderate language or add validation. At present, the work is best framed as an exploratory computational framework rather than an empirically validated predictive model. Define quantitative outcome metrics for synchronization, convergence/ divergence, "dragging/ resonance" and any claims of chaos.

9) Presentation and english (minor/ editorial)

The manuscript is generally understandable, but it would benefit from editing the conciseness and precision. Please correct specific issues including typographical errors and duplicated paragraphs in the Appendix around Figure 16.

10) Data/ code availability

Because the findings are simulation-based, full reproducibility requires public access to the code (or complete pseudocode sufficient for reimplementation), all parameter files and initial conditions (including Table 1 values and the coupling matrices used in each scenario), and scripts or raw outputs needed to reproduce figures end-to-end. If these materials are not yet provided in a public repository, I strongly encourage the authors to deposit them and reference them in the Data Availability Statement.

Summary

I believe the modeling idea could be valuable, but major revisions are needed to (i) clearly define the circumplex mapping, (ii) address conceptual constraints such as r ≥ 0, (iii) specify and justify coupling construction/scaling, and (iv) provide rigorous numerical verification and reproducible computational details. Only after these points are addressed can the technical soundness and the strength of the conclusions be adequately evaluated.

Reviewer #2: This manuscript proposes a novel approach for modeling the temporal evolution of affective dynamics during emotional contagion. The authors describe an individual’s emotional state using two independent oscillators, representing emotional intensity and orientation within Russell’s circumplex plane. The study aims to provide the scientific community with a more accurate yet computationally efficient framework to simulate affective dynamics emerging in complex social settings.

While the topic is of interest, the manuscript requires minor revisions concerning the data availability and writing quality. Specifically, in contrast with PLOS One Data policy, the authors do not provide full access to data which are available "upon request" via email. This approach does not meet the journal’s requirements therefore should be revised. Concerning the writing, I found some typographical errors, for instance:

Line 89 : "yhe" instead of "the";

Line 268 : "We" is capitalized incorrectly in the middle of the sentence;

I suggest reading carefully the entire manuscript to identify and correct additional typographical or stylistic issues.

Shifting to methodological aspects, the model in general is intuitive and appropriate for describing affective dynamics as it comprises both internal and external emotional influences. Still, some aspects need further clarification.

Firstly, the elements appearing in Equations (2) and (4) are described in a chaotic and fragmented manner (lines within the range 105–132), especially the term corresponding to emotional contagion. I suggest following the order in which these elements appear in the equations and giving them a complete characterization, all at once. This refers to both mathematical explanation and social interpretation.

Regarding the mathematical framework, to fully understand how the model works, further mathematical contextualization may be useful, especially if the paper is addressed to a non-experts audience such as psychologists. Therefore, it would be helpful to explain what the role of the elastic and damping forces in an oscillator is, and how these translate into the context of affective regulation.

For instance, the concept of internal “inertia” to affective changes, modeled by the elastic force, is very appealing but lacks a proper explanation. Indeed, the model description does not include the term ri-ri*. This aspect should be given greater consideration as it is important for emotional regulation just like external influences. Furthermore, the use of the spring metaphor to illustrate resistance to affective changes is well-chosen and could help make the model even more accessible if accompanied by a clear explanation.

Moreover, the role of the external force τi(t) is not clear: What does It represent in a social setting? Simulations miss initializing this variable and explaining its role, as well. Without real-life examples, it is even harder to understand what this emotional source might represent.

Overall, this paper addresses an interesting and relevant topic, providing simulations of highly diverse social contexts and offering valuable insights for the study of emotional contagion. For future research, the authors may consider extending the application of the model to real-world settings, using either a priori or real-time measures of emotional influences among individuals, which would enable predictions of the dynamics of emotional states in everyday social interactions.

**********

what does this mean?). If published, this will include your full peer review and any attached files.

If you choose “no”, your identity will remain anonymous but your review may still be made public.

**Do you want your identity to be public for this peer review?**  For information about this choice, including consent withdrawal, please see our Privacy Policy

Reviewer #1: **Yes:** Stella Wienhold

Reviewer #2: No

**********

[NOTE: If reviewer comments were submitted as an attachment file, they will be attached to this email and accessible via the submission site. Please log into your account, locate the manuscript record, and check for the action link "View Attachments". If this link does not appear, there are no attachment files.]

To ensure your figures meet our technical requirements, please review our figure guidelines: https://journals.plos.org/plosone/s/figures

You may also use PLOS’s free figure tool, NAAS, to help you prepare publication quality figures: https://journals.plos.org/plosone/s/figures#loc-tools-for-figure-preparation.

NAAS will assess whether your figures meet our technical requirements by comparing each figure against our figure specifications.

# Author comment: Author response to Decision Letter 1

**DOI:** 10.1371/journal.pone.0334738.r002

21 Jul 2026

We thank you and the two reviewers for the careful evaluation and constructive recommendations. We have submitted a clean revised manuscript, a marked version showing the changes, and detailed point-by-point responses to every reviewer comment. Responses have been provided in separate files, but we are in full agreement if a single integrated response file, including all the point-by-point responses, is submitted to the two reviewers.

Attachment Submitted filename: responseToReviewersLetter-PONE-D-25-53517R1.pdf

# Peer review: Decision Letter 1

**DOI:** 10.1371/journal.pone.0334738.r003

**Authors:** Alberto Greco

26 Aug 2026

A Dynamical Systems Model of Emotional Contagion: Oscillatory Coupling in the Circumplex of Affect

PONE-D-25-53517R1

Dear Dr. Duque-Daza,

We’re pleased to inform you that your manuscript has been judged scientifically suitable for publication and will be formally accepted for publication once it meets all outstanding technical requirements.

Within one week, you’ll receive an e-mail detailing the required amendments. When these have been addressed, you’ll receive a formal acceptance letter and your manuscript will be scheduled for publication.

An invoice will be generated when your article is formally accepted. Please note, if your institution has a publishing partnership with PLOS and your article meets the relevant criteria, all or part of your publication costs will be covered. Please make sure your user information is up-to-date by logging into Editorial Manager at Editorial Manager® and clicking the ‘Update My Information' link at the top of the page. For questions related to billing, please contact billing support.

If your institution or institutions have a press office, please notify them about your upcoming paper to help maximize its impact. If they’ll be preparing press materials, please inform our press team as soon as possible -- no later than 48 hours after receiving the formal acceptance. Your manuscript will remain under strict press embargo until 2 pm Eastern Time on the date of publication. For more information, please contact onepress@plos.org.

Kind regards,

Alberto Greco

Academic Editor

PLOS One

Additional Editor Comments (optional):

Reviewers' comments:

Reviewer's Responses to Questions

**Comments to the Author**

Reviewer #2: (No Response)

**********

2. Is the manuscript technically sound, and do the data support the conclusions?

Reviewer #2: (No Response)

**********

3. Has the statistical analysis been performed appropriately and rigorously?

Reviewer #2: (No Response)

**********

4. Have the authors made all data underlying the findings in their manuscript fully available?

The PLOS Data policy

Reviewer #2: (No Response)

**********

5. Is the manuscript presented in an intelligible fashion and written in standard English?

Reviewer #2: (No Response)

**********

Reviewer #2: (No Response)

**********

what does this mean?). If published, this will include your full peer review and any attached files.

If you choose “no”, your identity will remain anonymous but your review may still be made public.

**Do you want your identity to be public for this peer review?**  For information about this choice, including consent withdrawal, please see our Privacy Policy

Reviewer #2: No

**********

# Editor report: Acceptance letter

**DOI:** 10.1371/journal.pone.0334738.r004

**Authors:** Alberto Greco

PONE-D-25-53517R1

PLOS One

Dear Dr. Duque-Daza,

I'm pleased to inform you that your manuscript has been deemed suitable for publication in PLOS One. Congratulations! Your manuscript is now being handed over to our production team.

At this stage, our production department will prepare your paper for publication. This includes ensuring the following:

* All references, tables, and figures are properly cited

* All relevant supporting information is included in the manuscript submission,

* There are no issues that prevent the paper from being properly typeset

You will receive further instructions from the production team, including instructions on how to review your proof when it is ready. Please keep in mind that we are working through a large volume of accepted articles, so please give us a few days to review your paper and let you know the next and final steps.

Lastly, if your institution or institutions have a press office, please let them know about your upcoming paper now to help maximize its impact. If they'll be preparing press materials, please inform our press team within the next 48 hours. Your manuscript will remain under strict press embargo until 2 pm Eastern Time on the date of publication. For more information, please contact onepress@plos.org.

You will receive an invoice from PLOS for your publication fee after your manuscript has reached the completed accept phase. If you receive an email requesting payment before acceptance or for any other service, this may be a phishing scheme. Learn how to identify phishing emails and protect your accounts at https://explore.plos.org/phishing.

If we can help with anything else, please email us at customercare@plos.org.

Thank you for submitting your work to PLOS One and supporting open access.

Kind regards,

PLOS One Editorial Office Staff

on behalf of

Dr. Alberto Greco

Academic Editor

PLOS One
